# Supplementary material for: Glycoproteomic Analysis Reveals Aberrant Expression of Complement C9 and Fibronectin in the Plasma of Patients with Colorectal Cancer
Source: Proteomes. 2020 Sep 22;8(3):26. doi: 10.3390/proteomes8030026 (PMC7564939; doi:10.3390/proteomes8030026)
Supplement: Supplementary file 1 [file proteomes-08-00026-s001.pdf]

Supplementary Materials

Figure S1. Protein patterns from pooled crude plasma samples
Figure S2. Protein patterns from pooled WGA-bound plasma fraction
Figure S3. Protein patterns from pooled WGA-unbound plasma fraction
Figure S4. Total protein gels and immunoblots of C9 from crude plasma, WGA-bound plasma and WGA-unbound plasma fractions obtained from the study cohort.
Figure S5. Total protein gels and immunoblots of FN1 from crude plasma, WGA-bound plasma and WGA-unbound plasma fractions obtained from the study cohort.
Figure S6. Total protein gels and immunoblots of C9 and FN1 from individual samples in the study cohort.
Figure S7. Total protein gels and immunoblots of C9 and FN1 from individual samples in the validation cohort.
Table S1. List of the accepted peptides used in protein quantification.
Table S2. The 80 WGA-enriched plasma proteins selected for protein quantification.
Table S3. List of the 62 WGA-enriched plasma proteins containing at least 2 peptides and their fold change in non-metastatic and metastatic CRC patients compared to those of the healthy controls.
Table S4. N-linked glycosylated searched against 20 WGA-enriched glycoproteins of the pooled sample of healthy control, non-metastatic and metastatic CRC patient groups
Table S5. Glycated peptides searched against 20 WGA-enriched glycoproteins of the pooled sample of healthy control, non-metastatic and metastatic CRC patient groups

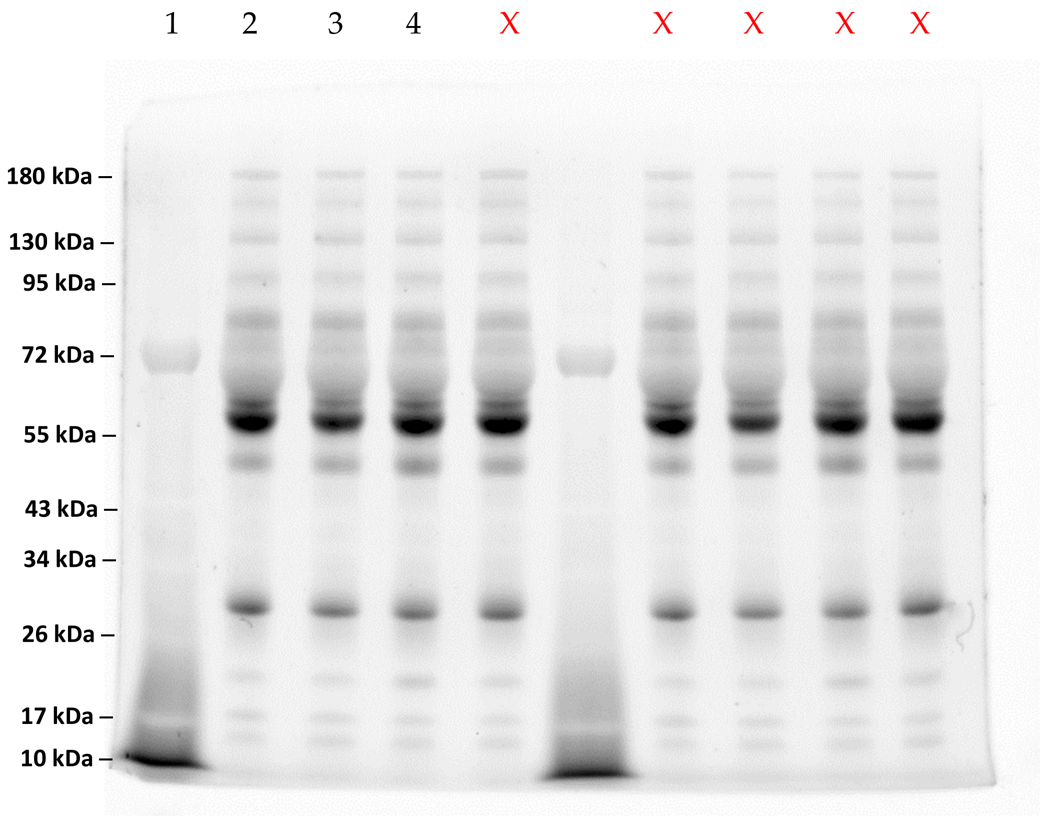

Figure S1. Protein patterns from pooled crude plasma samples
Lane 1: Pre-stained Protein Markers
Lane 2: Pooled crude plasma of heathy controls (10 µg)
Lane 3: Pooled crude plasma of non-metastatic CRC patients (10 µg)
Lane 4: Pooled crude plasma of metastatic CRC patients (10 µg)
X means protein bands from other conditions and authors focus only the proteins in three conditions (healthy controls, non-metastasis and metastasis CRC patients).

Densitometry analysis

Pooled crude plasma samples	Lane 2: Healthy control	Lane 3: Non-metastatic CRC	Lane 4: Metastatic CRC
total protein intensity	11989295	11647984	12549313
Normalized total protein intensity (compared to healthy control)	1.00	0.97	1.05

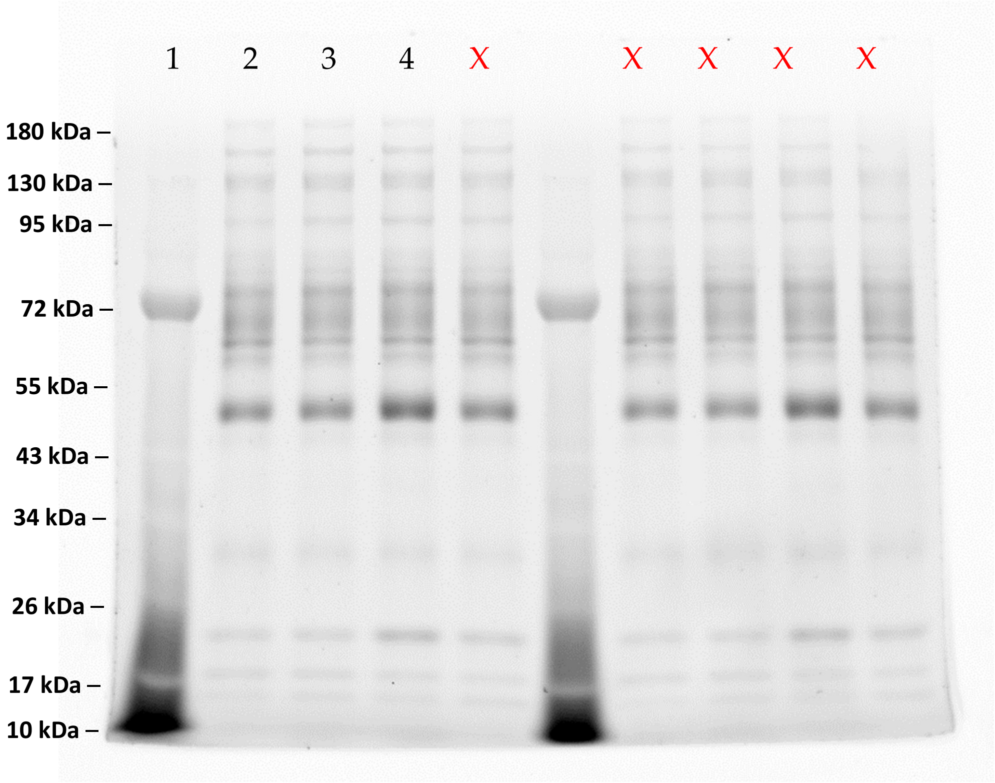

Figure S2. Protein patterns from pooled WGA-bound plasma fraction
Lane 1: Pre-stained Protein Markers
Lane 2: Pooled WGA-bound plasma fraction of heathy controls (5 µg)
Lane 3: Pooled WGA-bound plasma fraction of CRC patients (5 µg)
Lane 4: Pooled WGA-bound plasma fraction of metastatic CRC patients (5 µg)
X means protein bands from other conditions and authors focus only the proteins in three conditions (healthy controls, non-metastasis and metastasis CRC patients).

Densitometry analysis

WGA-bound plasma fraction	Lane 2: Healthy control	Lane 3: Non-metastatic CRC	Lane 4: Metastatic CRC
total protein intensity	9386693	8903096	9999975
Normalized total protein intensity (compared to healthy control)	1.00	0.95	1.07

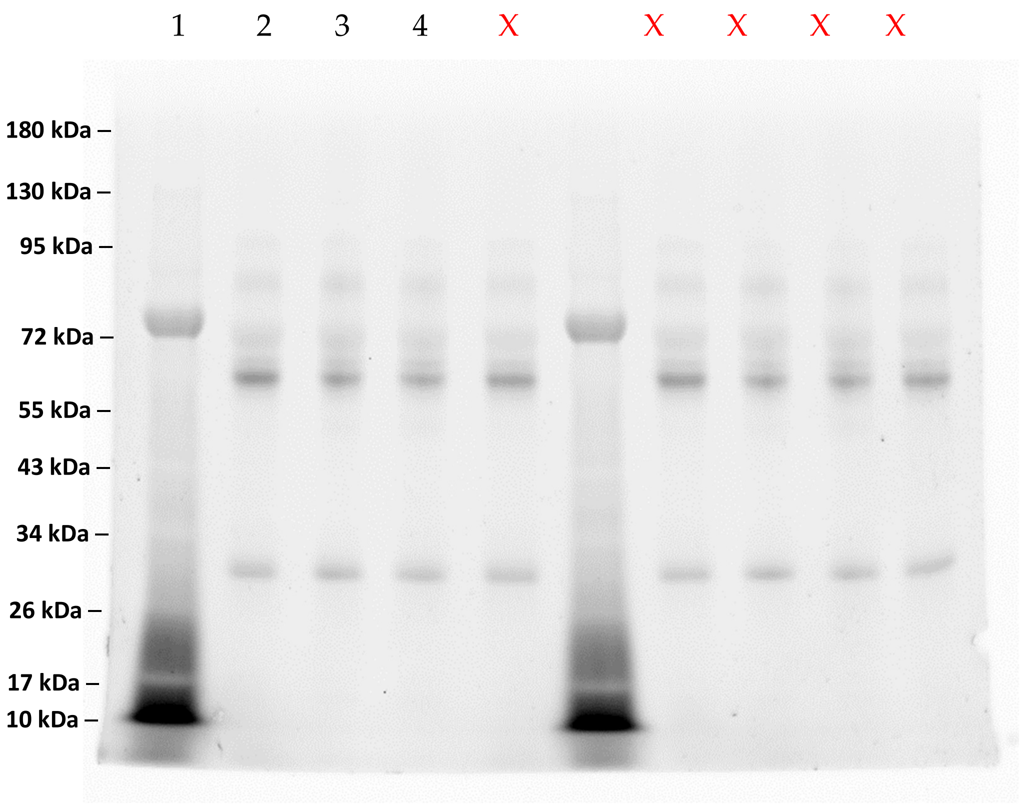

Figure S3. Protein patterns from pooled WGA-unbound plasma fraction
Lane 1: Pre-stained Protein Markers
Lane 2: Pooled WGA-unbound plasma fraction of heathy controls (5 µg)
Lane 3: Pooled WGA-unbound plasma fraction of CRC patients (5 µg)
Lane 4: Pooled WGA-unbound plasma fraction of metastatic CRC patients (5 µg)
X means protein bands from other conditions and authors focus only the proteins in three conditions (healthy controls, non-metastasis and metastasis CRC patients).

Densitometry analysis

WGA-unbound plasma fraction	Lane 2: Healthy control	Lane 3: Non-metastatic CRC	Lane 4: Metastatic CRC
total protein intensity	1925352	1618552	1588311
Normalized total protein intensity (compared to healthy control)	1.00	0.84	0.82

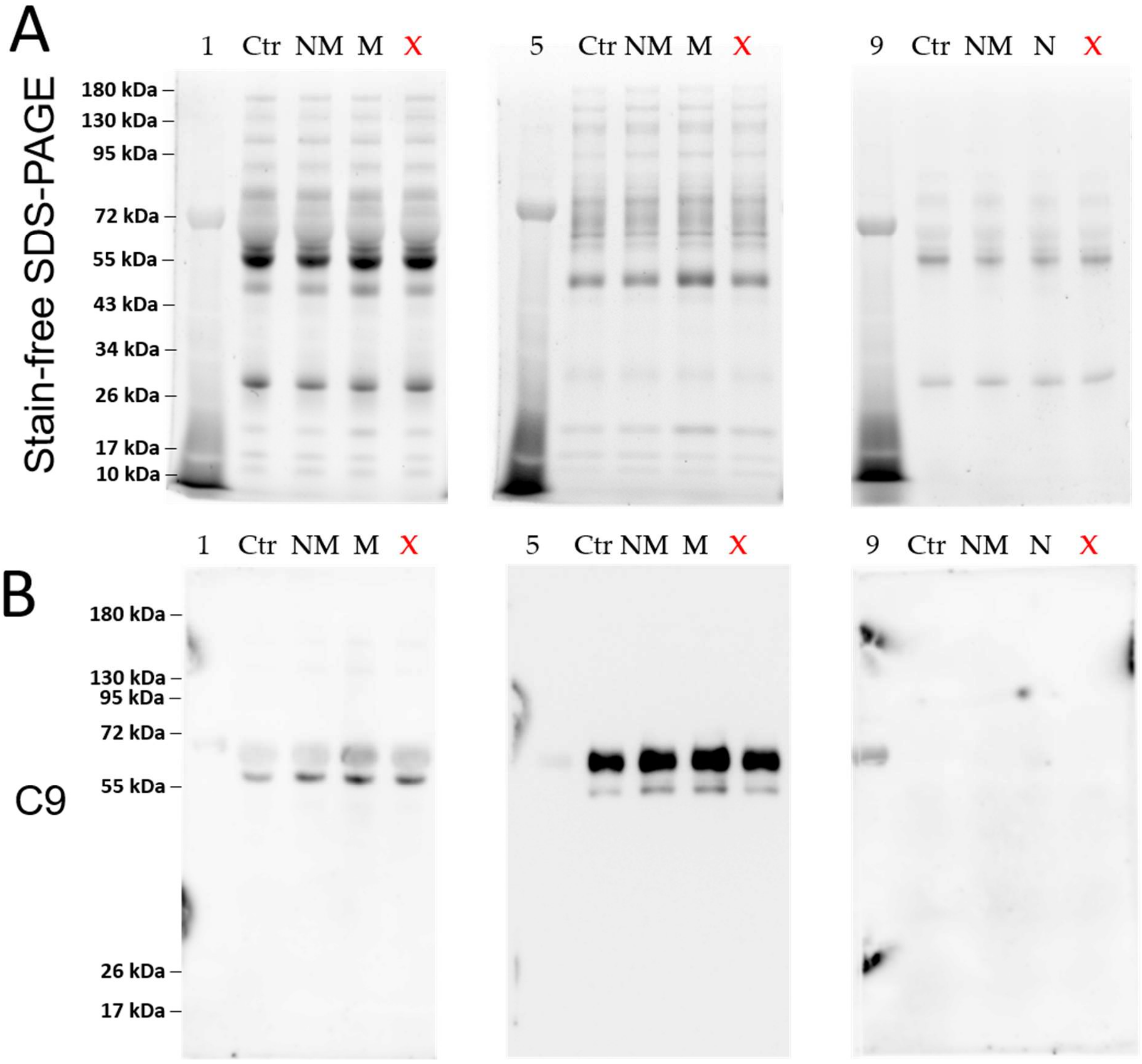

Figure S4. Total protein gels and immunoblots of C9 from crude plasma, WGA-bound plasma and WGA-unbound plasma fractions obtained from the study cohort. (A) Total protein gels performed by 10% stain-free SDS-PAGE. (B) Immunoblots of C9. Crude plasma (10 µg), WGA-bound plasma fraction (5 µg), and WGA-unbound plasma fraction (5 µg) were from healthy control (Ctr), non-metastatic (NM) and metastatic CRC (M) patient groups, respectively. *lane 1, 5, 9: Pre-stained Protein Markers, X means protein bands from other conditions and authors focus only the proteins in three conditions (healthy controls, non-metastasis and metastasis CRC patients).

Densitometry analysis

	Pooled crude plasma (lane 2-4)			Pooled WGA-bound plasma fraction (lane 6-8)			Pooled WGA-unbound plasma fraction (lane 10-12)		
	Crt	NM-CRC	M-CRC	Crt	NM-CRC	M-CRC	Crt	NM-CRC	M-CRC
total protein intensity	11989295	11647984	12549313	9386693	8903096	9999975	1737037	1526846	1652497
C9 intensity (up)	532688	601153	1388285	15989954	22000154	28518073	ND	ND	ND
C9 intensity (down)	554153	1126339	1425823	643454	1890934	2147486	ND	ND	ND
C9 intensity (up+down)	1086841	1727492	2814108	16633408	23891088	30665559	ND	ND	ND
C9/Total protein intensity ratio	0.09	0.15	0.22	1.77	2.68	3.07	-	-	-
Normalized ratio (compared to healthy control)	1.00	1.64	2.47	1.00	1.51	1.73	-	-	-

Note: Crt = Healthy controls, NM-CRC= non metastatic CRC patients, M-CRC= metastatic CRC patients, ND = indicated undetectable.

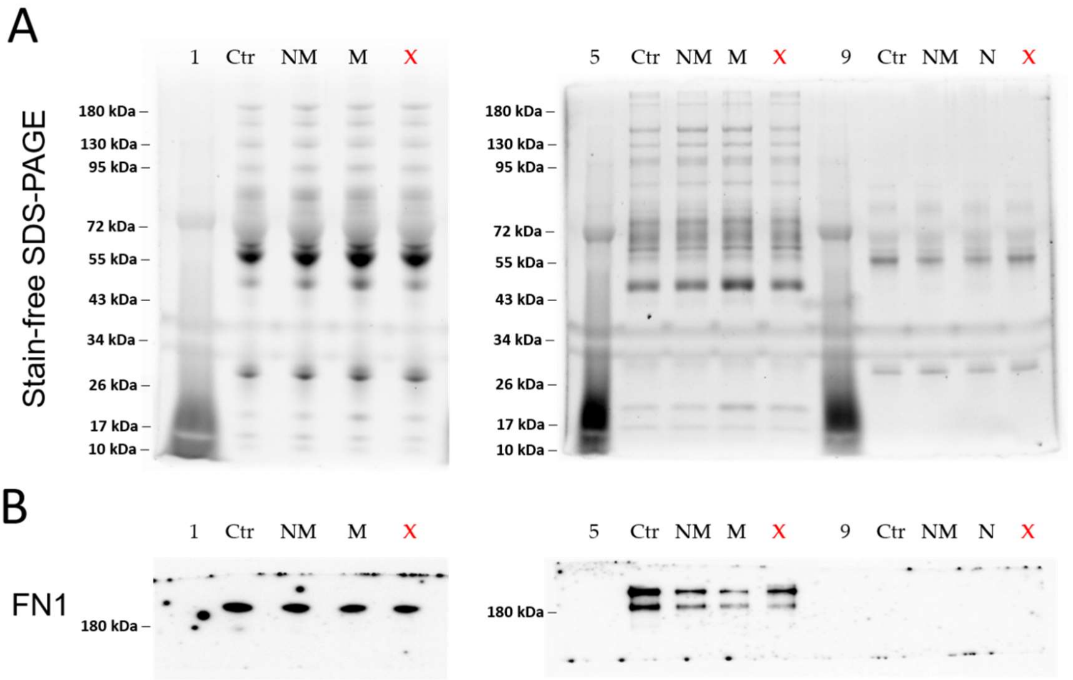

Figure S5. Total protein gels and immunoblots of FN1 from crude plasma, WGA-bound plasma and WGA-unbound plasma fractions obtained from the study cohort. (A) Total protein gels performed by 10% stain-free SDS-PAGE. (B) Immunoblots of C9. Crude plasma (10 µg), WGA-bound plasma fraction (5 µg), and WGA-unbound plasma fraction (5 µg) were from healthy control (Ctr), non-metastatic (NM) and metastatic CRC (M) patient groups, respectively. *lane 1, 5, 9: Pre-stained Protein Markers, X means protein bands from other conditions and authors focus only the proteins in three conditions (healthy controls, non-metastasis and metastasis CRC patients).

Densitometry analysis

	Pooled crude plasma (lane 2-4)			Pooled WGA-bound plasma fraction (lane 6-8)			Pooled WGA-unbound plasma fraction (lane 10-12)		
	Crt	NM-CRC	M-CRC	Crt	NM-CRC	M-CRC	Crt	NM-CRC	M-CRC
total protein intensity	8246249	10105944	11882239	3996026	3896196	4648245	1508204	1337398	1344310
FN1 intensity (down)	15556380	9810423	6456055	23743950	9244336	5041287	ND	ND	ND
FN1/Total protein intensity ratio	1.89	0.97	0.54	5.94	2.37	1.08	-	-	-
Normalized ratio (compared to healthy control)	1.00	0.51	0.29	1.00	0.40	0.18	-	-	-

Note: Crt = Healthy controls, NM-CRC= non metastatic CRC patients, M-CRC= metastatic CRC patients

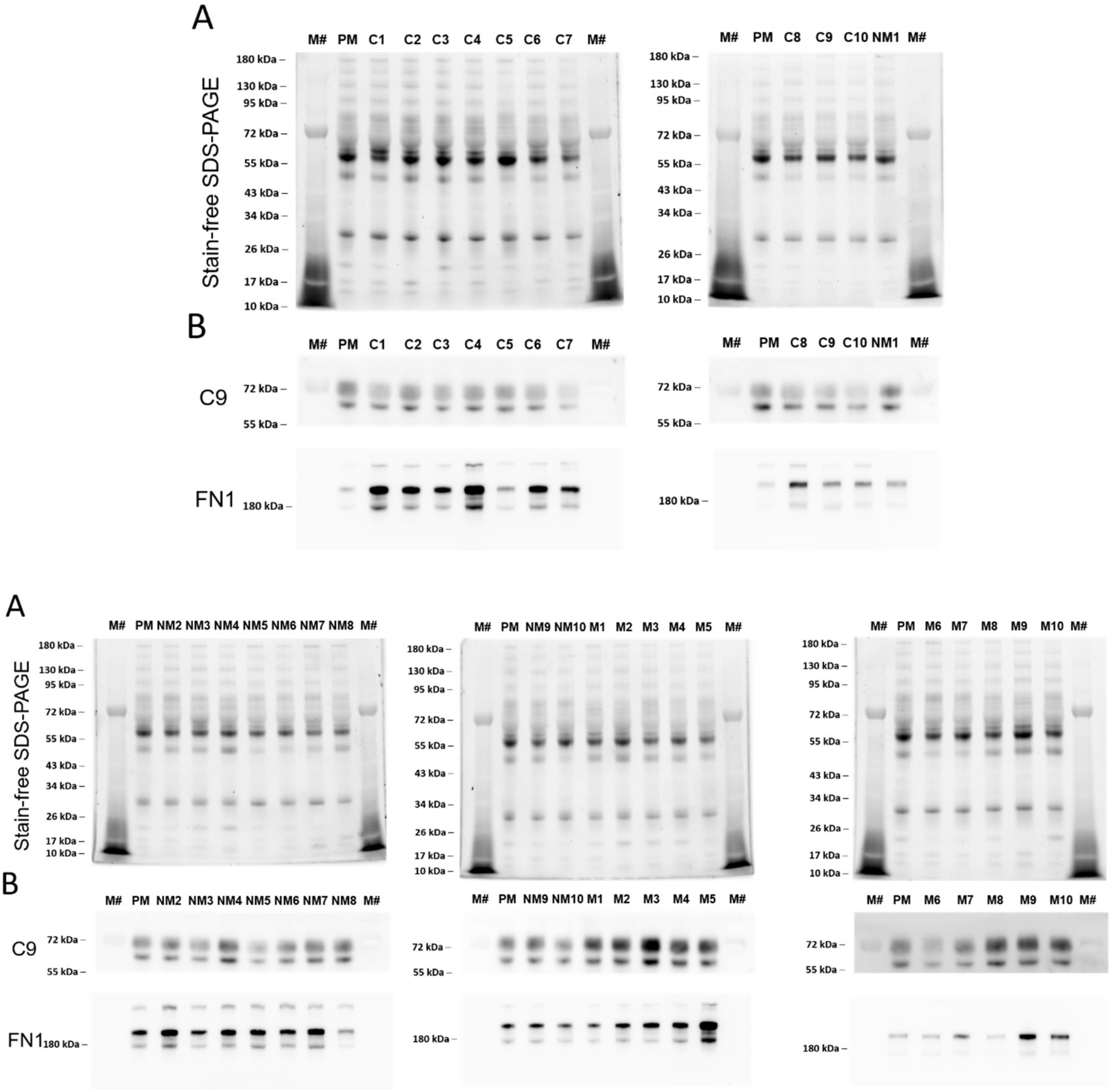

Figure S6. Total protein gels and immunoblots of C9 and FN1 from individual samples in the study cohort. (A) Total protein gels performed by 10% stain-free SDS-PAGE. (B) Immunoblots of C9 and FN1. Healthy controls (C1-C10), non-metastatic CRC patients (NM1-NM10), metastatic CRC patients (M1-M10), PM = a pooled plasma sample for protein normalization of each gel and immunoblot. M# = protein standard marker. Crude plasma samples (10 ug) were loaded in each lane.

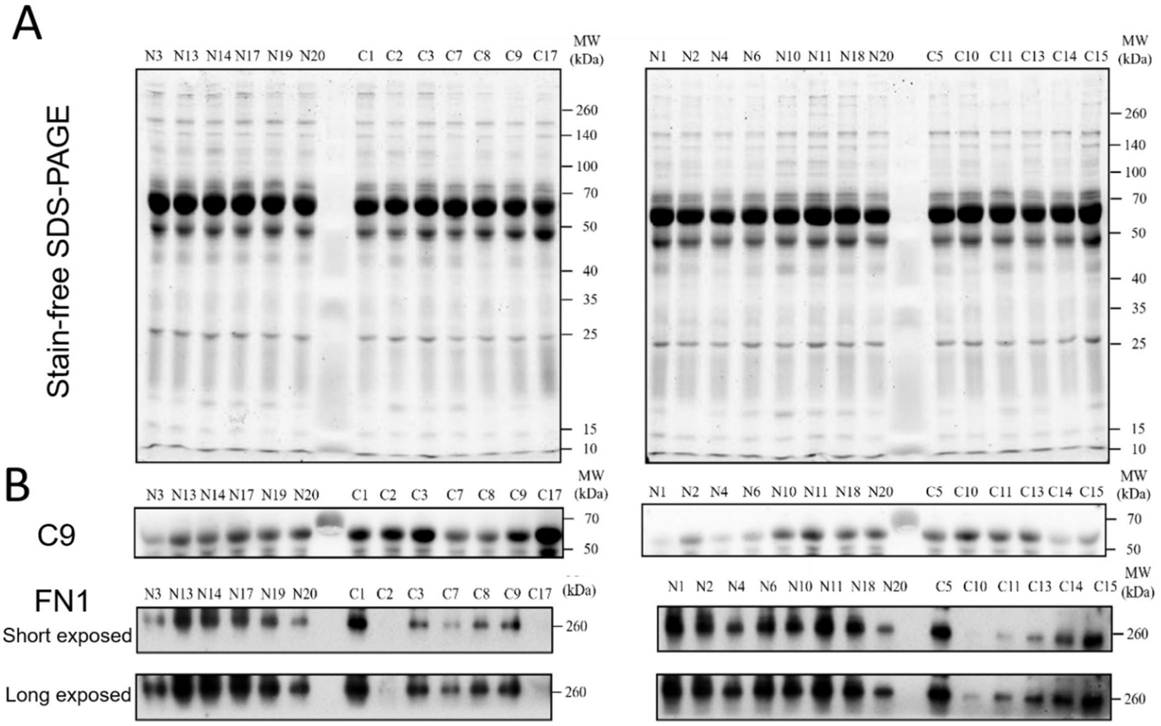

Figure S7. Total protein gels and immunoblots of C9 and FN1 from individual samples in the validation cohort. (A) Total protein gels performed by 10% stain-free SDS-PAGE. (B) Immunoblots of C9 and FN1. Healthy controls (N1, N13, N14, N17, N19, N20, N1, N2, N4, N6, N10, N11, and N18), non-metastatic CRC patients (C5, C10, C11, C13, C14, and C15) metastatic CRC patients (C1, C2, C3, C7, C8, C9, and C17), N20 = a plasma sample for protein normalization of each gel and immunoblot. Crude serum samples (10 ug) were loaded in each lane.

Supplementary Table S1. List of the accepted peptides used in protein quantification

#	Charge	m/z	Measured mass	Score	Sequence	Accession	Anova	Normalized MS peak intensities								
								Healthy control			Non-metastatic CRC			Metastatic CRC		
								Ctr1	Ctr2	Ctr3	NM1	NM2	NM3	M1	M2	M3
7538	2	492.723	983.432	12.71	FSGSSSGAER	sp|A0A075I	0.000162	3865.8	3193.5	3788.6	4469.6	3560.5	4232.2	2182.4	2124.6	1838.6
1399	3	583.243	1746.707	17.06	CSGEEQSLEQCQHR	sp|O43866	0.004865	18313.9	18285.8	17976.4	17704.9	14522.5	16048.8	23182.9	20031.6	20305.1
1482	2	574.281	1146.547	8.01	LVGGDNLCSGR	sp|O43866	0.003399	15880.9	15737.9	15806.1	14516.5	15134.0	12674.3	11564.3	10985.7	10848.8
2728	2	496.295	990.576	3.58	LTPTLYVGK	sp|O75460	1.39E-06	3976.7	3074.3	3786.1	5531.1	5140.3	6221.3	2023.1	1793.4	1786.1
3309	3	349.514	1045.521	7.14	YAVSEAAAHK	sp|O75636	5.70E-06	2824.1	2411.9	2897.9	3012.4	2862.3	2992.2	1509.5	1471.0	1675.6
8547	3	512.218	1533.632	10.07	CNGHASLCNTNTGK	sp|O75882	0.008709	1732.8	1516.0	1840.8	1394.5	1393.1	1417.8	1787.1	1726.6	1967.3
157	2	735.868	1469.720	16.06	EVGPTNADPVCLAK	sp|P00450	0.006089	147701.7	176880.2	176178.2	211575.5	271772.2	196592.2	283706.6	239971.9	296675.0
193	2	686.386	1370.758	15.06	GAYPLSIEPIGVR	sp|P00450	0.015124	96273.4	79784.7	113265.2	129964.5	172923.8	127779.5	116685.8	124635.5	126566.0
294	4	648.281	2589.097	11.02	TYCSEPEKVDKDNEDFQI	sp|P00450	3.00E-06	157220.4	174267.9	154744.7	180311.1	199722.4	177611.1	100278.1	100032.8	101846.5
350	2	760.376	1518.738	15.06	ALYLQYTDETFR	sp|P00450	0.00015	69626.0	66417.7	73291.6	105373.1	123060.6	105837.2	94260.9	81669.4	75469.7
373	3	844.421	2530.240	11.84	SVPPSASHVAPTETFTYE	sp|P00450	0.000321	220695.2	272040.2	236530.3	296223.1	311579.0	306225.9	383860.7	392368.5	334768.2
398	2	470.719	939.424	7.10	YTVNQCR	sp|P00450	0.000258	30616.7	29932.2	27334.6	34449.8	32927.6	38182.1	40756.4	45736.8	43095.0
407	3	638.050	1911.128	11.80	TTIEKPVWLGFLGPIIK	sp|P00450	0.000647	83160.8	91507.1	89746.7	120084.1	145365.1	112004.2	100140.3	95275.1	93275.2
410	4	340.688	1358.722	5.99	AETGDKVYVHLK	sp|P00450	3.99E-05	22945.2	25812.2	23731.7	29708.0	31926.8	30549.7	36147.4	37955.8	34096.3
447	4	854.929	3415.688	9.97	QKDVDKEFYLFPTVFDEN	sp|P00450	0.031199	133433.4	123528.5	140156.7	195928.8	144974.8	177206.0	153536.5	169442.5	160313.4
484	3	835.714	2504.120	16.38	SGAGTEDSACIPWAYYS	sp|P00450	0.000533	139022.6	105574.3	131372.7	177268.2	145654.4	152480.8	97214.3	96334.8	96827.1
637	3	533.271	1596.792	9.55	QYTDSTFRVPVER	sp|P00450	7.83E-05	25486.7	21045.1	25455.4	30265.0	37575.6	28713.4	53610.8	64169.5	70174.6
691	2	735.425	1468.836	18.06	DIASGLIGPLIICK	sp|P00450	6.19E-05	66263.8	63465.9	61794.4	88191.1	78887.0	88595.3	51980.3	55910.9	47432.3
750	3	724.364	2170.069	15.90	LISVDTEHSNIYLQNGPD	sp|P00450	0.000406	52215.3	46280.0	51807.4	64651.6	77047.7	64034.2	49974.8	45566.8	52766.2
753	4	295.909	1179.606	3.15	IYHSHIDAPK	sp|P00450	0.006808	9985.3	9309.0	10519.9	11553.1	11239.3	12403.4	11912.4	11564.8	12368.9
819	3	522.915	1565.723	6.17	VNKDDEEFIESNK	sp|P00450	3.03E-06	29072.2	35965.5	28838.0	44228.3	35795.4	46224.6	9892.4	10467.1	12359.2
822	2	509.236	1016.458	7.34	QYTDSTFR	sp|P00450	5.16E-07	19179.5	18949.8	19595.5	26277.6	29361.5	25477.0	11644.5	11248.3	11287.7
856	3	825.361	2473.062	18.70	MFTTAPDQVDKEDEDFQ	sp|P00450	0.001325	49597.0	58283.6	55872.4	85384.5	120324.5	84651.3	59263.1	51575.7	76608.3
863	4	575.549	2298.165	10.18	MYYSAVDPTKDIFTGLIGI	sp|P00450	6.62E-07	25660.0	26017.8	27318.9	34876.3	33792.2	35803.6	48007.6	48121.3	44880.8
871	4	600.282	2397.097	12.95	HYYIGIIETTWDYASDHGE	sp|P00450	0.027927	39819.7	30630.9	30192.0	49102.2	37498.3	44143.7	45519.9	48464.0	41141.5
875	6	414.728	2482.323	6.97	KAEEEHLGILGPQLHADV	sp|P00450	0.003815	19493.7	24680.9	17533.2	25684.5	26145.6	23327.7	33486.9	29616.0	41838.0
922	4	693.865	2771.431	8.29	ERGPEEEHLGILGPVIWA	sp|P00450	1.94E-05	68007.4	61992.5	66923.0	97181.1	96469.9	94140.4	95941.4	93307.7	82893.5

923	3	444.546	1330.617	3.89	EYTDASFTNRK	sp|P00450	0.00018	14523.5	14001.5	14112.8	19123.1	19757.1	18915.7	17421.4	14698.6	15953.0
964	2	602.269	1202.523	15.02	EYTDASFTNR	sp|P00450	3.02E-06	19880.9	18695.5	21366.5	29239.5	27390.7	27678.6	14256.5	14266.1	15950.5
1083	5	431.821	2154.068	2.44	NLASRPYTFHSHGITYYK	sp|P00450	0.000145	20035.4	21083.5	20804.2	25223.0	26638.7	24898.7	15184.7	15174.0	18514.6
1086	3	765.015	2292.022	12.70	FNKNNEGTYYSPNYNPQ	sp|P00450	2.94E-08	30619.1	31062.6	27607.3	38812.6	43767.3	40736.0	120189.0	111182.1	118612.4
1098	2	788.433	1574.852	18.06	DLYSGLIGPLIVCR	sp|P00450	2.46E-05	55703.4	45286.7	59484.6	88304.4	84971.5	89185.2	40123.0	44836.3	43502.5
1150	2	716.323	1430.632	17.11	QSEDSTFYLGER	sp|P00450	0.016738	29326.7	38321.2	31388.8	38161.4	33346.5	35234.9	35124.5	28373.1	30113.8
1159	3	635.279	1902.816	12.16	NNEGTYYSPNYNPQSR	sp|P00450	0.003528	31857.9	31043.2	29109.2	38160.7	33345.2	36725.8	40012.5	35330.9	37387.9
1168	3	829.768	2486.283	16.17	GPEEEHLGILGPVIWAEV	sp|P00450	0.004874	85554.2	97111.6	80692.4	109733.1	91958.7	116340.0	78181.1	82718.1	76589.9
1251	4	631.793	2523.143	10.56	TYSDHPEKVNKDDEEFIE	sp|P00450	2.73E-05	36293.9	53661.6	36566.3	47570.1	51896.0	49244.7	13351.2	16132.2	16598.8
1326	4	607.272	2425.061	7.64	MFTTAPDQVDKEDEDFQ	sp|P00450	0.000921	26875.2	22967.6	26741.3	30872.0	45802.8	34276.1	20025.2	21129.8	23555.1
1352	3	525.958	1574.852	15.27	DLYSGLIGPLIVCR	sp|P00450	4.92E-05	25272.5	20610.9	27344.9	37281.6	34922.6	36791.8	18420.0	20155.2	19493.0
1408	3	529.919	1586.736	4.08	RQSEDSTFYLGER	sp|P00450	0.000779	14729.7	15928.3	15376.5	19670.4	24154.6	19977.9	17593.3	17822.6	18414.2
1416	5	505.636	2523.144	3.75	TYSDHPEKVNKDDEEFIE	sp|P00450	1.03E-05	22412.3	29704.9	25636.0	32981.1	27859.4	32166.7	9811.8	10414.9	11992.0
1422	3	507.254	1518.739	9.23	ALYLQYTDETFR	sp|P00450	4.18E-05	9792.0	8826.8	10752.5	15711.1	14962.3	14730.2	12970.0	12196.1	11563.4
1437	4	563.547	2250.161	8.52	MYYSAVDPTKDIFTGLIGI	sp|P00450	0.004708	23708.7	25990.0	23327.0	24747.5	27160.7	27302.5	37080.8	39835.9	27136.3
1446	4	575.548	2298.164	7.92	KLISVDTEHSNIYLQNGPI	sp|P00450	0.035185	16377.0	22800.1	20016.9	20562.9	21250.4	19323.3	24591.3	20706.6	24300.8
1447	3	490.619	1468.836	15.27	DIASGLIGPLIICK	sp|P00450	2.31E-05	17477.3	16596.0	17081.4	23750.2	20834.2	23295.3	13537.5	15354.5	13482.6
1497	4	539.524	2154.067	3.39	NLASRPYTFHSHGITYYK	sp|P00450	0.010603	18490.9	24459.7	18661.9	20372.4	30845.7	22696.7	11992.7	12095.4	16640.8
1665	4	632.305	2525.192	11.16	HYYIGIIETTWDYASDHGI	sp|P00450	0.002816	22121.9	23505.9	23825.8	32858.3	47649.3	31828.6	24525.3	26697.8	21431.3
1726	3	800.039	2397.095	19.96	HYYIGIIETTWDYASDHGI	sp|P00450	0.021721	35153.9	27231.3	26748.1	41470.4	32415.6	38866.1	40218.2	40688.2	33157.6
1732	3	457.927	1370.759	9.20	GAYPLSIEPIGVR	sp|P00450	0.004751	6055.0	5633.4	6809.1	7708.6	10293.5	8366.3	7756.0	9303.2	7879.6
1759	5	561.678	2803.353	1.46	WYLFGMGNEVDVHAAFF	sp|P00450	0.000141	17365.2	14478.3	18577.0	24661.6	23788.3	22213.1	14612.1	13787.7	14147.1
1776	5	506.046	2525.192	10.33	HYYIGIIETTWDYASDHGI	sp|P00450	0.001982	15122.1	17045.9	17031.9	23529.5	28081.6	23964.8	17996.1	20169.2	16766.3
1802	4	564.797	2255.160	16.96	KAEEEHLGILGPQLHADV	sp|P00450	0.023705	15478.3	23123.4	16057.7	20171.3	39811.7	21623.2	11053.8	10836.0	15114.1
1896	3	326.151	975.432	6.50	TYSDHPEK	sp|P00450	0.003735	7400.9	6157.0	7482.4	11964.7	8640.1	11481.9	2524.6	4554.9	3351.8
2001	3	533.317	1596.930	11.69	DIASGLIGPLIICKK	sp|P00450	2.07E-06	10192.7	7485.4	9238.5	9634.0	8869.3	10655.2	35670.1	33911.0	29537.6
2235	3	1133.893	3398.657	17.76	QKDVDKEFYLFPTVFDEN	sp|P00450	0.001503	32268.1	32642.6	27144.6	54968.3	45798.9	45636.7	62277.9	46435.7	49322.1
2311	3	864.039	2589.095	16.88	TYCSEPEKVDKDNEDFQI	sp|P00450	1.99E-05	23821.0	24413.9	19613.6	31417.5	37033.6	35641.1	13412.5	12440.9	10499.7
2313	3	905.456	2713.346	18.17	HYYIAAEEIIWNYAPSGID	sp|P00450	3.46E-08	35394.7	37073.0	38415.3	78517.4	58391.9	74426.3	9385.0	9315.0	8460.2
2341	5	775.392	3871.925	11.99	NMATRPYSIHAHGVQTES	sp|P00450	0.000122	16220.5	16614.2	18867.3	30668.5	40787.7	32063.5	34986.5	35399.5	30530.4
2352	3	1054.184	3159.532	18.52	DVDKEFYLFPTVFDENES	sp|P00450	3.78E-06	25069.5	24647.2	26655.6	44632.3	41730.7	41577.2	36821.5	35511.4	31279.8
2427	5	452.039	2255.159	7.16	KAEEEHLGILGPQLHADV	sp|P00450	0.000756	6831.8	8871.9	8232.2	11291.7	15282.4	10625.7	5916.3	4647.2	6968.6
2553	3	890.426	2668.255	20.38	GVYSSDVFDIFPGTYQTL	sp|P00450	4.10E-05	12660.4	9777.6	12690.2	30141.7	23664.8	27360.4	17775.1	17499.0	17402.7
2595	4	575.794	2299.149	3.43	KLISVDTEHSNIYLQNGPI	sp|P00450	0.00707	17328.8	20470.0	15642.0	23048.3	29543.2	22795.0	22902.7	21256.4	26853.3

2861	4	713.847	2851.359	11.79	WYLFGMGNEVDVHAAFF	sp|P00450	7.69E-05	14922.6	13715.7	15340.3	31444.3	23949.4	30665.4	12474.1	11290.6	9275.9
2932	4	850.672	3398.660	12.70	QKDVDKEFYLFPTVFDEN	sp|P00450	3.60E-05	14135.9	15108.1	13798.5	21382.2	18417.4	20970.8	26622.3	25367.3	23492.8
2947	2	1253.067	2504.119	15.17	SGAGTEDSACIPWAYYST	sp|P00450	1.74E-05	37844.7	29004.1	31992.7	54448.7	47723.1	45566.0	23104.6	19161.0	20572.5
3036	2	666.316	1330.617	8.36	EYTDASFTNRK	sp|P00450	0.01025	6011.6	8240.2	5687.1	8877.4	13202.3	9198.6	6591.7	5193.3	5640.0
3112	2	790.890	1579.765	6.02	QYTDSTFRVPVER	sp|P00450	1.43E-06	5507.6	6149.0	5283.8	7278.6	7063.6	6125.5	17493.3	17748.2	15615.9
3225	4	404.200	1612.770	9.40	PYTFHSHGITYYK	sp|P00450	2.17E-07	4917.3	4911.4	4875.2	8329.3	10597.8	7537.1	1485.7	1128.2	1191.2
3407	3	381.886	1142.636	3.98	DIFTGLIGPMK	sp|P00450	0.007279	2574.5	2974.3	2295.8	2867.9	4015.5	2572.1	2438.6	2127.1	1967.2
3428	2	488.723	975.431	6.91	TYSDHPEK	sp|P00450	2.87E-05	7054.1	6113.0	6807.8	11832.5	9056.6	11275.7	2216.3	2111.9	2579.2
3981	5	527.094	2630.432	5.61	IYHSHIDAPKDIASGLIGPL	sp|P00450	4.99E-08	2579.6	2950.3	2961.4	3397.6	4243.6	2937.1	19711.8	19563.2	19858.0
4018	4	1016.243	4060.943	7.07	SGAGTEDSACIPWAYYST	sp|P00450	2.05E-08	18279.8	16056.5	17947.3	21072.6	17440.9	17278.7	93676.4	83432.1	87769.4
4119	3	783.064	2346.169	15.64	MYYSAVDPTKDIFTGLIGP	sp|P00450	3.09E-05	5148.8	5192.0	4397.0	11160.4	9649.7	11229.4	14182.5	10785.5	15034.1
5748	2	956.570	1911.126	13.95	TTIEKPVWLGFLGPIIK	sp|P00450	0.008893	6351.2	8482.4	6905.2	10812.1	17072.1	10431.6	7723.2	7583.5	6742.9
5935	4	790.890	3159.532	12.84	DVDKEFYLFPTVFDENES	sp|P00450	8.20E-05	6000.7	5464.4	6473.1	9903.6	8407.9	8710.5	7556.3	8464.5	6689.3
6142	4	856.658	3422.605	18.90	GDSVVWYLFSAGNEADV	sp|P00450	0.002134	16401.1	15196.0	16102.2	20497.1	16593.6	22702.3	20942.1	22566.5	21099.7
6285	3	924.817	2771.429	18.97	ERGPEEEHLGILGPVIWA	sp|P00450	0.003822	11388.1	9183.0	9295.5	14678.2	17181.2	15680.3	12395.5	11875.0	10280.8
6695	3	1139.570	3415.687	18.71	QKDVDKEFYLFPTVFDEN	sp|P00450	0.002975	7857.6	7197.7	7304.0	14569.7	9782.8	11876.8	8309.3	8839.9	8685.5
7295	4	1016.999	4063.965	20.35	IDTINLFPATLFDAYMVAQ	sp|P00450	1.72E-06	5196.4	5436.6	5440.8	17546.3	14013.7	16061.6	7605.5	8847.8	7988.7
7608	3	1141.875	3422.603	22.69	GDSVVWYLFSAGNEADV	sp|P00450	0.001288	16825.8	16640.2	17356.6	23202.8	19303.4	25826.4	22726.4	23606.5	23587.5
7619	4	622.578	2486.284	6.64	GPEEEHLGILGPVIWAEV	sp|P00450	0.000754	5287.6	5695.8	5689.8	7096.9	6956.0	7876.2	4230.5	5189.1	5160.1
8217	2	596.327	1190.640	8.56	DIFTGLIGPMK	sp|P00450	0.0016	973.8	943.9	1183.1	2040.8	2110.0	1849.3	1523.5	1251.1	1125.0
8311	6	761.910	4565.413	6.84	GPEEEHLGILGPVIWAEV	sp|P00450	1.21E-06	6445.0	7518.9	6370.5	5689.3	6050.0	6809.3	17177.3	15486.6	16145.5
9254	5	552.713	2758.526	11.00	IYHSHIDAPKDIASGLIGPL	sp|P00450	6.50E-08	538.2	753.7	555.3	589.2	739.6	462.6	16161.6	15886.3	11332.6
10046	4	679.344	2713.347	12.43	HYYIAAEEIIWNYAPSGID	sp|P00450	6.40E-08	3507.8	3484.3	3316.9	6283.7	5222.9	6458.7	609.8	563.2	404.2
11846	3	1354.660	4060.959	23.25	SGAGTEDSACIPWAYYST	sp|P00450	3.08E-08	4616.2	4329.5	5590.0	5729.2	4645.8	4679.4	31160.4	27814.8	30420.7
13220	4	996.494	3981.948	16.30	SVPPSASHVAPTETFTYE	sp|P00450	1.38E-06	498.8	1067.3	1002.8	229.0	499.9	563.4	26747.5	31083.4	27557.3
19790	3	901.779	2702.315	18.38	EFYLFPTVFDENESLLLED	sp|P00450	0.000247	911.6	814.3	822.5	1689.2	1409.8	1621.5	469.2	659.6	406.7
27152	2	507.217	1012.418	6.94	TYCSEPEK	sp|P00450	8.65E-06	1858.3	1611.5	1932.6	3116.3	2277.9	3063.8	972.3	893.4	814.7
597	2	574.780	1147.546	9.21	SGIECQLWR	sp|P00734	0.028851	28010.6	36827.5	29160.0	34188.6	40474.3	33500.3	37857.8	39735.5	52662.7
673	3	628.638	1882.891	10.75	TFGSGEADCGLRPLFEK	sp|P00734	2.44E-05	54237.9	61570.8	58557.1	63919.7	71617.6	66314.5	58486.2	53579.3	55712.4
715	3	744.757	2231.250	12.95	GQPSVLQVVNLPIVERPV	sp|P00734	0.017617	65718.2	49739.4	52455.7	71647.0	52049.1	65125.1	78660.6	92722.3	72696.0
733	3	665.689	1994.044	14.90	LAVTTHGLPCLAWASAQA	sp|P00734	0.005189	48927.7	56280.8	45161.0	52753.5	67114.4	47823.1	73541.9	67943.4	59435.1
1108	5	698.375	3486.838	11.52	LKKPVAFSDYIHPVCLPDI	sp|P00734	0.005392	37979.3	35189.8	43928.9	47569.9	66948.3	46327.5	71721.5	62804.4	52450.8
1114	2	597.805	1193.595	15.02	ELLESYIDGR	sp|P00734	1.82E-06	20185.9	20603.1	18650.6	22506.3	25939.1	22191.9	13310.5	13047.6	12305.4
1207	2	781.369	1560.723	15.06	TATSEYQTFFNPR	sp|P00734	0.000127	30925.0	34539.9	29489.8	38679.9	42408.3	36888.5	47832.2	45874.5	39463.8

1243	3	738.010	2211.008	17.64	DKLAACLEGNCAEGLGTI	sp|P00734	0.00283	41330.4	29144.3	35122.8	43335.4	49910.9	39533.4	55913.0	50539.3	60317.8
1288	3	397.199	1188.574	6.76	YGFYTHVFR	sp|P00734	0.001059	9403.6	12005.0	9910.5	10403.8	12570.5	10412.9	12820.4	12140.3	14190.6
1310	2	503.255	1004.495	6.03	ETWTANVGK	sp|P00734	0.002858	9657.1	9060.0	10057.9	12324.0	15581.6	11773.0	13994.1	12136.6	13021.0
1406	2	437.744	873.473	5.28	VTGWGNLK	sp|P00734	0.011407	8091.7	7600.4	8121.4	7809.8	10639.4	9373.1	8654.2	8311.5	9252.0
1946	3	655.309	1962.904	11.67	HQDFNSAVQLVENFCR	sp|P00734	1.86E-08	17628.4	16640.4	18218.0	21921.8	20171.7	23512.5	7841.2	7051.0	7242.4
1951	4	503.754	2010.987	12.44	TFGSGEADCGLRPLFEKI	sp|P00734	6.61E-05	7904.7	10042.8	8175.1	7344.7	10301.4	7562.1	22910.4	21567.9	31607.2
2097	2	486.217	970.419	7.01	YTACETAR	sp|P00734	0.001293	13655.6	11288.9	12688.7	16594.4	13768.0	16259.1	21212.7	20517.5	23653.2
2399	3	521.249	1560.724	6.38	TATSEYQTFFNPR	sp|P00734	0.000139	8075.5	8871.7	8914.8	9879.9	9480.9	9829.2	14234.6	13440.8	11505.2
4868	2	367.683	733.352	1.45	SPFNNR	sp|P00734	0.000686	4983.5	4273.4	5185.5	5817.4	4851.5	5640.6	6536.6	6593.1	6631.4
5568	3	718.986	2153.936	12.12	NPDSSTTGPWCYTTDPT	sp|P00734	2.99E-08	5901.5	6533.1	6029.7	13576.6	11081.2	12598.8	1685.9	1666.7	1292.5
6616	3	493.546	1477.616	10.67	GDACEGDSGGPFVMK	sp|P00734	2.67E-05	3456.4	3620.4	3520.6	4184.2	4233.6	4165.0	2873.6	2923.8	2803.2
8119	3	752.057	2253.148	13.10	TLDEFTIIQNLQPQYQFR	sp|P00736	0.025234	3310.4	2532.0	3086.9	5392.0	3547.2	4150.5	3900.5	4532.2	3831.0
1	2	602.322	1202.630	13.20	VTSIQDWVQK	sp|P00738	7.09E-05	1825986.9	1965019.3	1718245.7	1555540.2	1937719.7	1684234.1	3138003.3	3045356.8	2868703.6
2	2	490.751	979.488	8.00	VGYVSGWGR	sp|P00738	0.000507	826944.5	862480.2	764133.2	687875.2	1028514.4	736483.0	1546695.0	1450848.6	1893256.9
8	2	720.337	1438.659	16.11	TEGDGVYTLNNEK	sp|P00738	0.027277	1051184.7	1109335.4	1101632.6	1121799.2	1308181.8	1065101.5	1279575.2	1314373.9	1639145.5
22	3	570.289	1707.846	10.19	LRTEGDGVYTLNNEK	sp|P00738	0.000159	482534.4	492469.1	547805.8	438020.4	591857.1	526338.2	857333.6	853019.6	933199.8
30	5	687.325	3431.590	6.99	AVGDKLPECEADDGCPK	sp|P00738	9.23E-05	1199447.7	1310342.2	1120443.4	1243566.7	1471497.4	1179491.9	2662172.9	2588919.9	3629565.9
31	4	447.485	1785.911	4.06	VMPICLPSKDYAEVGR	sp|P00738	0.000545	283024.5	357940.8	319038.4	288835.9	424777.8	294246.9	610627.8	632728.5	721567.6
32	6	572.939	3431.593	5.11	AVGDKLPECEADDGCPK	sp|P00738	4.23E-06	969327.2	959696.5	908728.6	991145.3	1047575.2	918697.6	2070501.5	2125199.8	2652680.0
33	3	1292.230	3873.667	22.09	YQEDTCYGDAGSAFAVH	sp|P00738	9.32E-06	3482244.6	3239077.9	3143999.6	4028940.7	3710935.0	3856817.4	5550005.0	5116913.3	5039350.0
43	3	425.551	1273.632	6.77	HYEGSTVPEKK	sp|P00738	0.004037	384038.7	311622.9	370275.8	406284.1	335955.1	397377.7	831590.4	1770137.2	969667.3
47	2	352.191	702.367	1.44	VSVNER	sp|P00738	0.008702	249765.3	219262.7	256573.9	275498.2	212774.1	267568.1	427293.1	449832.9	475048.1
49	3	401.884	1202.631	7.00	VTSIQDWVQK	sp|P00738	3.09E-06	133232.9	132821.7	133419.8	120237.3	127126.9	118400.5	241055.3	261855.3	256299.2
61	4	969.425	3873.670	15.30	YQEDTCYGDAGSAFAVH	sp|P00738	3.78E-06	1831943.2	1780758.1	1890538.1	2146798.8	1941403.8	2086090.2	2862076.7	2911148.0	2890065.4
63	3	553.944	1658.811	6.01	YVMLPVADQDQCIR	sp|P00738	3.93E-05	481706.4	541313.4	467339.8	432720.0	518934.9	495200.6	944455.2	841484.7	1088361.0
65	2	854.414	1706.813	15.11	YVMLPVADQDQCIR	sp|P00738	0.000193	311218.0	426311.1	313176.6	449019.4	564768.9	449920.2	876234.9	928439.0	742977.3
74	4	531.770	2123.050	13.01	SPVGVQPILNEHTFCAGM	sp|P00738	0.000276	282212.1	367348.6	253927.8	246849.0	251856.0	276148.9	570643.6	485264.1	632564.3
81	2	405.194	808.373	3.79	DYAEVGR	sp|P00738	0.002224	105099.4	88249.9	102513.9	127479.2	113173.4	123327.6	91976.6	92877.1	91302.9
95	3	569.946	1706.815	14.27	YVMLPVADQDQCIR	sp|P00738	3.59E-05	150924.3	186056.8	153304.1	205493.4	240604.3	194384.7	390911.9	419634.6	344029.6
98	1	980.495	979.488	3.17	VGYVSGWGR	sp|P00738	0.016542	225576.7	300515.3	180121.1	176137.5	395934.9	218557.1	458731.5	432396.1	749356.0
126	3	596.311	1785.910	12.97	VMPICLPSKDYAEVGR	sp|P00738	0.012628	137762.7	208809.5	170082.6	138522.8	287562.6	166157.9	347468.1	334954.2	447476.7
138	2	854.929	1707.844	12.75	LRTEGDGVYTLNNEK	sp|P00738	0.000959	205712.6	226497.3	213777.7	209061.6	320253.4	251793.7	392674.3	403357.2	427393.0
149	3	599.010	1794.008	14.90	VVLHPNYSQVDIGLIK	sp|P00738	0.005809	140205.9	195832.0	134075.2	120708.9	142188.7	114657.0	251867.1	203994.0	204515.1
160	3	703.680	2108.019	17.70	TEGDGVYTLNNEKQWINI	sp|P00738	3.05E-09	69768.0	94975.2	76622.7	47925.1	50406.0	57345.9	643101.3	597171.3	655516.5

173	2	471.755	941.495	5.94	QKVSVNER	sp|P00738	2.72E-08	33990.5	33606.8	34128.2	42398.2	33383.3	37844.3	143542.6	127050.0	137613.3
174	3	708.690	2123.047	17.95	SPVGVQPILNEHTFCAGM	sp|P00738	0.001495	209460.5	284403.4	183230.2	182497.7	169764.9	190810.5	414877.9	342038.7	561541.8
177	3	320.514	958.521	5.24	QKVSVNER	sp|P00738	0.001992	55857.3	46464.3	55541.3	60922.1	45325.5	57975.8	149710.7	322844.0	194100.4
212	3	480.561	1438.661	7.99	TEGDGVYTLNNEK	sp|P00738	0.000522	71494.0	65483.1	73173.0	76156.1	76691.7	72751.2	89028.5	88143.5	101113.0
221	2	637.823	1273.632	14.71	HYEGSTVPEKK	sp|P00738	0.002683	149157.5	124756.4	160488.7	146981.9	125763.7	181604.7	561305.6	1126028.1	696141.5
246	1	1203.637	1202.630	6.54	VTSIQDWVQK	sp|P00738	1.44E-05	188046.7	210586.3	165396.7	169577.1	214335.9	181154.1	422814.5	420678.5	420118.9
306	3	570.289	1707.845	3.30	LRTEGDGVYTLNNEK	sp|P00738	5.08E-08	28880.1	38089.3	35851.4	21374.4	27599.9	22152.3	218194.9	209318.3	256865.5
335	3	570.617	1708.830	7.79	LRTEGDGVYTLNNEK	sp|P00738	6.64E-05	77506.3	88459.8	79167.9	85311.0	111723.7	90069.3	166988.6	156185.1	154809.4
356	4	858.904	3431.587	19.34	AVGDKLPECEADDGCPK	sp|P00738	0.002593	176699.2	233728.2	162261.9	182761.9	277085.5	182496.7	451398.5	392461.5	685101.1
408	4	319.415	1273.632	5.67	HYEGSTVPEKK	sp|P00738	0.000714	46139.4	39667.2	48024.6	51578.7	42295.9	50858.4	117688.2	187639.5	122814.9
425	2	573.776	1145.537	9.01	HYEGSTVPEK	sp|P00738	0.000606	80338.0	67308.8	61311.4	105651.2	87432.1	91725.4	133821.2	115457.9	168368.7
432	4	595.309	2377.209	7.97	LRTEGDGVYTLNNEKQW	sp|P00738	5.99E-10	15915.5	19870.2	17087.2	13117.6	9949.9	10500.4	261527.7	249111.9	255865.0
438	5	593.276	2961.343	12.83	LPECEADDGCPKPPEIAH	sp|P00738	0.00606	139768.9	145568.9	129539.4	165662.9	191238.3	164798.3	180796.0	170800.5	207071.8
602	2	480.268	958.521	3.65	QKVSVNER	sp|P00738	0.000387	25800.5	20955.1	23733.1	29725.1	21811.7	28573.9	83256.3	221296.9	118004.5
680	2	352.683	703.351	1.43	VSVNER	sp|P00738	0.001237	7887.6	7048.3	8707.4	8851.5	7899.1	9730.4	15936.5	15869.3	13881.7
749	3	382.853	1145.537	3.97	HYEGSTVPEK	sp|P00738	0.002326	27829.7	22874.6	27228.8	32832.6	25446.7	30533.5	54938.5	52764.3	52589.0
844	2	471.754	941.494	7.18	QKVSVNER	sp|P00738	0.000574	20697.0	17109.1	20623.0	23265.6	16851.3	22812.2	65849.6	134189.3	82256.1
929	3	704.009	2109.006	18.64	TEGDGVYTLNNEKQWINI	sp|P00738	1.28E-05	31219.6	26156.5	35863.1	28676.3	29693.3	30559.3	70628.2	77520.9	82219.9
1009	3	389.843	1166.508	3.42	YQCKNYYK	sp|P00738	5.68E-05	8418.2	8136.4	9198.6	9811.1	6825.4	9054.9	21452.6	19293.3	18503.4
1527	4	334.674	1334.665	1.41	NANFKFTDHLK	sp|P00738	6.56E-08	3764.0	4091.7	3608.4	3333.0	4114.0	3360.8	14134.3	13390.1	14726.6
1551	3	793.409	2377.205	16.88	LRTEGDGVYTLNNEKQW	sp|P00738	2.63E-09	4418.4	6854.9	5618.7	4182.0	3784.9	3466.9	104809.5	103809.5	103351.0
1598	4	543.771	2171.054	12.67	SPVGVQPILNEHTFCAGM	sp|P00738	6.32E-05	10282.0	8680.8	10713.1	15144.4	10332.5	13408.7	24514.7	28379.1	28703.0
1607	2	1055.017	2108.019	14.75	TEGDGVYTLNNEKQWINI	sp|P00738	9.16E-07	11371.7	18991.0	9655.1	7692.6	11188.8	9408.8	127341.7	111790.7	132362.1
2065	2	917.964	1833.913	9.02	VMPICLPSKDYAEVGR	sp|P00738	0.001089	13575.7	14910.9	11292.2	18195.1	25545.2	18784.1	36013.5	37870.3	73051.2
2189	4	459.486	1833.915	3.55	VMPICLPSKDYAEVGR	sp|P00738	5.95E-05	5282.1	5898.7	5388.9	6347.7	8726.8	7175.5	15139.0	15755.8	22818.5
2604	2	584.261	1166.508	1.47	YQCKNYYK	sp|P00738	2.68E-05	5096.9	4654.7	5156.1	5730.2	5455.2	6091.8	10891.1	9272.2	9780.5
2638	2	602.324	1202.634	11.67	VTSIQDWVQK	sp|P00738	8.08E-05	6382.2	7579.5	7004.3	6781.9	6785.6	6401.9	11494.8	10139.6	12172.3
2679	3	389.843	1166.508	3.34	YQCKNYYK	sp|P00738	3.38E-08	3393.4	3269.2	3838.8	3383.9	3646.2	3751.8	9759.4	9616.3	9256.2
2686	3	389.515	1165.524	3.67	YQCKNYYK	sp|P00738	1.09E-05	5183.5	4439.6	5306.9	5342.1	4390.7	5005.6	12273.6	12301.1	11798.3
2968	4	532.016	2124.035	10.61	SPVGVQPILNEHTFCAGM	sp|P00738	0.000828	11533.8	11973.3	10558.3	10877.6	14105.3	9688.9	20740.9	18277.8	23496.3
3250	2	352.683	703.351	1.43	VSVNER	sp|P00738	8.01E-05	7901.9	6995.0	8416.9	9133.3	7286.9	8656.4	16666.8	16248.8	16775.2
3716	4	659.378	2633.484	10.83	QLVEIEKVVLHPNYSQVD	sp|P00738	2.60E-09	585.3	561.3	482.9	206.7	208.2	346.0	34393.5	29620.9	30842.3
3808	4	449.509	1794.007	6.72	VVLHPNYSQVDIGLIK	sp|P00738	0.00196	2733.5	4358.8	3226.1	2474.5	3188.6	2623.3	6355.7	5392.1	5555.9
5108	3	320.842	959.506	4.31	QKVSVNER	sp|P00738	0.000122	2442.3	1915.0	2484.4	2778.7	2156.9	2625.7	8059.6	7632.3	8438.4

6128	4	1301.080	5200.292	19.86	YQEDTCYGDAGSAFAVH	sp|P00738	6.85E-11	7489.9	6718.9	5877.6	8295.1	6405.3	8418.6	587627.2	611586.0	555979.3
6136	5	775.741	3873.669	10.09	YQEDTCYGDAGSAFAVH	sp|P00738	0.005964	7203.5	7659.9	10541.6	9752.4	9667.8	9159.7	14912.6	16353.8	13462.7
9004	2	583.770	1165.524	1.44	YQCKNYYK	sp|P00738	0.00448	1563.1	1367.5	1530.1	1737.4	1439.7	1630.7	3861.0	3591.0	4304.6
16091	3	873.160	2616.457	16.97	QLVEIEKVVLHPNYSQVD	sp|P00738	0.001689	65.8	47.0	0.1	49.9	112.7	56.2	8138.5	9346.8	7886.8
27030	3	500.625	1498.854	7.65	SDLGAVISLLLWGR	sp|P00739	0.000879	186.6	156.7	165.9	204.8	243.7	181.9	190.4	239.3	180.6
28762	2	750.434	1498.854	15.06	SDLGAVISLLLWGR	sp|P00739	0.004421	335.0	266.4	266.6	241.7	356.1	318.8	358.0	349.2	241.7
7583	3	677.024	2028.051	7.63	NKPGVYTDVAYYLAWIR	sp|P00748	9.04E-05	2912.1	3270.4	3431.3	3866.9	3749.5	3577.6	2063.9	2029.0	1552.2
3899	2	754.848	1507.681	17.06	VSEADSSNADWVTK	sp|P00751	0.045155	6227.4	8906.0	5907.1	5736.4	6518.2	4941.6	8406.8	7704.5	9933.7
4975	3	939.132	2814.373	17.38	LLQEGQALEYVCPSGFYI	sp|P00751	0.000479	10347.3	9266.7	8831.4	9212.0	8609.8	8542.6	16221.6	15656.6	11922.9
6231	3	326.191	975.552	5.82	VASYGVKPR	sp|P00751	0.008795	838.5	741.9	963.5	634.7	606.4	716.3	880.9	1054.8	1191.0
6902	3	590.664	1768.970	6.77	DFHINLFQVLPWLK	sp|P00751	0.026015	2992.4	2333.0	2735.0	3191.3	2432.8	2920.5	3945.5	4063.1	3282.6
485	3	411.581	1231.721	6.30	ANRPFLVFIR	sp|P01008	0.021803	27002.9	34159.1	30416.7	22271.5	30341.4	22870.2	30515.9	32234.5	35096.5
525	3	447.560	1339.659	9.11	TSDQIHFFFAK	sp|P01008	0.012541	31183.8	37750.5	40149.6	28973.1	30268.1	31153.0	26343.2	30378.7	26584.5
670	2	394.211	786.407	3.89	VANPCVK	sp|P01008	0.04335	14461.4	13081.3	15692.8	12358.7	10250.9	13064.1	16338.8	15949.7	14393.4
986	3	404.553	1210.637	7.46	FRIEDGFSLK	sp|P01008	4.98E-05	15019.0	14701.2	15481.8	11527.2	13715.6	11524.9	7002.7	7031.3	9065.2
1174	2	381.214	760.413	1.40	VWELSK	sp|P01008	0.048319	7674.0	8708.4	7487.7	5564.9	7962.8	6491.6	8808.0	8793.4	9743.4
1361	3	558.954	1673.841	6.51	LQPLDFKENAEQSR	sp|P01008	0.017902	19257.2	27451.6	16189.9	11985.4	16000.9	11346.2	21431.0	22383.3	28808.2
1462	3	764.390	2290.147	15.38	AFLEVNEEGSEAAASTAV	sp|P01008	0.012858	52011.0	46226.2	48148.5	42450.8	38580.6	45660.4	47311.0	47882.2	44630.3
1601	4	605.067	2416.239	10.92	SKLPGIVAEGRDDLYVSD	sp|P01008	0.004379	26501.0	33368.5	29221.9	17127.3	21969.0	18637.6	30535.8	25503.7	32252.6
1960	4	508.500	2029.969	11.29	ATEDEGSEQKIPEATNRR	sp|P01008	3.66E-05	15416.1	13299.7	13722.9	11966.3	11779.1	10965.1	21594.0	21595.6	18544.2
2016	2	459.265	916.515	1.39	RVWELSK	sp|P01008	0.041104	7349.0	9063.9	8889.9	6041.1	6918.4	6137.4	7229.5	6730.0	6713.9
2666	3	600.973	1799.898	7.74	EQLQDMGLVDLFSPEK	sp|P01008	8.12E-08	13856.9	13805.1	15059.0	11581.8	11990.3	11093.7	5104.6	4462.0	4749.5
2936	2	420.212	838.409	5.72	FDTISEK	sp|P01008	1.69E-08	4154.0	3921.4	4531.2	4099.6	4171.1	4240.7	1254.4	1322.4	1340.0
3038	2	350.691	699.367	2.11	SLNPNR	sp|P01008	0.027669	8944.7	7239.4	8267.7	8246.4	5919.8	7120.8	8624.7	8512.6	11095.7
4218	3	326.839	977.495	5.27	FSPENTRK	sp|P01008	0.017651	5213.8	4198.5	5026.4	4753.3	3676.8	4195.2	4654.9	4654.8	4755.1
10435	4	761.139	3040.526	11.51	FRIEDGFSLKEQLQDMGL	sp|P01008	2.06E-06	2196.2	2471.6	2146.0	2551.1	2434.0	2066.8	9191.0	8905.2	7075.3
17601	3	767.049	2298.124	11.28	NDNDNIFLSPLSISTAFAM	sp|P01008	0.001145	1243.4	1087.7	1002.3	1587.1	950.4	1284.6	439.0	361.1	520.7
21601	4	856.433	3421.702	16.52	VEKELTPEVLQEWLDELE	sp|P01008	1.06E-05	692.8	815.9	885.7	488.4	490.4	826.8	3960.6	3491.7	3433.5
24442	4	876.750	3502.972	12.67	ITDVIPSEAINELTVLVLVN	sp|P01008	0.000287	1269.4	1241.1	1026.6	680.3	558.0	635.9	1532.3	1873.0	1268.3
33173	3	935.857	2804.550	7.58	ITDVIPSEAINELTVLVLVN	sp|P01008	0.016346	416.4	434.5	417.1	539.5	447.1	612.1	511.0	498.4	547.0
311	3	360.181	1077.522	5.32	FLENEDRR	sp|P01009	0.030685	28536.5	27043.3	31545.1	29322.9	27444.3	27289.7	30564.7	28218.2	31889.6
415	2	444.756	887.498	6.99	AVLTIDEK	sp|P01009	0.029195	29241.4	42833.0	29392.2	26849.9	40672.3	29048.9	20700.9	20117.7	22293.3
440	2	504.754	1007.494	6.33	QINDYVEK	sp|P01009	4.52E-07	40781.1	48394.8	39636.3	32158.7	37154.5	38892.0	12556.3	13240.0	11527.8
561	3	697.372	2089.093	10.19	ELDRDTVFALVNYIFFK	sp|P01009	0.000394	65984.9	63123.7	55124.6	75108.0	71767.1	68162.7	44650.0	38615.4	39182.7

609	4	334.199	1332.767	4.15	LVDKFLEDVKK	sp|P01009	0.000142	18464.9	22568.2	19105.5	17620.7	26649.2	16935.6	6668.5	7774.6	8349.0
627	3	721.712	2162.114	19.64	VFSNGADLSGVTEEAPLK	sp|P01009	5.90E-09	44757.1	40836.2	43593.7	36490.3	35889.5	33342.8	111899.3	103552.1	100702.2
643	3	284.837	851.488	8.58	SASLHLPK	sp|P01009	0.028255	14024.8	16550.6	14653.8	13469.3	14271.4	13471.2	14853.6	14661.4	13535.5
674	4	547.267	2185.038	12.70	LYHSEAFTVNFGDTEEAK	sp|P01009	5.27E-06	64080.2	76493.0	62610.4	61645.4	75768.2	62701.5	24214.2	21543.0	27642.4
769	3	721.384	2161.129	20.70	VFSNGADLSGVTEEAPLK	sp|P01009	1.43E-05	35588.2	45490.8	35726.8	25634.4	27253.7	22880.1	82321.7	83838.6	69057.8
972	2	821.437	1640.860	15.06	ITPNLAEFAFSLYR	sp|P01009	2.89E-05	72388.0	59057.2	73623.6	75153.6	68189.4	70956.1	42201.5	41190.6	35930.1
981	5	356.760	1778.763	1.44	TDTSHHDQDHPTFNK	sp|P01009	0.001575	16318.3	8158.6	12555.9	18604.7	13957.9	17601.2	5019.3	3352.2	4901.1
997	2	917.958	1833.901	19.95	VFSNGADLSGVTEEAPLK	sp|P01009	1.93E-06	52308.2	62080.0	63586.8	71718.4	91997.9	68755.0	23484.5	23221.8	24053.7
1184	5	630.511	3147.520	11.60	GKWERPFEVKDTEEEDF	sp|P01009	8.14E-05	55316.8	70733.0	54020.1	49517.9	63074.4	49428.8	26245.3	24617.5	26901.6
1196	3	400.538	1198.594	7.00	LGMFNIQHCK	sp|P01009	0.001892	13270.0	11918.4	14596.8	11575.2	11447.0	11166.9	14101.7	14388.0	15547.8
1282	3	858.786	2573.337	19.38	TLNQPDSQLQLTTGNGLF	sp|P01009	2.20E-06	46777.4	42481.0	48117.6	57251.0	48473.8	59664.0	20743.5	19403.2	17925.9
1332	4	445.698	1778.763	4.52	TDTSHHDQDHPTFNK	sp|P01009	0.005134	15251.3	6537.4	11386.8	20073.5	14954.3	17566.9	3880.8	2076.3	3397.6
1336	3	859.115	2574.322	21.38	TLNQPDSQLQLTTGNGLF	sp|P01009	2.62E-06	60302.5	44181.2	56661.7	81816.8	72369.8	76237.4	18870.6	18996.8	14642.4
1355	3	547.961	1640.860	13.27	ITPNLAEFAFSLYR	sp|P01009	6.44E-05	32374.8	25298.0	30169.7	31331.4	25374.1	30059.9	17168.3	16872.8	15220.1
1421	2	917.465	1832.916	17.95	VFSNGADLSGVTEEAPLK	sp|P01009	4.06E-05	36019.4	49042.4	34369.8	38470.2	42616.2	34956.1	14088.9	15170.6	12372.4
1704	4	452.750	1806.970	7.34	FNKPFVFLMIEQNTK	sp|P01009	0.034116	20950.0	33855.4	23714.8	23714.3	34506.4	21115.7	31119.6	34537.4	43132.3
2126	3	612.308	1833.903	15.07	VFSNGADLSGVTEEAPLK	sp|P01009	4.62E-06	13493.3	14264.9	15242.2	16755.8	20533.1	15180.6	6377.8	5881.9	6204.2
2169	3	402.565	1204.673	4.83	LVDKFLEDVK	sp|P01009	0.001324	6051.4	8909.6	6844.4	6080.9	8200.0	5552.2	2764.6	2588.2	3831.5
2411	3	293.505	877.493	1.42	FLEDVKK	sp|P01009	9.71E-11	3766.2	3876.2	3662.4	3946.7	3540.0	3860.4	594.8	546.1	601.1
2503	3	593.928	1778.763	10.47	TDTSHHDQDHPTFNK	sp|P01009	0.003432	10580.7	5124.4	9774.0	13270.5	10868.1	14469.7	3212.0	2019.3	2795.0
2522	3	721.712	2162.114	19.64	VFSNGADLSGVTEEAPLK	sp|P01009	8.54E-06	9259.6	9222.9	9310.6	7531.3	9841.9	7599.6	19214.7	22011.3	19266.7
2587	2	366.209	730.403	3.74	SPLFMGK	sp|P01009	0.002927	12555.4	11105.5	13602.2	11454.9	9777.4	11434.5	16258.7	15829.3	17559.2
2614	4	339.681	1354.695	5.80	RLGMFNIQHCK	sp|P01009	0.008525	4821.8	4242.4	4978.3	4334.4	3816.0	4585.6	4781.3	5093.7	5986.2
2788	3	729.353	2185.036	18.70	LYHSEAFTVNFGDTEEAK	sp|P01009	0.00035	20718.0	25253.5	19568.7	19331.0	25189.3	18171.1	9846.3	8501.4	11481.2
2871	2	538.813	1075.612	9.00	LSSWVLLMK	sp|P01009	0.001236	3766.8	2934.2	3831.6	4817.6	5549.7	5200.7	4989.7	4884.3	4274.9
4197	2	461.718	921.420	2.21	FLENEDR	sp|P01009	0.000152	2017.2	2037.4	2170.8	2095.7	2384.4	2048.4	1705.7	1420.8	1524.0
4247	3	686.654	2056.941	11.49	LYHSEAFTVNFGDTEEAK	sp|P01009	5.55E-05	9004.5	10461.4	9596.2	10670.4	12107.0	9594.3	4126.8	3776.0	5268.9
6612	5	635.911	3174.519	4.19	LYHSEAFTVNFGDTEEAK	sp|P01009	5.43E-07	2140.4	2083.9	1652.0	362.8	288.8	685.6	9962.5	11429.2	9333.7
8152	5	593.488	2962.405	6.29	WERPFEVKDTEEEDFHV	sp|P01009	1.27E-08	4051.3	4412.3	4052.4	4025.3	4432.5	3705.1	722.6	698.4	834.1
8231	4	690.137	2756.520	7.75	IVDLVKELDRDTVFALVNY	sp|P01009	1.81E-10	923.1	753.1	851.9	343.0	378.8	307.3	9913.4	10949.9	9542.6
8830	4	523.280	2089.092	11.41	ELDRDTVFALVNYIFFK	sp|P01009	2.11E-05	1835.4	1526.0	1551.0	1950.2	1826.5	1653.3	884.8	769.6	774.7
26588	2	788.926	1575.837	15.06	DTVFALVNYIFFK	sp|P01009	4.41E-06	629.4	718.3	467.6	781.5	708.9	833.1	110.7	117.0	72.3
35758	4	941.253	3760.982	9.83	TLNQPDSQLQLTTGNGLF	sp|P01009	6.83E-07	61.5	72.8	125.2	33.9	58.3	25.3	1760.2	1876.6	1715.8
206	2	531.298	1060.582	12.98	EIGELYLPK	sp|P01011	0.04515	73347.5	91565.6	98385.2	91169.8	93143.3	99286.6	104754.8	117378.4	108341.8

298	3	370.190	1107.548	3.45	WRDSLEFR	sp|P01011	0.009167	22483.0	31333.0	24821.7	22781.1	34051.2	26850.3	40518.6	42787.1	47951.0
308	3	365.549	1093.626	8.57	NLAVSQVVHK	sp|P01011	0.001561	32597.5	31690.7	30820.1	35719.9	37835.8	32242.6	38776.5	40097.4	45254.1
480	2	432.746	863.477	1.45	LINDYVK	sp|P01011	0.020863	18660.7	18753.5	20361.3	21117.4	24465.1	21098.1	24758.5	24074.1	30271.4
619	3	636.659	1906.956	18.11	AVLDVFEEGTEASAATAV	sp|P01011	0.009607	52870.3	44727.5	51838.9	68426.1	62227.7	59489.2	46098.8	49379.8	38491.5
716	4	455.994	1819.945	4.95	EQLSLLDRFTEDAKR	sp|P01011	1.35E-06	19526.0	21724.7	23829.3	18930.7	19725.6	19237.9	38598.4	38472.3	40496.8
944	2	383.691	765.368	1.86	DSLEFR	sp|P01011	0.00043	8609.4	9512.6	8884.8	9309.2	12571.1	10595.6	6594.9	6125.0	5653.3
996	2	351.724	701.433	2.14	ITDLIK	sp|P01011	3.82E-06	8820.2	11998.7	9319.8	10705.9	14919.7	11598.5	3071.0	2947.1	3413.7
1107	2	954.484	1906.954	19.90	AVLDVFEEGTEASAATAV	sp|P01011	0.003627	52170.8	51535.6	50160.2	76385.3	65840.4	60038.2	48957.8	46363.6	38625.5
1686	3	887.132	2658.374	12.16	FNRPFLMIIVPTDTQNIFFI	sp|P01011	0.000199	23667.0	22692.4	23710.6	57806.8	42341.8	54591.8	34550.5	33478.0	27810.9
1821	3	405.916	1214.726	7.80	ITLLSALVETR	sp|P01011	0.024085	4975.1	3728.3	4656.9	7014.4	5095.8	6436.9	5587.8	6001.0	4973.9
1874	3	742.381	2224.121	15.90	GTHVDLGLASANVDFAFS	sp|P01011	1.95E-07	40512.7	41288.4	38267.3	61855.4	54285.7	51736.8	15461.9	16011.7	12753.8
2006	3	607.656	1819.945	10.72	EQLSLLDRFTEDAKR	sp|P01011	8.41E-05	9111.7	10985.3	11085.3	8477.7	10096.3	9275.6	18976.4	16189.1	19990.1
2315	2	711.346	1420.677	14.06	DEELSCTVVELK	sp|P01011	1.22E-05	9635.1	11220.0	9611.8	12866.2	15408.3	13512.7	4952.9	4522.9	3336.8
2840	4	544.773	2175.064	10.15	RLYGSEAFATDFQDSAA	sp|P01011	6.44E-05	5412.6	7618.0	7370.4	6247.2	5513.3	6093.9	13530.9	12646.5	15191.3
4316	2	1148.590	2295.166	17.90	DYNLNDILLQLGIEEAFTS	sp|P01011	0.010748	11224.8	9545.4	11028.8	22820.1	13666.9	15742.9	12011.0	9148.0	8910.6
5454	2	355.672	709.330	3.36	FTEDAK	sp|P01011	0.002804	2797.2	2369.3	2963.9	3662.6	3034.3	3479.7	1982.4	1979.2	1769.4
5700	3	916.486	2746.437	14.91	ITDLIKDLDSQTMMVLVNY	sp|P01011	0.001404	7401.4	6298.3	7870.8	11231.5	10125.7	10841.1	10917.4	10414.3	9315.6
8214	4	665.601	2658.375	12.28	FNRPFLMIIVPTDTQNIFFI	sp|P01011	6.09E-05	2140.9	1957.5	2162.1	4731.4	3775.6	4547.5	3123.3	3022.9	2602.7
11037	4	687.617	2746.437	9.79	ITDLIKDLDSQTMMVLVNY	sp|P01011	0.001392	1986.2	1754.3	2159.5	3063.6	2660.0	2694.6	2908.8	2716.2	2543.6
18606	3	766.391	2296.152	17.11	DYNLNDILLQLGIEEAFTS	sp|P01011	2.06E-05	1231.4	1100.3	1397.4	2351.6	2046.1	2604.2	952.3	809.4	906.5
19027	4	733.896	2931.554	6.83	GKITDLIKDLDSQTMMVL	sp|P01011	1.87E-05	761.0	832.2	1033.6	674.9	568.7	813.8	3015.5	3124.2	2435.6
23935	3	1079.894	3236.661	21.44	DYNLNDILLQLGIEEAFTS	sp|P01011	1.47E-06	473.9	282.6	496.3	335.2	391.5	625.8	6492.1	6317.8	6870.0
26335	4	810.173	3236.662	12.27	DYNLNDILLQLGIEEAFTS	sp|P01011	4.99E-08	201.0	146.4	241.3	172.1	161.8	170.3	3452.7	2793.3	2895.5
2042	3	607.980	1820.919	1.47	SLDFTELDVAAEKIDR	sp|P01019	0.000884	12772.3	13198.2	13959.8	9280.8	7944.4	8661.5	11186.0	10944.8	8205.4
3582	3	575.014	1722.019	15.11	VLSALQAVQGLLVAQGR	sp|P01019	1.30E-07	10140.9	7893.8	9886.6	9099.9	6493.8	8299.7	660.4	933.6	826.2
3789	3	822.464	2464.371	20.22	ADSQAQLLLSTVVGVFTA	sp|P01019	1.78E-06	19535.2	14816.8	17398.1	14998.4	10668.6	11893.3	3250.0	3126.2	3618.5
4173	3	633.369	1897.087	7.23	QPFVQGLALYTPVVLPR	sp|P01019	1.44E-06	8596.6	7269.5	9287.9	7096.3	8402.1	6996.3	2892.7	2898.2	3084.4
8584	4	1067.832	4267.297	21.51	TIHLTMPQLVLQGSYDLQ	sp|P01019	0.011823	12172.1	11249.4	11359.6	11861.9	11080.6	11658.6	15730.4	13436.8	16970.5
8770	4	617.101	2464.373	19.38	ADSQAQLLLSTVVGVFTA	sp|P01019	6.00E-06	4157.6	3016.6	3801.5	3051.6	2318.3	2395.9	801.3	611.0	707.3
13044	6	1085.393	6506.314	4.06	VGEVLNSIFFELEADEREI	sp|P01019	0.000251	6570.5	7020.1	7637.3	4560.8	3727.8	4215.2	8429.0	8997.0	6657.8
24	2	628.326	1254.637	12.89	AIGYLNTGYQR	sp|P01023	0.020008	472502.9	558501.5	480847.5	454616.8	468112.4	465249.9	612050.7	547767.0	683834.5
77	3	558.292	1671.855	13.06	TEHPFTVEEFVLPK	sp|P01023	0.000173	282200.1	243858.1	298641.1	291417.0	289229.7	288907.9	195031.8	169468.1	162044.1
89	2	697.844	1393.673	17.11	NEDSLVFVQTDK	sp|P01023	3.27E-07	412859.8	424119.8	399517.1	334063.7	402005.0	354129.4	156857.2	157389.6	145813.7
92	2	669.830	1337.645	14.06	NALFCLESAWK	sp|P01023	1.96E-05	214528.3	300793.9	246436.1	226679.3	208845.4	221727.6	107561.8	113071.3	103667.4

97	3	796.405	2386.192	22.17	QQNAQGGFSSTQDTVVA	sp|P01023	0.017023	496861.3	521419.1	526125.6	462746.0	520903.0	438950.2	365735.7	360518.9	446848.9
117	2	324.216	646.418	1.40	VIFIR	sp|P01023	2.08E-05	58698.4	68888.2	55807.5	60669.2	74612.7	59054.7	25917.6	24130.6	30265.2
129	2	523.799	1045.583	12.80	FEVQVTVPK	sp|P01023	2.00E-06	158710.5	181227.6	177421.4	150275.5	149012.5	145838.8	85581.5	88432.4	96191.1
130	2	924.944	1847.874	20.95	QFSFPLSSEPFQGSYK	sp|P01023	0.000838	449561.7	544526.3	430636.5	409137.4	330747.4	409373.4	288905.2	271401.2	273696.0
133	3	633.966	1898.876	14.90	FSGQLNSHGCFYQQVK	sp|P01023	4.43E-05	275374.6	307433.4	265326.5	297025.0	327314.7	286150.6	171653.2	180206.0	192780.8
134	2	574.815	1147.616	11.79	QGIPFFGQVR	sp|P01023	5.12E-05	222644.7	227197.0	226141.5	232393.8	206036.4	215243.9	134427.5	149341.3	120806.0
143	2	503.739	1005.464	3.73	FQVDNNNR	sp|P01023	9.08E-05	102201.5	91251.4	110804.9	105048.9	88535.5	106384.5	58261.0	53964.6	53649.2
145	2	414.733	827.451	6.76	SDIAPVAR	sp|P01023	1.15E-09	78995.3	77970.3	77776.7	74954.2	77807.4	77249.8	27476.2	28746.9	25744.1
155	3	473.538	1417.593	4.49	HYDGSYSTFGER	sp|P01023	0.010671	119347.3	110276.4	108682.1	105590.4	106192.1	105183.6	108330.8	107231.1	113024.0
156	5	651.924	3254.583	12.49	YDVENCLANKVDLSFSPS	sp|P01023	0.000329	388374.7	444256.3	396036.4	367389.4	527199.0	367425.2	208543.8	218255.6	180744.3
161	3	616.966	1847.876	14.11	QFSFPLSSEPFQGSYK	sp|P01023	0.005881	192938.9	227450.6	201626.6	180969.0	140890.8	164986.7	144752.9	149454.2	138751.7
179	3	374.217	1119.630	7.80	SIYKPGQTVK	sp|P01023	0.000332	70468.0	67584.4	77196.2	83130.3	64716.4	78309.5	46723.5	40364.3	41008.9
180	3	704.030	2109.070	13.88	LHTEAQIQEEGTVVELTG	sp|P01023	2.48E-05	278854.8	402243.7	302608.4	300311.1	329161.1	333809.2	141494.0	136071.7	152335.5
190	3	295.192	882.556	6.28	DLKPAIVK	sp|P01023	2.50E-06	44710.0	40551.5	43606.6	42591.8	43157.8	39535.9	23202.3	24999.0	26326.6
215	4	387.207	1544.797	10.96	LVHVEEPHTETVR	sp|P01023	0.004499	79077.9	100526.5	89276.9	80806.7	85625.1	86264.0	64508.3	72587.5	66274.5
224	5	668.504	3337.484	5.54	SPCYGYQWVSEEHEEAH	sp|P01023	4.16E-06	271734.2	338801.3	265140.1	275312.4	266925.3	253461.0	128711.2	115846.7	109752.0
228	3	594.009	1779.006	15.90	DTVIKPLLVEPEGLEK	sp|P01023	1.08E-05	155478.2	142172.7	154043.4	160210.3	207434.5	147855.3	54849.0	56517.2	45092.9
235	2	709.803	1417.592	14.12	HYDGSYSTFGER	sp|P01023	0.049291	152122.7	161316.0	130470.5	142284.2	157389.4	142758.9	127211.7	119369.3	133189.1
237	4	559.226	2232.874	5.61	KYSDASDCHGEDSQAFC	sp|P01023	0.000213	157808.1	163778.2	147299.7	141761.8	150509.1	150606.1	114002.3	119992.8	104741.4
238	2	317.700	633.386	1.34	VFQLK	sp|P01023	8.40E-06	38205.8	38675.1	39678.8	35986.3	42457.7	33734.7	20461.9	20537.4	20707.5
243	2	445.235	888.456	5.84	SLNEEAVK	sp|P01023	0.020608	53764.4	42966.0	49572.8	44724.9	39447.3	44802.9	51014.9	42928.0	45714.9
244	2	383.211	764.408	3.92	GEAFTLK	sp|P01023	1.07E-06	50895.0	52576.0	50570.3	48750.5	67907.2	52609.1	13075.6	13231.0	16307.9
247	3	539.955	1616.844	11.11	TEVSSNHVLIYLDK	sp|P01023	1.94E-06	134068.2	156524.0	131726.6	128332.5	156185.4	117696.1	34440.2	36801.9	42784.0
251	3	362.210	1083.609	6.82	GHFSISIPVK	sp|P01023	5.75E-06	59677.4	75216.5	64767.2	64175.1	73394.5	55437.4	20285.0	20638.8	25220.2
253	2	403.706	805.398	4.84	GPTQEFK	sp|P01023	9.72E-05	68621.1	61640.9	75806.4	70455.5	60375.3	68107.8	38170.0	43244.8	41935.4
261	3	309.188	924.541	8.96	TGTHGLLVK	sp|P01023	2.91E-06	40893.9	39784.7	44584.0	47930.5	40624.1	46608.1	16060.8	15338.6	16129.2
262	2	923.023	1844.031	15.90	LLIYAVLPTGDVIGDSAK	sp|P01023	6.23E-06	293530.3	242787.6	291668.1	323471.2	225997.9	269729.9	73150.0	88257.8	72898.4
284	3	615.685	1844.033	18.11	LLIYAVLPTGDVIGDSAK	sp|P01023	4.71E-05	150249.5	116923.7	141855.1	170745.3	110531.9	137579.8	48032.4	53088.4	47014.9
287	3	550.618	1648.833	7.48	SSSNEEVMFLTVQVK	sp|P01023	0.018151	100613.4	140024.6	104025.5	80392.8	109521.1	73969.2	55759.4	55782.9	78531.8
290	3	522.617	1564.829	12.16	ALLAYAFALAGNQDK	sp|P01023	4.39E-06	85941.2	69157.8	89014.6	90725.8	77303.9	86927.4	34656.9	34081.5	29641.5
300	4	513.019	2048.046	13.00	VDLSFSPSQSLPASHAHL	sp|P01023	4.76E-06	122766.5	153864.2	122573.3	129315.6	128636.9	119091.9	40035.2	40109.6	53020.3
318	3	745.298	2232.872	16.96	KYSDASDCHGEDSQAFC	sp|P01023	1.50E-05	160125.0	163321.0	148169.8	143345.9	155061.4	155227.0	103120.0	107611.1	94040.3
319	3	362.865	1085.573	3.59	SASNMAIVDVK	sp|P01023	9.98E-08	47912.0	44884.9	45141.2	47655.3	43462.7	48251.3	17821.4	19528.0	20132.1
322	5	1070.761	5348.769	14.18	DTVIKPLLVEPEGLEKETT	sp|P01023	1.19E-06	679370.6	676385.1	653905.4	568909.9	497730.8	589035.2	########	1151941.2	########

323	3	796.763	2387.268	17.90	AYIFIDEAHITQALIWLSQI	sp|P01023	0.00015	207374.2	179530.9	211225.1	242172.2	162338.4	194612.9	85216.3	94182.0	82921.2
325	2	613.280	1224.546	8.00	YDVENCLANK	sp|P01023	1.40E-08	80711.2	76807.0	73131.3	96866.7	89030.9	81806.2	15133.5	12607.3	16274.5
345	2	783.421	1564.828	15.90	ALLAYAFALAGNQDK	sp|P01023	2.95E-05	119931.0	87980.6	121567.9	131539.7	111313.8	134100.7	48487.6	45384.9	37761.1
363	4	1227.616	4906.435	20.84	APVGHFYEPQAPSAEVEI	sp|P01023	0.015555	418969.9	351414.4	409639.6	480815.7	430644.5	454060.0	476717.2	410961.5	461237.1
367	4	820.414	3277.627	15.44	VSVQLEASPAFLAVPVEK	sp|P01023	0.000825	200739.5	175858.7	233493.1	225222.7	239486.2	225989.2	157071.9	126341.0	125397.6
370	3	702.599	2104.774	18.95	YSDASDCHGEDSQAFCE	sp|P01023	0.007323	149035.8	121276.9	117356.9	111424.8	105876.9	106266.3	100860.1	90773.8	79547.6
391	3	471.872	1412.595	8.67	EQAPHCICANGR	sp|P01023	6.65E-08	82201.8	69484.2	77640.4	91168.5	85513.7	86092.9	27587.2	27208.5	24131.4
422	2	630.291	1258.567	14.06	VGFYESDVMGR	sp|P01023	0.036243	43964.5	50226.5	48628.0	52421.5	66632.7	58166.7	62310.6	59618.3	78885.6
423	3	666.371	1996.091	17.11	LLLQQVSLPELPGEYSMK	sp|P01023	4.24E-07	133727.1	136373.6	116570.7	132083.5	108047.9	123482.4	27665.5	29581.7	20866.2
429	4	835.378	3337.481	15.83	SPCYGYQWVSEEHEEAH	sp|P01023	2.06E-05	181954.8	254124.5	165117.7	172731.0	199649.2	168594.3	66740.5	61281.1	54099.5
461	4	1040.292	4157.140	15.25	DTVIKPLLVEPEGLEKETT	sp|P01023	7.56E-06	325377.9	223653.9	319073.8	365479.4	253640.6	308064.3	66405.4	80227.3	68050.3
462	2	410.712	819.410	6.31	QSSEITR	sp|P01023	0.039906	91558.3	74806.5	85770.1	104479.7	72417.3	88125.2	66370.3	62066.8	82856.4
483	4	383.180	1528.693	6.55	TAQEGDHGSHVYTK	sp|P01023	0.014693	97799.4	84107.5	97694.1	109002.9	93452.9	108784.9	53586.0	74466.5	52756.0
492	3	628.688	1883.043	18.11	VSVQLEASPAFLAVPVEK	sp|P01023	7.21E-09	88513.3	71887.2	72298.2	101627.0	82327.9	98149.5	9091.8	8740.5	7763.7
505	3	1055.195	3162.562	18.52	YNILPEKEEFPFALGVQTL	sp|P01023	7.27E-10	312793.6	257491.2	289170.0	385925.6	370176.9	356648.7	36891.8	33663.3	31137.6
543	2	605.826	1209.638	10.83	LPPNVVEESAR	sp|P01023	3.68E-07	207155.7	255478.3	208627.7	225142.3	291557.7	226527.7	58485.9	55926.7	61145.8
548	4	791.648	3162.563	15.73	YNILPEKEEFPFALGVQTL	sp|P01023	4.72E-10	188389.3	159007.3	180648.5	223799.3	206965.0	201961.3	22210.4	22310.0	19873.1
624	3	471.544	1411.611	9.92	EQAPHCICANGR	sp|P01023	1.79E-06	35579.0	33527.6	38378.7	32483.0	32566.6	33896.4	14898.7	13587.9	11462.6
676	3	451.620	1351.837	10.26	GVPIPNKVIFIR	sp|P01023	5.42E-08	8788.7	10644.9	9168.0	7848.4	8782.8	7058.8	60664.0	59529.0	77284.8
693	3	937.488	2809.442	16.17	VVSMDENFHPLNELIPLV	sp|P01023	0.000417	114023.1	123242.8	126594.2	173376.2	120800.8	169338.8	71513.3	75405.7	76055.6
700	3	419.220	1254.637	6.08	AIGYLNTGYQR	sp|P01023	0.005949	18336.7	17930.6	19000.4	18260.5	16847.9	18521.1	23594.0	20745.5	25631.7
723	6	847.775	5080.606	10.99	LLIYAVLPTGDVIGDSAKY	sp|P01023	5.75E-08	91620.4	89044.0	96164.6	65211.1	63660.6	72510.7	221191.3	251672.6	248983.6
734	2	836.934	1671.853	13.12	TEHPFTVEEFVLPK	sp|P01023	2.47E-05	73620.1	59814.9	70423.2	73630.4	86695.9	74106.1	35385.8	27864.1	26772.5
736	4	820.168	3276.642	16.44	VSVQLEASPAFLAVPVEK	sp|P01023	0.005301	100428.5	130615.9	95452.0	77085.9	94112.1	88652.2	65059.4	70524.2	66244.6
759	2	777.896	1553.777	18.11	VTGEGCVYLQTSLK	sp|P01023	1.64E-09	135836.7	167032.0	140819.4	166533.9	172999.1	179984.2	28345.6	26275.5	28968.2
767	4	474.270	1893.051	8.37	GHFSISIPVKSDIAPVAR	sp|P01023	0.000806	21933.8	35227.5	23398.6	19121.2	23513.7	17550.0	42783.0	49399.1	63625.3
777	2	942.528	1883.042	17.95	VSVQLEASPAFLAVPVEK	sp|P01023	2.34E-09	89716.5	76849.6	72320.0	109517.9	95681.9	115231.2	8815.5	8302.4	7080.7
780	5	982.294	4906.435	16.55	APVGHFYEPQAPSAEVEI	sp|P01023	0.019983	151482.1	125542.0	144976.0	167083.7	142438.4	153681.1	171649.6	150048.2	166632.2
843	2	567.796	1133.578	11.72	SASNMAIVDVK	sp|P01023	0.000106	21449.8	21398.0	18212.7	25414.4	36872.3	23794.7	7186.9	6587.6	10021.1
888	3	574.651	1720.930	11.25	ALLAYAFALAGNQDKR	sp|P01023	2.26E-05	46032.8	33587.6	32338.7	40795.3	29521.7	36408.8	11954.9	11267.3	10104.6
899	3	790.728	2369.164	12.16	QQNAQGGFSSTQDTVVA	sp|P01023	2.39E-07	65925.7	60474.5	60178.4	67827.5	63450.2	66957.0	25734.3	28995.5	28201.8
966	4	814.653	3254.582	16.49	YDVENCLANKVDLSFSPS	sp|P01023	0.001846	86740.0	118267.7	89953.7	71070.6	147756.9	80549.8	38763.5	39444.5	27856.4
977	2	560.822	1119.630	11.28	SIYKPGQTVK	sp|P01023	2.49E-05	22572.2	22302.6	24230.6	26988.0	26235.5	27175.2	12230.8	10123.3	11224.4
980	3	799.722	2396.145	19.38	ETTFNSLLCPSGGEVSEE	sp|P01023	7.13E-08	77287.5	68578.7	73527.2	104091.8	84323.9	104931.6	21565.2	18450.9	17758.2

1006	4	619.562	2474.220	17.38	SLFTDLEAENDVLHCVAF	sp|P01023	0.049453	45813.7	35253.3	39613.3	38731.8	26555.8	33015.3	29205.3	30282.0	27336.5
1041	3	691.982	2072.925	14.95	MCPQLQQYEMHGPEGLI	sp|P01023	0.003909	35969.0	43534.8	43598.9	49014.5	54387.4	52297.7	52451.1	53395.2	54635.3
1069	3	634.294	1899.861	14.90	FSGQLNSHGCFYQQVK	sp|P01023	0.004493	43301.0	37808.6	36898.7	39434.9	51164.4	37253.3	24235.4	20734.7	29070.6
1104	2	624.840	1247.665	12.79	LSFYYLIMAK	sp|P01023	4.46E-08	22724.5	22102.3	26096.2	31527.6	27794.1	32572.4	7234.1	7991.1	8151.2
1117	3	446.889	1337.645	5.75	NALFCLESAWK	sp|P01023	9.96E-06	11882.3	13066.5	14728.0	12314.6	10579.8	11397.7	5618.7	6152.5	5276.8
1125	4	820.414	3277.627	15.44	VSVQLEASPAFLAVPVEK	sp|P01023	0.000467	52570.3	54814.3	54357.4	48845.2	45912.7	54583.9	38337.3	41096.5	33683.8
1197	3	515.939	1544.797	13.17	LVHVEEPHTETVR	sp|P01023	0.004916	20549.5	30073.4	22975.0	20798.0	26279.0	22790.4	14757.3	16666.4	15241.4
1205	3	465.565	1393.674	8.02	NEDSLVFVQTDK	sp|P01023	5.79E-05	19369.6	20733.5	20005.4	16882.4	18551.1	17369.2	9006.9	8460.0	6188.9
1214	2	566.302	1130.589	7.28	QGIPFFGQVR	sp|P01023	4.67E-07	21870.2	24304.6	21640.4	23543.2	21126.2	25749.3	10006.2	10318.4	9437.2
1252	4	508.265	2029.031	4.36	SGGRTEHPFTVEEFVLPK	sp|P01023	1.29E-06	7866.0	10068.0	8414.4	5028.0	6868.7	5473.3	44357.8	45260.7	72199.9
1257	3	510.571	1528.692	8.37	TAQEGDHGSHVYTK	sp|P01023	0.012117	54392.7	44428.7	52795.5	60031.9	52137.6	58850.0	17590.6	38414.1	19052.0
1305	4	597.824	2387.269	12.89	AYIFIDEAHITQALIWLSQI	sp|P01023	8.12E-05	32458.4	26265.4	34711.1	39151.1	26006.8	31303.5	12469.0	12793.0	12386.8
1307	2	849.425	1696.836	15.90	SSSNEEVMFLTVQVK	sp|P01023	2.64E-06	40272.0	40711.8	47150.8	57818.6	54381.0	60960.8	28257.9	28242.9	25044.4
1343	3	682.373	2044.096	17.11	LLLQQVSLPELPGEYSMK	sp|P01023	7.15E-08	31799.5	27789.0	30507.6	51837.1	44895.0	55081.7	10577.5	10450.8	8735.8
1350	2	504.715	1007.415	3.65	QKDNGCFR	sp|P01023	0.001025	13126.8	14038.6	14177.6	16493.7	15105.6	15230.5	11477.6	11368.1	9483.5
1428	3	349.535	1045.583	5.04	FEVQVTVPK	sp|P01023	3.62E-06	8376.9	8478.9	9151.1	7544.2	8033.3	7920.7	4365.9	4275.7	5004.5
1454	4	597.556	2386.193	12.28	QQNAQGGFSSTQDTVVA	sp|P01023	0.005939	24328.2	24587.5	26646.3	24757.4	24602.1	21501.2	16236.2	17255.6	19545.0
1478	2	916.432	1830.849	19.90	QFSFPLSSEPFQGSYK	sp|P01023	0.000395	50684.7	42979.6	47112.3	54410.7	48442.0	51632.1	25035.5	29922.3	18820.5
1505	2	809.428	1616.842	14.17	TEVSSNHVLIYLDK	sp|P01023	5.03E-05	38964.6	53112.1	34934.2	32079.7	54892.4	31948.2	8099.4	8897.8	11964.1
1521	4	703.368	2809.442	11.29	VVSMDENFHPLNELIPLV	sp|P01023	2.67E-05	31306.7	31822.4	34455.5	45669.4	37031.2	48232.3	19974.1	20861.2	20057.5
1524	4	814.411	3253.615	19.44	QQNAQGGFSSTQDTVVA	sp|P01023	4.18E-05	40948.0	47159.4	37080.8	25114.2	20126.0	21698.6	48228.9	48239.1	42102.0
1526	2	950.445	1898.876	12.95	FSGQLNSHGCFYQQVK	sp|P01023	0.000337	33394.6	50273.6	30002.3	47543.1	55439.6	45201.3	16789.8	15539.0	20625.0
1555	4	691.366	2761.436	8.12	VVSMDENFHPLNELIPLV	sp|P01023	0.000315	36728.4	35914.3	44950.0	39347.0	31140.3	36423.0	17465.9	20886.0	14115.8
1564	3	721.733	2162.178	13.10	VSNQTLSLFFTVLQDVPV	sp|P01023	1.06E-06	31602.4	38563.1	32474.4	35898.5	34231.3	37814.6	9492.6	10058.5	7233.2
1572	4	779.160	3112.613	11.44	FRVVSMDENFHPLNELIP	sp|P01023	4.31E-05	29857.5	21956.0	27535.0	30588.3	24586.1	31346.3	63606.3	59446.4	53655.4
1589	3	342.488	1024.441	4.15	QKDNGCFR	sp|P01023	0.014574	18558.5	14435.7	16122.7	19964.8	15250.1	17529.6	16067.4	17377.6	18567.1
1649	3	451.551	1351.632	6.38	DMYSFLEDMGLK	sp|P01023	7.06E-07	12373.8	14362.5	12083.4	13409.8	15731.1	14199.2	4091.1	3287.7	4398.8
1668	3	400.894	1199.661	5.27	LSFYYLIMAK	sp|P01023	2.60E-06	13016.8	16005.6	12766.7	10832.9	14328.6	11992.2	3537.5	4052.5	3469.1
1721	3	566.620	1696.839	9.71	SSSNEEVMFLTVQVK	sp|P01023	2.86E-05	15581.9	14942.2	18186.2	22901.5	19725.0	22676.3	12140.2	12150.4	12195.9
1783	3	443.593	1327.756	6.00	KLSFYYLIMAK	sp|P01023	2.76E-05	12966.3	21263.3	13607.7	12145.7	16368.6	12634.7	3494.4	3157.4	4115.6
1793	2	463.277	924.540	9.50	TGTHGLLVK	sp|P01023	3.30E-06	8872.4	9185.5	10059.9	11095.7	10998.1	11325.2	2615.1	2485.9	2751.2
1795	4	675.864	2699.426	14.91	TEHPFTVEEFVLPKFEVQ	sp|P01023	4.34E-08	7993.2	7237.7	8200.2	4467.8	3719.3	3587.5	49564.2	64167.9	40021.2
1828	2	402.199	802.384	4.61	QSSEITR	sp|P01023	6.04E-06	5484.9	5549.1	5405.9	6787.3	6247.7	6946.6	3519.6	3184.8	2748.0
1833	4	419.230	1672.891	3.47	LVHVEEPHTETVRK	sp|P01023	0.023599	13018.3	11144.5	12861.3	10931.1	13592.1	10701.6	10262.0	9585.0	9804.4

1867	3	459.594	1375.760	3.31	KLSFYYLIMAK	sp|P01023	5.27E-06	11344.4	7356.2	10090.7	14099.2	10699.5	13007.7	2312.7	2741.1	2845.1
1904	3	764.335	2289.984	18.11	DLTGFPGPLNDQDNEDC	sp|P01023	1.79E-06	31043.5	36830.1	31014.1	36684.0	29100.8	34530.4	12007.5	12699.7	10703.4
1949	2	543.794	1085.573	7.77	SASNMAIVDVK	sp|P01023	5.41E-09	12981.7	12603.8	11975.9	12882.2	12560.2	13107.1	4114.3	3977.1	4039.9
1988	2	765.354	1528.693	14.91	TAQEGDHGSHVYTK	sp|P01023	0.014772	49354.1	43519.6	48066.9	59985.6	52601.7	66098.1	34222.1	28470.6	28169.9
1996	6	818.747	4906.436	15.42	APVGHFYEPQAPSAEVEI	sp|P01023	0.030029	43475.6	35799.9	41393.8	47073.8	39926.4	44659.6	48082.7	41763.9	44813.7
2021	2	504.223	1006.431	3.36	QKDNGCFR	sp|P01023	0.010389	8032.8	7254.6	8435.1	8608.9	7719.6	8339.0	6402.7	6775.0	5151.0
2083	4	897.950	3587.771	19.05	ETTFNSLLCPSGGEVSEE	sp|P01023	3.94E-06	106152.9	85165.2	94846.0	107936.0	86672.9	99577.4	34796.1	33732.8	28487.1
2099	3	713.025	2136.052	12.95	HNVYINGITYTPVSSTNEK	sp|P01023	5.34E-05	25099.9	27608.4	22027.6	25376.1	29926.3	23069.8	8483.6	6620.1	10818.3
2114	5	832.435	4157.141	17.46	DTVIKPLLVEPEGLEKETT	sp|P01023	6.57E-06	48048.2	30150.4	43946.1	49560.8	36040.9	46248.6	8532.0	9756.8	8936.4
2196	2	890.509	1779.004	16.01	DTVIKPLLVEPEGLEK	sp|P01023	4.65E-05	29719.7	25739.7	25323.0	26403.3	49630.8	29514.0	7284.8	7823.3	5572.3
2233	4	763.650	3050.571	18.29	LLIYAVLPTGDVIGDSAKY	sp|P01023	7.50E-05	31415.8	26204.3	27939.4	29060.9	23035.9	29994.9	14347.9	12655.6	10871.4
2236	4	528.276	2109.073	8.11	LHTEAQIQEEGTVVELTG	sp|P01023	0.000134	12137.4	16098.1	14187.0	12768.4	12834.9	14374.3	8709.8	8810.0	8698.3
2258	1	803.390	802.383	1.46	QSSEITR	sp|P01023	0.000147	12119.9	16268.4	11071.7	12622.8	17159.8	14157.4	5948.4	6132.0	5455.7
2309	5	1038.706	5188.493	16.02	AVDQSVLLMKPDAELSAS	sp|P01023	1.47E-06	57823.8	45665.1	52746.8	83372.8	103092.6	85170.9	19386.9	16695.0	13201.0
2334	5	858.054	4285.232	6.31	KDTVIKPLLVEPEGLEKET	sp|P01023	2.93E-07	42345.6	39302.2	31730.6	43830.4	36904.5	39397.1	8468.7	9510.2	7546.6
2346	3	1196.931	3587.770	17.83	ETTFNSLLCPSGGEVSEE	sp|P01023	1.08E-06	146680.6	121053.7	139007.3	148975.4	128909.2	141184.2	43928.0	44113.3	34185.9
2381	6	892.469	5348.768	13.39	DTVIKPLLVEPEGLEKETT	sp|P01023	7.57E-07	64660.6	63169.7	60534.6	54472.6	46480.5	52913.2	105472.7	109034.5	100507.6
2534	5	613.929	3064.609	4.54	FRVVSMDENFHPLNELIP	sp|P01023	0.000188	18703.8	14872.8	15998.2	12968.0	11299.4	14207.2	24869.7	23953.5	24251.5
2622	1	1099.579	1098.572	5.57	QTVSWAVTPK	sp|P01023	0.014245	12277.3	18354.3	15923.6	15991.2	27121.0	18072.1	15046.9	13776.9	13478.8
2745	4	624.831	2495.296	4.25	NEDSLVFVQTDKSIYKPG	sp|P01023	7.68E-08	7769.1	9958.8	8957.9	4538.3	5811.3	4982.8	29907.7	30742.9	32121.3
2755	3	383.546	1147.616	6.71	QGIPFFGQVR	sp|P01023	0.001314	5913.4	5004.6	5702.8	5662.7	4332.6	5115.7	3514.7	3212.6	2937.3
2907	3	632.024	1893.050	14.70	GHFSISIPVKSDIAPVAR	sp|P01023	0.004079	7273.7	13468.4	6588.4	5342.4	8092.4	5580.8	14197.6	16637.4	21897.9
2949	2	1238.116	2474.217	15.22	SLFTDLEAENDVLHCVAF	sp|P01023	0.005765	44764.5	32375.8	31741.5	40642.4	24569.3	32682.7	17560.9	20336.2	18455.3
3088	6	543.438	3254.585	8.57	YDVENCLANKVDLSFSPS	sp|P01023	3.56E-05	12803.0	14961.6	12824.4	11699.0	16678.6	11860.4	5554.3	6068.4	5372.0
3154	2	707.305	1412.595	14.12	EQAPHCICANGR	sp|P01023	7.37E-09	15003.8	14213.8	13760.0	18051.2	22069.8	17270.4	3344.7	3262.0	3231.9
3387	3	441.240	1320.699	7.75	PVPGHVTVSICR	sp|P01023	7.06E-08	6563.6	8212.2	6923.8	7908.8	9857.2	8174.1	1195.1	1127.4	1321.9
3405	2	339.697	677.379	3.22	TTVMVK	sp|P01023	0.010167	5945.8	5046.6	5968.6	7988.3	6487.3	8419.1	3822.0	4016.5	4348.4
3448	4	377.958	1507.802	2.30	TGTHGLLVKQEDMK	sp|P01023	0.000176	11461.7	9092.4	11381.6	8801.1	7153.4	9226.2	20374.3	19721.3	22904.7
3629	4	1298.131	5188.494	19.81	AVDQSVLLMKPDAELSAS	sp|P01023	1.29E-05	37632.0	27797.3	32337.5	57748.8	78032.2	54583.2	12577.9	8934.6	7248.1
3661	4	963.002	3847.977	16.30	KYFPETWIWDLVVVNSAG	sp|P01023	0.000314	59046.0	54805.5	66971.1	56845.3	54584.4	58682.8	37044.8	40168.2	35724.8
3691	5	1017.128	5080.603	5.72	LLIYAVLPTGDVIGDSAKY	sp|P01023	7.94E-05	16412.4	20938.5	14706.3	8927.5	15196.3	14089.0	45411.7	52035.7	43414.5
3880	2	513.228	1024.441	1.38	QKDNGCFR	sp|P01023	0.046617	11198.7	8007.4	7543.2	12477.2	9181.7	10020.2	9245.4	8335.0	12021.2
3959	4	1338.199	5348.766	16.23	DTVIKPLLVEPEGLEKETT	sp|P01023	2.42E-05	45984.3	50225.5	47327.8	44471.7	38134.3	46629.2	81985.3	79319.5	74923.0
4244	4	859.433	3433.704	12.70	IAQWQSFQLEGGLKQFSI	sp|P01023	5.95E-07	9100.9	8178.4	6482.1	5810.8	5021.3	5427.6	22810.1	26739.6	25117.8

4365	4	892.673	3566.664	18.44	HNVYINGITYTPVSSTNEK	sp|P01023	2.04E-06	13780.5	10068.4	13146.3	25857.3	21486.7	31682.1	4702.2	4147.5	3909.7
5294	5	612.328	3056.603	7.02	SGGRTEHPFTVEEFVLPK	sp|P01023	7.72E-11	781.9	624.2	646.5	229.0	241.7	188.3	20146.9	20400.5	19478.6
5516	6	579.451	3470.659	1.32	HNVYINGITYTPVSSTNEK	sp|P01023	1.89E-06	6946.9	9466.8	7156.4	7212.1	8713.1	8023.9	1962.6	1697.3	2027.5
6297	3	1636.484	4906.431	11.76	APVGHFYEPQAPSAEVEI	sp|P01023	0.015981	24699.9	21709.3	26873.9	31530.8	25106.7	30270.6	28538.7	27323.8	26718.3
6507	6	896.801	5374.761	5.73	LVHVEEPHTETVRKYFPE	sp|P01023	1.90E-08	9786.1	9832.3	9414.3	5192.2	4144.7	5515.8	32046.6	34747.1	33891.2
6798	6	771.933	4625.553	14.09	TEVSSNHVLIYLDKVSNQ	sp|P01023	5.90E-09	8870.3	8166.0	8394.8	5105.9	4983.2	5143.7	17879.6	20232.6	20233.8
7293	3	611.291	1830.850	15.11	QFSFPLSSEPFQGSYK	sp|P01023	0.004802	3525.3	3025.4	4242.5	4174.2	3439.1	3588.6	1887.9	2657.0	1697.8
7574	4	1073.347	4289.358	20.97	SSGSLLNNAIKGGVEDEV	sp|P01023	5.39E-09	7440.2	7748.3	8445.4	4507.3	3592.2	3891.7	37783.5	38319.0	33811.6
7606	2	724.827	1447.639	16.06	DMYSFLEDMGLK	sp|P01023	2.65E-05	2308.5	1617.8	2195.2	4577.3	3796.2	5067.4	1361.4	1287.3	1123.8
7954	3	885.498	2653.473	12.57	SDIAPVARLLIYAVLPTGD	sp|P01023	5.66E-08	8791.3	8326.5	9585.0	7102.9	7333.5	7540.6	1915.0	1395.2	1640.5
8299	3	931.157	2790.451	11.90	AFQPFFVELTMPYSVIRG	sp|P01023	1.75E-06	3208.0	4283.6	3503.1	4006.0	2955.1	3377.3	10390.2	11221.5	10693.5
8405	3	1145.574	3433.701	21.44	IAQWQSFQLEGGLKQFSI	sp|P01023	1.24E-05	5865.3	5807.2	3860.5	4202.7	3390.3	3268.1	15789.8	17626.9	15733.5
9134	3	1283.666	3847.975	14.09	KYFPETWIWDLVVVNSA(	sp|P01023	8.00E-05	24307.5	22156.9	26931.0	24699.5	23336.2	25042.4	13977.2	16248.4	13637.4
9781	4	541.552	2162.178	12.44	VSNQTLSLFFTVLQDVPV	sp|P01023	2.71E-07	2017.0	2431.8	2148.7	2363.2	2328.0	2426.2	612.8	553.5	468.4
9979	4	757.688	3026.722	14.52	VSNQTLSLFFTVLQDVPV	sp|P01023	2.34E-08	1680.2	1675.0	1890.7	986.2	845.1	969.0	5121.8	5284.7	5452.9
10891	2	1082.095	2162.176	17.90	VSNQTLSLFFTVLQDVPV	sp|P01023	2.23E-05	4603.5	6091.1	5108.4	5459.7	5300.6	6157.5	1080.0	1480.1	739.7
11196	6	1301.493	7802.913	9.58	APVGHFYEPQAPSAEVEI	sp|P01023	1.44E-06	8449.5	12359.8	9694.1	4519.1	4858.5	5566.8	37346.8	50196.1	39277.4
11931	4	941.260	3761.009	7.07	TEVSSNHVLIYLDKVSNQ	sp|P01023	0.006296	5762.6	7424.9	4659.7	3837.2	3085.2	2841.9	3805.8	4168.5	4035.7
12835	5	770.603	3847.978	14.58	KYFPETWIWDLVVVNSA(	sp|P01023	0.001699	6752.2	6351.0	8054.5	6360.4	5839.7	6571.8	4191.0	4551.1	3856.7
13692	5	1087.955	5434.737	15.26	APVGHFYEPQAPSAEVEI	sp|P01023	1.20E-08	2545.6	2461.5	3065.2	1352.5	1153.6	1312.6	18728.6	17650.3	17854.0
14561	5	858.879	4289.360	16.18	SSGSLLNNAIKGGVEDEV	sp|P01023	1.77E-08	1862.6	1772.0	2036.4	929.5	724.6	917.2	9748.5	9968.3	9283.9
14593	5	926.118	4625.552	16.15	TEVSSNHVLIYLDKVSNQ	sp|P01023	3.78E-07	3648.7	3767.1	3465.7	1967.4	2260.4	2082.7	8529.5	8471.4	7515.5
17370	4	948.514	3790.025	6.80	AFQPFFVELTMPYSVIRG	sp|P01023	0.00304	3652.0	3622.1	4040.8	2945.0	2292.6	2449.0	3536.3	2896.5	3492.3
19873	4	1359.692	5434.739	6.78	APVGHFYEPQAPSAEVEI	sp|P01023	1.86E-09	1559.6	1852.6	1516.9	983.7	857.4	816.5	13710.4	14256.0	14052.5
24252	6	715.901	4289.361	15.45	SSGSLLNNAIKGGVEDEV	sp|P01023	3.46E-09	617.9	546.8	517.0	237.0	223.5	266.4	3502.6	3322.4	3013.8
24315	5	753.209	3761.011	7.73	TEVSSNHVLIYLDKVSNQ	sp|P01023	0.004577	1312.5	1555.8	1047.6	875.4	672.3	606.5	720.9	856.2	795.4
30917	6	627.842	3761.011	6.90	TEVSSNHVLIYLDKVSNQ	sp|P01023	0.007649	543.5	689.7	430.1	311.1	346.5	272.9	367.2	475.1	395.6
35516	3	722.061	2163.162	8.32	VSNQTLSLFFTVLQDVPV	sp|P01023	8.76E-05	254.5	240.5	203.2	403.3	273.8	285.4	75.1	66.7	34.8
273	3	733.383	2197.126	12.43	VPVAVQGEDTVQSLTQG	sp|P01024	0.001397	149569.1	118739.5	145572.6	181907.8	249680.3	208037.7	105018.5	123630.5	91657.4
283	2	645.308	1288.602	16.11	SGSDEVQVGQQR	sp|P01024	1.03E-06	72620.6	65407.2	67809.3	106258.6	112654.0	101894.6	69766.7	67120.4	70495.4
313	2	444.746	887.477	4.00	IWDVVEK	sp|P01024	1.03E-06	33964.1	34706.1	35136.7	41840.4	47709.8	38426.5	38615.7	36017.4	42319.1
347	3	596.662	1786.965	11.52	SGIPIVTSPYQIHFTK	sp|P01024	0.020642	75869.2	69177.4	88801.4	94294.0	107358.0	94085.3	107469.9	107984.8	101182.2
372	3	397.867	1190.579	7.46	SDDKVTLEER	sp|P01024	0.000303	28720.4	24097.3	25457.5	37664.7	40015.1	34680.5	26940.6	24438.2	28349.5
412	2	403.245	804.476	7.59	TFISPIK	sp|P01024	0.037616	20685.0	23661.0	21161.2	23012.2	25708.7	25604.0	24629.0	24997.8	26081.3

497	3	374.512	1120.514	3.65	LCRDELCR	sp|P01024	0.000484	14144.7	12217.4	15782.5	17897.5	14688.6	18040.9	26527.4	25335.4	24353.0
552	2	701.422	1400.830	14.06	SSLSVPYVIVPLK	sp|P01024	7.72E-05	57675.3	61182.6	53120.5	80068.8	57117.5	69824.9	31378.8	31446.9	33033.6
601	3	606.304	1815.889	15.11	SNLDEDIIAEENIVSR	sp|P01024	0.012798	38990.9	35633.1	41787.5	40819.6	41483.9	43522.8	47257.5	45035.7	43789.6
615	3	620.304	1857.889	4.20	VFLDCCNYITELRR	sp|P01024	0.026115	43249.3	43235.4	43721.2	46037.6	59645.0	51427.6	76809.6	67346.8	50539.8
652	2	650.798	1299.581	11.10	ACEPGVDYVYK	sp|P01024	0.000147	42247.7	43371.4	35215.1	46713.8	60447.7	51578.0	24822.1	23325.7	27983.5
667	3	624.684	1871.030	11.95	TELRPGETLNVNFLLR	sp|P01024	0.000137	45168.7	40664.6	50530.9	61230.4	51202.3	59079.3	26535.8	26204.6	27763.1
714	2	421.772	841.529	6.40	VVLVAVDK	sp|P01024	0.003411	14745.4	18433.4	14984.6	17403.8	26601.1	18972.8	11759.5	10844.4	10793.4
779	3	504.613	1510.818	14.27	LVAYYTLIGASGQR	sp|P01024	0.000108	20492.9	19318.2	20094.4	28536.4	27175.0	26337.8	20815.9	18147.3	17907.5
798	2	402.727	803.440	5.85	SVQLTEK	sp|P01024	0.000306	14475.0	13002.0	14004.0	15800.2	14954.1	17367.1	20376.1	22322.6	22822.6
800	3	384.875	1151.603	6.56	FISLGEACKK	sp|P01024	0.042322	14292.1	17619.2	13682.7	16778.5	18275.3	16422.0	19047.7	18059.5	19030.8
810	2	570.275	1138.535	6.36	FYYIYNEK	sp|P01024	9.18E-08	24006.4	29880.7	25381.4	36368.2	38699.9	38834.6	8322.5	8671.3	9951.2
834	2	365.691	729.367	3.42	TLDPER	sp|P01024	0.040345	15654.4	13425.1	16298.0	18811.6	15129.4	18655.2	21868.1	22794.1	22670.8
836	2	366.201	730.387	5.57	AEDLVGK	sp|P01024	0.000682	12766.3	11463.5	12126.5	15612.3	14237.9	16208.0	11558.8	11969.2	12122.0
848	2	542.283	1082.552	7.63	GYTQQLAFR	sp|P01024	0.000565	17613.8	15017.6	19077.5	25251.7	30643.6	25268.5	13969.7	15559.5	16281.3
948	3	463.905	1388.692	10.07	KQELSEAEQATR	sp|P01024	0.002164	12972.1	12751.4	15406.8	19346.5	18257.5	17851.8	14555.0	14638.3	15696.0
971	2	340.703	679.391	3.36	FLTTAK	sp|P01024	0.03696	8796.1	7236.8	9982.0	9937.8	10066.6	10766.0	10484.3	11049.8	9651.2
1001	2	417.249	832.483	3.89	LPYSVVR	sp|P01024	6.42E-08	11399.1	13394.5	12038.1	16858.9	22493.6	16471.5	2565.4	2410.2	2661.6
1032	2	756.415	1510.816	17.06	LVAYYTLIGASGQR	sp|P01024	0.000162	28636.6	26148.3	27458.9	39897.3	41101.1	37578.0	26737.6	24990.4	21174.6
1074	2	449.743	897.472	2.80	AVLYNYR	sp|P01024	9.85E-07	10331.5	12332.2	10507.0	14845.0	20069.3	16034.1	3529.5	4139.7	4179.4
1096	2	555.818	1109.622	12.12	VLLDGVQNPR	sp|P01024	0.003915	15122.1	12282.8	13494.4	18677.6	21854.9	18372.7	13456.9	13757.4	14505.9
1112	2	385.233	768.451	1.47	VVPEGIR	sp|P01024	1.35E-05	11942.2	11905.8	12589.2	15637.4	16317.6	15788.6	17529.3	16584.6	16486.3
1120	2	596.297	1190.579	5.94	SDDKVTLEER	sp|P01024	2.07E-06	16212.9	14550.8	14361.4	24811.4	25694.4	22608.3	13243.6	12534.2	14086.2
1143	2	444.233	886.452	1.43	NEQVEIR	sp|P01024	1.51E-09	12165.9	11781.5	11964.0	18828.9	16201.2	16467.3	4172.2	4152.8	3957.4
1147	3	542.967	1625.878	14.46	RIPIEDGSGEVVLSR	sp|P01024	0.001912	16178.0	20467.2	17442.0	21941.2	21576.0	22485.9	27747.5	24128.5	28930.6
1153	3	320.855	959.542	2.00	SVQLTEKR	sp|P01024	0.001223	3003.4	2318.7	2869.6	3339.8	3314.4	4106.9	6976.8	7315.7	5751.4
1154	3	413.579	1237.716	6.75	KVLLDGVQNPR	sp|P01024	0.001479	11865.4	12983.1	12975.8	16463.2	15104.3	15853.5	14260.6	13975.6	16465.1
1166	3	552.937	1655.789	6.82	LDKACEPGVDYVYK	sp|P01024	0.001814	16411.0	18472.3	15110.5	18675.3	20795.9	22190.2	20062.3	20318.0	21922.4
1180	3	722.673	2164.998	13.21	AYYENSPQQVFSTEFEV	sp|P01024	1.20E-07	50657.7	43201.1	49015.6	65673.2	55587.6	63762.8	17084.7	14900.2	14782.6
1193	3	320.519	958.536	3.48	EALKLEEK	sp|P01024	8.87E-05	5411.2	4946.3	4912.0	7211.8	6614.9	6687.4	7382.7	7006.7	6731.7
1208	3	815.442	2443.304	12.43	EPGQDLVVLPLSITTDFIP	sp|P01024	2.09E-06	40971.0	37359.1	44938.3	75055.9	60645.5	68288.6	22491.6	20168.8	17781.0
1224	2	512.761	1023.508	9.44	FISLGEACK	sp|P01024	0.006047	9753.7	9074.5	8915.4	12922.0	15253.0	11898.7	9906.6	12461.4	10244.3
1231	2	595.815	1189.616	7.29	DFDFVPPVVR	sp|P01024	6.94E-08	18136.3	17027.8	17852.6	30476.1	19843.6	26861.7	3127.6	2905.7	3084.2
1232	3	614.669	1840.986	8.78	VHQYFNVELIQPGAVK	sp|P01024	4.78E-07	24952.9	34149.4	27921.9	44607.6	49174.8	37614.7	5647.3	4474.4	4316.1
1262	3	619.291	1854.851	15.64	SEETKENEGFTVTAEGK	sp|P01024	1.91E-05	33279.0	38409.0	35731.0	40857.6	31491.2	45961.1	13708.2	15454.2	15380.1

1271	3	404.900	1211.678	11.11	VTIKPAPETEK	sp|P01024	9.44E-09	12087.0	10770.2	12477.2	17200.2	15789.6	17107.0	3739.6	3183.5	3416.1
1278	3	540.285	1617.834	13.99	TKKQELSEAEQATR	sp|P01024	4.27E-07	13374.4	13165.9	13344.9	16438.6	16003.2	14495.3	27726.1	27641.1	30735.7
1279	3	294.864	881.571	3.21	LKGPLLNK	sp|P01024	0.00016	5401.4	6026.6	5529.6	7827.0	8130.7	6879.9	4677.7	4355.4	4902.6
1296	3	314.516	940.525	3.47	EALKLEEK	sp|P01024	6.49E-05	4867.0	4474.7	4521.4	6055.4	5661.7	5924.0	6312.3	6159.3	6201.2
1354	2	314.179	626.344	1.33	FLYGK	sp|P01024	7.76E-07	5258.7	6032.4	5470.1	7789.5	7362.1	7297.8	2632.1	2873.0	2820.0
1375	4	395.959	1579.806	6.13	AKDQLTCNKFDLK	sp|P01024	1.02E-05	13050.5	12647.9	12423.8	16270.3	18270.3	16180.5	8503.2	8567.3	8271.5
1449	2	641.267	1280.519	10.69	CAEENCFIQK	sp|P01024	0.000194	13398.9	13210.8	14623.7	16052.3	16363.9	18020.0	22932.7	25446.4	22306.1
1470	2	939.992	1877.970	16.90	EYVLPSFEVIVEPTEK	sp|P01024	6.89E-08	48897.4	40680.1	46270.7	77838.8	64402.3	77013.2	8025.9	9418.0	6886.3
1514	5	321.768	1603.803	4.33	FYHPEKEDGKLNK	sp|P01024	0.000179	8979.4	8146.0	9367.1	12777.0	10989.6	11113.3	7956.0	7517.4	7540.7
1533	4	368.692	1470.739	1.41	AAVYHHFISDGVR	sp|P01024	0.000311	8811.9	11009.1	8697.8	12763.5	11580.4	12076.7	6934.9	7101.1	7686.0
1628	2	685.870	1369.726	15.06	TIYTPGSTVLYR	sp|P01024	5.27E-06	20064.0	20219.4	20846.7	32091.8	23618.7	36984.8	4658.6	4455.7	6673.6
1636	4	405.466	1617.835	1.30	TKKQELSEAEQATR	sp|P01024	1.87E-06	7374.4	6530.3	7575.2	8886.3	7739.4	8036.9	14077.3	14370.8	15192.1
1664	2	631.305	1260.596	18.06	QELSEAEQATR	sp|P01024	1.84E-05	11523.3	11865.9	11428.0	14457.8	15139.4	14049.9	9795.2	8365.9	9045.6
1681	3	795.794	2384.360	11.97	SSLSVPYVIVPLKTGLQE	sp|P01024	3.50E-05	19021.1	15806.3	19166.7	17258.8	13212.7	16044.2	52148.0	65662.6	38325.6
1701	2	531.748	1061.482	15.11	ADIGCTPGSGK	sp|P01024	1.02E-05	10986.5	9475.3	11881.0	15772.2	15514.8	16426.4	6358.8	6707.0	6389.8
1766	2	349.158	696.302	3.67	FSCQR	sp|P01024	0.004564	13313.5	11869.2	12986.9	18895.0	14026.9	16767.8	19607.4	18155.3	19431.0
1805	3	665.350	1993.030	10.82	KVEGTAFVIFGIQDGEQR	sp|P01024	1.56E-08	32459.9	32404.3	30586.3	44484.5	48325.5	48562.7	16641.8	16227.5	15263.5
1807	3	600.983	1799.927	11.69	VELLHNPAFCSLATTK	sp|P01024	6.80E-06	15837.1	20247.1	19295.3	22483.7	29108.4	24199.8	7898.1	8841.5	8469.4
1848	3	386.873	1157.599	3.24	YISKYELDK	sp|P01024	6.42E-09	7402.8	6450.2	7756.8	11812.2	10909.2	10992.5	1574.9	1913.1	1728.7
1854	2	673.355	1344.695	16.06	EVVADSVWVDVK	sp|P01024	1.69E-08	18757.1	20873.8	19139.2	28731.6	25978.9	26488.4	8055.4	7050.6	7243.1
1858	3	275.497	823.468	4.60	ASHLGLAR	sp|P01024	0.009146	7210.6	5740.8	6855.9	9398.7	7325.9	8761.4	10655.7	10891.4	11280.7
1866	3	469.879	1406.616	9.51	VSHSEDDCLAFK	sp|P01024	2.63E-08	8753.3	12387.3	9309.4	16971.3	15501.7	16491.5	406.3	602.0	660.5
1880	3	716.364	2146.070	12.69	DAPDHQELNLDVSLQLPS	sp|P01024	1.15E-07	30567.9	26105.7	22369.8	39886.8	36278.6	37557.0	6348.4	7585.5	7098.2
1916	2	366.172	730.330	1.33	WEDPGK	sp|P01024	4.82E-09	6090.3	5387.4	5793.7	8694.6	8372.4	8194.1	1670.3	1670.3	1462.2
1919	2	851.901	1701.787	14.11	VFLDCCNYITELR	sp|P01024	0.004415	21260.5	19665.7	19098.6	30166.7	23413.5	21768.3	14830.1	16728.8	12186.3
1952	4	633.333	2529.302	8.08	TMQALPYSTVGNSNNYLI	sp|P01024	0.001196	14584.8	18062.7	14476.0	20610.7	29866.6	17413.5	9682.6	9489.0	6992.8
1968	2	834.373	1666.732	15.06	VYAYYNLEESCTR	sp|P01024	7.30E-05	39497.8	49869.6	37335.1	51226.6	54871.4	48608.8	19419.9	21590.0	25828.4
1987	3	719.701	2156.082	19.11	ILLQGTPVAQMTEDAVDA	sp|P01024	0.00256	21048.8	13871.0	19797.7	34526.5	28168.2	32737.9	18489.3	17269.9	17495.2
1997	3	868.348	2602.023	19.90	DTWVEHWPEEDECQDEI	sp|P01024	2.26E-06	28003.8	30802.3	31605.0	46076.9	51175.5	51169.1	12460.5	14597.8	10885.9
2027	3	717.741	2150.203	18.11	QLYNVEATSYALLALLQLI	sp|P01024	1.86E-07	24963.1	16147.9	25474.5	41827.0	32682.3	40083.8	2606.0	2890.5	2318.0
2122	3	401.546	1201.615	3.68	NTMILEICTR	sp|P01024	5.62E-05	5851.9	7375.7	6288.6	6843.5	8932.4	6993.3	2382.5	2488.7	3062.8
2148	3	943.184	2826.531	14.91	QVREPGQDLVVLPLSITTI	sp|P01024	0.0025	27299.5	17624.0	18069.2	27301.4	20102.4	28976.5	44477.1	41923.6	37872.4
2202	4	733.618	2930.444	11.50	SEFPESWLWNVEDLKEP	sp|P01024	0.000323	129455.7	106206.6	116928.6	160486.1	137314.8	160348.4	96505.4	99571.5	89771.3
2214	3	302.193	903.556	3.36	GVFVLNKK	sp|P01024	0.000328	3074.5	2980.9	3073.0	4393.5	4543.0	4234.3	2532.8	2460.8	2415.2

2216	3	286.833	857.478	1.34	IFTVNHK	sp|P01024	2.06E-06	3695.5	3477.3	4442.9	5626.4	4859.8	5290.3	1560.5	1817.2	1842.6
2300	4	432.776	1727.075	7.30	VVLVAVDKGVFVLNKK	sp|P01024	0.00201	4120.1	6701.0	3801.1	4137.2	6210.2	5266.1	10552.9	10387.3	12566.6
2303	2	480.275	958.535	1.42	EALKLEEK	sp|P01024	0.001367	4828.9	4850.1	4223.8	5989.1	6652.9	5766.2	6586.3	6102.9	6129.8
2360	4	485.248	1936.962	8.57	SGQSEDRQPVPGQQMTI	sp|P01024	0.00381	9491.3	8416.0	9582.3	12213.1	9995.4	10584.5	9450.6	10257.4	9992.4
2403	3	753.042	2256.103	12.78	VQLSNDFDEYIMAIEQTIK	sp|P01024	0.000761	11369.1	9266.2	10784.2	25786.9	18137.2	20499.1	13291.4	12216.8	10924.0
2413	5	676.537	3377.650	12.00	LESEETMVLEAHDAQGD\`	sp|P01024	0.000751	18862.2	20089.4	17543.3	29088.7	27449.6	26385.8	25979.7	24420.6	22467.4
2559	3	662.663	1984.966	12.75	SGQSEDRQPVPGQQMTI	sp|P01024	0.000602	10583.2	13472.8	11131.6	18194.4	21623.8	19070.8	15974.1	15190.2	16282.5
2605	3	832.055	2493.144	22.38	DYAGVFSDAGLTFTSSS(	sp|P01024	1.73E-05	26416.4	20859.4	22665.8	35973.0	26741.4	33844.0	12227.8	12170.6	11944.5
2655	4	401.957	1603.801	3.86	FYHPEKEDGKLNK	sp|P01024	3.74E-06	7096.0	6371.0	7406.9	9384.9	9744.2	9263.1	5255.8	5598.4	5273.7
2698	3	860.109	2577.305	15.17	TMQALPYSTVGNSNNYLI	sp|P01024	0.004173	11800.8	8036.3	7646.3	19165.6	15681.0	22777.2	8698.3	11358.5	7979.2
2872	4	497.504	1985.986	6.88	KVFLDCCNYITELRR	sp|P01024	0.0006	5599.4	6847.1	6169.0	6644.0	8027.3	6673.8	11730.6	9418.1	11538.9
2911	3	797.415	2389.223	18.91	EVVADSVWVDVKDSCVG	sp|P01024	0.001446	13869.3	11652.3	15399.9	18410.5	14344.3	16186.1	23596.6	26542.5	20500.9
2926	3	556.585	1666.733	9.58	VYAYYNLEESCTR	sp|P01024	6.28E-07	12680.1	13653.6	11833.5	17137.5	16669.7	15050.6	6974.7	6171.5	6310.6
3040	2	601.814	1201.614	6.24	NTMILEICTR	sp|P01024	0.000271	6749.0	8885.9	7283.0	8102.4	11351.1	8685.4	3373.8	3176.0	4019.5
3072	3	626.997	1877.970	11.73	EYVLPSFEVIVEPTEK	sp|P01024	3.29E-07	11717.8	9986.9	11222.6	16911.1	15144.5	17045.4	1955.1	1500.0	1201.1
3105	4	641.830	2563.290	11.96	QKPDGVFQEDAPVIHQEI	sp|P01024	5.79E-05	8180.5	11045.7	8242.4	15077.3	16498.7	13834.0	5658.2	5005.5	3884.7
3109	2	439.763	877.511	2.25	LMNIFLK	sp|P01024	3.74E-06	2109.3	2166.1	2147.2	4169.5	5477.4	4409.8	1222.2	1168.8	900.8
3252	2	668.370	1334.726	16.06	APSTWLTAYVVK	sp|P01024	8.82E-08	8282.0	6214.9	6469.9	12965.7	9552.8	11503.5	1235.3	1057.9	959.4
3382	3	568.270	1701.788	5.41	VFLDCCNYITELR	sp|P01024	0.001679	6287.9	5711.9	5857.8	8406.1	6784.0	6646.4	4504.1	4683.3	3788.8
3403	2	568.294	1134.574	14.06	QPSSAFAAFVK	sp|P01024	0.02777	4650.9	3366.3	4256.0	6209.5	4757.8	7050.4	3332.6	4235.4	3889.5
3530	4	1030.771	4119.053	14.43	NNNEKDMALTAFVLISLQI	sp|P01024	0.000318	23824.9	17512.6	23104.3	24751.1	28167.4	23676.4	35673.9	32972.4	33191.7
3556	4	387.704	1546.785	5.74	SDDKVTLEERLDK	sp|P01024	2.09E-08	2196.1	2035.2	1975.7	1764.1	2006.4	1657.9	6764.1	6586.2	6559.1
3702	2	437.225	872.436	4.64	QLANGVDR	sp|P01024	1.01E-07	3018.1	3015.0	3227.5	5064.0	4360.0	5196.1	593.9	656.5	451.9
3711	3	497.927	1490.758	8.39	GQGTLSVVTMYHAK	sp|P01024	1.04E-06	3375.4	4165.1	3367.9	5375.6	5978.2	5980.9	2000.8	1986.6	2027.1
3845	2	436.734	871.453	4.41	QLANGVDR	sp|P01024	2.97E-07	3308.2	3111.2	3470.3	4542.4	4482.7	4168.2	1606.2	1592.4	1458.8
4097	3	893.758	2678.251	11.33	YYGGGYGSTQATFMVFC	sp|P01024	5.99E-07	10283.6	11056.2	11447.1	24392.0	18269.1	22666.9	5313.2	4308.0	4402.8
4468	2	695.353	1388.691	13.12	KQELSEAEQATR	sp|P01024	9.16E-05	3619.8	4011.1	3934.5	6059.0	7185.2	5891.8	3786.3	3472.1	3703.4
4992	4	831.459	3321.807	12.64	QLYNVEATSYALLALLQLI	sp|P01024	0.001919	7193.0	7424.7	7539.2	7928.7	8586.7	10056.7	6761.4	5457.0	5516.8
5064	3	1108.275	3321.805	24.44	QLYNVEATSYALLALLQLI	sp|P01024	0.002818	11484.5	10584.5	11812.5	12380.3	12211.0	15326.1	10197.7	8463.8	8257.1
5468	4	744.636	2974.513	15.52	AEDLVGKSLYVSATVILHS	sp|P01024	2.03E-06	5034.5	4003.2	5062.1	5066.5	4193.0	5357.3	14077.3	14062.0	12521.9
5475	4	1009.538	4034.124	14.96	WEDPGKQLYNVEATSYA	sp|P01024	1.35E-06	10654.9	8726.6	10297.6	7486.4	7047.9	7466.3	16126.8	16312.7	15035.1
5784	4	707.641	2826.533	12.18	QVREPGQDLVVLPLSITTI	sp|P01024	0.001032	4619.8	3161.7	3344.1	5311.8	3821.0	5063.9	7438.3	7656.5	6883.3
5969	3	334.499	1000.475	3.34	NRWEDPGK	sp|P01024	1.16E-07	1308.7	1238.0	1439.4	1727.4	1596.6	1727.5	644.8	611.0	636.7
6115	2	1222.658	2443.302	16.17	EPGQDLVVLPLSITTDFIP	sp|P01024	7.13E-06	9338.8	8663.9	10574.5	19856.3	15181.2	17939.1	4733.6	4634.4	3384.3

6307	3	641.997	1922.970	10.50	DSITTWEILAVSMSDKK	sp|P01024	1.78E-06	4881.6	3586.4	4258.4	9265.9	8256.9	8472.5	1771.6	1156.0	1384.8
6910	3	783.744	2348.209	8.40	NNNEKDMALTAFVLISLQ	sp|P01024	1.66E-08	5554.2	4610.6	5125.8	11431.2	10987.2	12167.1	907.7	1255.1	1044.9
7620	2	1129.057	2256.100	16.95	VQLSNDFDEYIMAIEQTIK	sp|P01024	0.001364	4600.5	3443.8	4430.6	11172.3	7809.8	8750.2	4719.7	4505.4	3846.3
7700	4	313.152	1248.580	3.41	FYHPEKEDGK	sp|P01024	3.07E-05	2568.6	2203.1	2486.2	3834.9	2880.6	3931.5	839.3	815.8	886.7
8820	2	1076.108	2150.201	21.90	QLYNVEATSYALLALQLI	sp|P01024	1.93E-06	6861.1	4205.7	7747.8	12800.7	8789.1	12180.9	896.5	923.9	725.6
9215	4	1007.246	4024.954	8.73	AYYENSPQQVFSTEFEVI	sp|P01024	0.000932	6406.0	6562.3	8269.5	6977.7	6617.0	7793.9	7504.8	7831.1	8745.9
9342	3	739.750	2216.227	21.11	VFSLAVNLIAIDSQVLCGA	sp|P01024	3.30E-07	5418.8	4840.8	5676.0	12230.7	10865.0	10944.0	1151.9	896.4	633.7
9909	3	917.112	2748.313	15.17	YFKPGMPFDLMVFVTNPI	sp|P01024	3.32E-06	5529.4	4364.1	4454.5	11678.1	14188.2	12400.7	5169.6	4544.8	4412.7
11070	5	824.818	4119.055	6.51	NNNEKDMALTAFVLISLQ	sp|P01024	9.67E-07	2024.6	1731.6	1951.1	2581.1	2366.9	2425.2	4001.8	3598.8	3430.9
11484	3	955.181	2862.522	11.59	WEDPGKQLYNVEATSYA	sp|P01024	1.14E-09	3738.3	3684.7	3747.4	6022.6	6563.8	6039.6	1082.8	1238.0	1197.2
12272	3	824.738	2471.191	20.11	GICVADPFEVTVMQDFFI	sp|P01024	7.38E-08	2030.9	1928.7	2501.9	5099.5	4796.0	5906.3	551.8	469.0	425.7
13552	3	1000.505	2998.492	11.59	EYVLPSFEVIVEPTEKFYY	sp|P01024	1.29E-05	2631.2	1645.3	2596.2	1916.4	1560.8	1906.5	5236.5	5299.0	5021.0
14888	3	1094.887	3281.638	22.96	QDSLSSQNQLGVLPLSW	sp|P01024	0.000106	3149.5	2950.7	3012.8	8674.4	7489.8	6887.7	1106.0	2281.2	1683.3
16170	3	937.509	2809.505	15.91	QVREPGQDLVVLPLSITTI	sp|P01024	4.63E-07	1108.8	1106.2	1142.3	1890.3	1832.1	1685.0	4218.4	3557.7	4012.8
16370	4	716.638	2862.522	7.79	WEDPGKQLYNVEATSYA	sp|P01024	8.80E-10	1392.5	1401.6	1367.3	2321.1	2342.5	2257.7	312.5	345.8	361.7
16781	3	720.646	2158.916	10.94	QCQDLGAFTESMVVFGC	sp|P01024	0.002006	1100.3	757.8	1361.5	1990.2	1722.1	1980.0	774.5	846.6	733.3
17486	5	807.832	4034.126	6.78	WEDPGKQLYNVEATSYA	sp|P01024	6.67E-06	1493.8	1210.0	1405.3	1058.4	946.2	1024.2	2224.1	2197.4	2006.5
24450	4	985.034	3936.108	16.05	EPGQDLVVLPLSITTDFIP	sp|P01024	0.01031	1550.4	1603.7	1660.3	1643.1	1342.1	1537.9	1448.6	1347.2	1135.5
25219	3	753.369	2257.086	12.78	VQLSNDFDEYIMAIEQTIK	sp|P01024	0.008401	348.6	287.0	334.2	721.6	662.4	630.1	498.5	396.1	277.7
29328	4	880.954	3519.789	15.65	DMALTAFVLISLQEAKDIC	sp|P01024	0.007729	450.2	531.0	709.4	1245.3	863.5	1015.1	349.4	581.3	275.0
10288	3	682.349	2044.025	10.75	SYFPESWLWEVHLVPR	sp|P01031	0.025497	4637.2	3672.1	4919.2	5847.0	4718.1	6041.2	5873.1	6091.5	6083.9
15331	3	947.141	2838.400	13.04	YGGGFYSTQDTINAIEGL	sp|P01031	7.28E-05	1919.0	1362.8	1581.6	2968.0	2779.6	3324.7	1264.1	981.0	1128.2
20678	3	705.692	2114.055	12.16	YIYPLDSLTWIEYWPR	sp|P01031	9.96E-05	665.4	621.7	780.9	1188.7	1185.0	1255.4	407.4	534.5	329.4
22106	3	872.179	2613.514	16.17	GLLVGEILSAVLSQEGINI	sp|P01031	0.009449	799.0	777.2	1085.3	1372.6	1375.7	1447.9	828.6	1021.0	919.3
24458	5	856.024	4275.083	1.99	GSAEAELMSVVPVFYVFI	sp|P01031	0.000559	933.7	879.2	809.3	1179.1	1274.6	1270.4	1739.0	1468.3	1225.1
29129	3	840.453	2518.336	15.38	VDDGVASFVLNLPSGVT\	sp|P01031	2.61E-07	695.3	714.7	609.5	1405.6	1362.3	1438.2	338.7	349.2	266.2
489	2	626.299	1250.584	15.06	TVGSDTFYSFK	sp|P01042	0.007536	40396.9	48634.0	41782.4	47470.1	48703.2	45517.8	55694.4	57502.7	72902.8
623	3	838.078	2511.213	19.91	IASFSQNCDIYPGKDFVQ	sp|P01042	0.002753	92777.8	117151.1	85909.1	107999.2	103375.7	100865.7	157298.7	163969.3	131009.0
668	3	505.558	1513.653	9.40	TWQDCEYKDAAK	sp|P01042	0.017559	20036.6	29697.6	27950.2	24576.7	23230.6	25574.8	33971.9	39140.3	34705.0
713	2	538.739	1075.462	14.02	EGDCPVQSGK	sp|P01042	0.016331	25551.5	21018.0	24890.5	26851.8	23569.1	29941.8	36991.3	40467.9	35348.0
741	2	515.773	1029.531	6.94	YFIDFVAR	sp|P01042	4.01E-05	15617.5	15832.6	18580.4	18847.5	17326.0	19386.8	26998.4	27160.4	25823.0
761	2	502.289	1002.563	8.49	QVVAGLNFR	sp|P01042	0.034526	15426.2	17744.7	15279.7	12803.0	19505.9	14623.6	19705.6	22700.0	25809.4
1485	2	583.280	1164.546	16.06	AATGECTATVGK	sp|P01042	0.003873	13251.3	11546.1	14150.3	16659.0	13588.7	17074.0	10492.1	10526.0	11498.9
1749	2	692.336	1382.657	16.06	ENFLFLTPDCK	sp|P01042	1.46E-05	16114.8	10907.9	14553.6	23665.5	23366.3	22751.3	8203.5	8516.5	7678.9

2226	3	537.256	1608.747	8.73	YEIKEGDCPVQSGK	sp|P01042	3.09E-05	8383.0	9040.8	7451.3	8409.5	8515.4	6917.9	15852.2	16257.9	15874.2
2823	2	350.722	699.429	1.46	VQVVAGK	sp|P01042	0.02762	6455.8	5606.2	7425.3	6891.0	5740.1	7155.5	10432.4	9778.1	10508.6
2874	3	386.882	1157.625	2.10	KYFIDFVAR	sp|P01042	0.014978	2317.6	2777.0	2557.7	2603.8	3483.2	2489.2	3558.9	3833.3	5174.7
2989	3	814.374	2440.100	16.11	SLWNGDTGECTDNAYIDI	sp|P01042	0.001263	18694.5	14597.1	16241.2	20463.2	17061.0	17389.1	22491.6	19576.0	23246.9
3386	4	628.811	2511.215	11.90	IASFSQNCDIYPGKDFVQI	sp|P01042	0.007532	8594.9	11163.4	8133.3	10702.0	9766.1	9725.3	14971.8	15651.0	11697.7
3738	3	272.505	814.492	4.15	AVDAALKK	sp|P01042	0.029063	2962.6	2401.2	3081.4	3659.5	2738.5	3158.6	4419.1	4231.2	5235.0
4748	4	591.558	2362.203	12.54	KLGQSLDCNAEVYVVPW	sp|P01042	0.000112	2179.0	3144.3	2902.8	2736.8	2873.5	2209.0	7048.0	6639.5	9641.0
7402	3	300.822	899.445	1.30	CPGRPWK	sp|P01042	0.005902	1535.0	1251.7	1505.6	1414.0	1093.1	1383.8	2442.4	2595.5	2540.9
266	2	695.311	1388.607	14.11	SSEDPNEDIVER	sp|P01591	0.016262	101308.2	140875.8	99598.9	95682.3	96601.1	94321.1	135552.3	125785.0	148637.8
467	3	427.882	1280.624	7.79	FVYHLSDLCK	sp|P01591	0.014793	30259.8	33132.6	27385.3	26731.5	30634.0	24045.1	34019.0	30321.7	34455.4
746	2	828.920	1655.825	16.90	CYTAVVPLVYGGETK	sp|P01591	0.009354	59050.2	55281.7	68731.3	49252.0	62271.3	52740.8	71622.6	73884.5	68033.2
946	2	400.748	799.482	1.45	IVLVDNK	sp|P01591	0.005765	11232.6	17155.3	11525.9	11584.8	11704.6	10736.7	7313.7	7610.8	9475.7
1549	4	353.187	1408.719	6.06	FVYHLSDLCKK	sp|P01591	0.001274	6878.8	8605.7	7200.0	5481.1	7118.1	6419.2	9358.6	10641.3	11274.6
2066	2	791.843	1581.672	11.32	MVETALTPDACYPD	sp|P01591	0.017912	17771.1	21897.6	21351.6	20759.0	18370.9	20856.4	26409.8	32133.4	26331.6
2336	2	820.407	1638.799	14.90	CYTAVVPLVYGGETK	sp|P01591	0.002061	18475.0	16508.4	19277.1	19033.3	15929.3	16450.1	21967.6	24379.0	22737.3
2186	2	525.257	1048.499	4.06	ITCQGDSLR	sp|P01714	0.00396	6792.2	6909.6	6474.9	6672.1	4817.5	5637.0	6977.8	6211.2	6731.2
9005	3	636.329	1905.966	11.54	ALEWLALIYWDDDKR	sp|P01817	0.000211	4251.2	2995.8	3437.8	3319.9	2603.3	3057.5	1575.7	1568.5	1322.9
30432	3	705.405	2113.194	11.14	LDIQGTGQLLFSVVINQLF	sp|P01833	3.03E-05	56.7	23.3	97.5	122.4	110.6	87.3	1233.7	1100.2	1430.4
54	2	435.183	868.351	5.54	SFNRGEC	sp|P01834	0.006454	114163.8	104808.3	129491.5	102796.9	84974.0	112780.9	159031.3	163395.2	150243.1
233	3	712.662	2134.965	20.11	VDNALQSGNSQESVTEQ	sp|P01834	4.76E-05	193575.1	226953.9	180758.6	196682.6	221982.0	190365.6	82596.2	96570.5	74943.2
234	4	536.026	2140.077	14.70	HKVYACEVTHQGLSSPV	sp|P01834	3.62E-06	120022.2	118367.0	108194.4	110524.6	115131.3	104817.0	175303.2	172711.1	167425.6
608	2	751.884	1501.753	16.06	DSTYSLSSTLTLSK	sp|P01834	5.76E-08	119728.5	112637.4	121686.4	137241.9	145661.0	138270.2	54702.3	47400.6	44840.8
1055	2	1068.489	2134.964	19.90	VDNALQSGNSQESVTEQ	sp|P01834	0.000393	78444.8	101876.0	62205.5	77857.2	110025.4	74778.2	28774.5	34881.3	28976.8
2569	2	435.675	869.335	1.46	SFNRGEC	sp|P01834	0.001035	4528.3	4163.0	4893.6	4681.2	3745.4	4171.8	5497.5	5666.6	5700.6
6497	4	1041.009	4160.007	14.38	VQWKVDNALQSGNSQES	sp|P01834	8.35E-10	1981.3	2157.9	2104.9	1050.0	1303.2	1644.7	102982.0	98524.5	78862.4
8308	2	435.675	869.335	1.46	SFNRGEC	sp|P01834	0.001588	952.1	904.0	1045.7	809.6	767.1	883.5	1065.4	1309.4	1277.1
13199	4	931.983	3723.901	11.82	TVAAPSVFIFPPSDEQLKS	sp|P01834	5.54E-08	8725.8	6942.1	8196.2	7355.7	4924.0	6346.5	61155.4	58626.9	56887.2
326	3	559.940	1676.798	14.06	FNWYVDGVEVHNAK	sp|P01857	0.002222	86955.8	89440.0	83022.7	60851.7	78089.5	62669.6	44020.5	44527.0	60304.7
428	3	713.682	2138.024	20.90	TPEVTCVVVDVSHEDPEV	sp|P01857	1.34E-05	159247.4	155321.7	177716.1	113453.6	123262.7	114166.3	66886.9	72416.3	81203.1
513	2	593.828	1185.641	16.06	GPSVFPLAPSSK	sp|P01857	0.023927	59646.6	64488.2	62000.6	42925.5	33942.6	53282.1	46536.6	44954.9	54554.9
935	2	937.466	1872.917	14.90	TTPPVLDSDGSFFLYSK	sp|P01857	0.008322	130907.6	84358.1	111195.8	86147.8	69914.0	91341.6	64507.4	59930.6	57398.6
1063	4	711.871	2843.455	14.78	THTCPPCPAPELLGGPSV	sp|P01857	7.26E-05	156076.7	132716.8	151937.2	99348.4	87822.1	100913.9	83221.5	84529.0	78336.6
1339	5	1343.470	6712.315	14.08	DYFPEPVTVSWNSGALTS	sp|P01857	0.002428	223058.4	169016.8	209021.9	211277.5	236451.2	214047.7	127876.8	162331.7	126009.8
1914	5	667.735	3333.641	7.35	SCDKTHTCPPCPAPELLG	sp|P01857	0.002161	66570.3	68835.1	68952.5	52416.3	66578.4	50045.1	86096.1	77705.6	78567.1

4022	6	1119.727	6712.315	9.84	DYFPEPVTVSWNSGALT	sp|P01857	0.001655	42589.3	33126.1	41122.4	38062.1	46843.7	41074.3	25419.3	28443.9	25272.9
8191	6	1336.836	8014.972	10.63	STSGGTAALGCLVKDYF	sp|P01857	3.97E-08	5364.8	4729.6	7006.6	3447.2	3243.4	4392.2	60417.9	55862.8	56459.0
1190	4	554.551	2214.174	12.54	VVSVLTVVHQDWLNGKE	sp|P01859	0.017326	24507.0	25013.9	26424.2	18620.7	27527.9	17882.5	25644.8	26986.2	26014.0
1309	2	412.748	823.482	5.52	GLPAPIEK	sp|P01859	0.047624	10300.8	11222.0	11026.1	8840.1	11150.1	8426.9	9828.6	8842.2	10132.8
1314	3	598.669	1792.986	11.89	VVSVLTVVHQDWLNGK	sp|P01859	0.000203	29268.3	33799.8	24253.7	19344.7	16792.7	18698.9	13706.5	15864.6	13783.6
2409	3	598.997	1793.970	11.85	VVSVLTVVHQDWLNGK	sp|P01859	0.000193	25118.2	18367.0	23610.5	21297.2	17753.0	21526.4	9406.4	10065.2	7197.9
3819	3	970.140	2907.398	20.78	CCVECPPCPAPPVAGPS	sp|P01859	0.017773	48575.9	42757.1	40224.6	38720.8	31852.5	39775.1	38478.2	40644.5	31946.9
4219	4	554.304	2213.187	4.12	VVSVLTVVHQDWLNGKE	sp|P01859	2.43E-07	3661.4	3168.2	3925.2	2062.6	1803.2	1526.1	7378.7	7350.6	7067.0
3076	3	452.551	1354.632	10.85	TPLGDTTHTCPR	sp|P01860	1.58E-05	5040.0	4891.2	4673.8	2909.9	2421.5	3067.9	4621.1	4650.8	4106.6
3273	2	593.753	1185.492	13.02	SCDTPPPCPR	sp|P01860	6.75E-07	15565.2	14435.6	13980.9	9311.4	9213.0	9074.3	16370.6	15499.6	14054.5
11230	5	1341.064	6700.284	7.14	DYFPEPVTVSWNSGALT	sp|P01860	0.000814	16235.9	10765.8	14327.3	9392.2	8382.1	8434.8	8019.2	8187.5	7149.6
1453	3	981.825	2942.453	20.78	YGPPCPSCPAPEFLGGP	sp|P01861	0.00028	149968.8	124585.6	135172.9	89079.1	68258.6	85954.2	142236.7	142224.4	124135.9
2287	2	951.469	1900.923	14.90	TTPPVLDSDGSFFLYSR	sp|P01861	0.001344	45676.1	33100.4	41127.5	27694.6	24367.6	25684.6	29196.5	26240.7	24984.9
2356	2	415.735	829.456	2.24	GLPSSIEK	sp|P01861	4.82E-05	7658.4	9566.9	7501.9	4321.9	5290.3	4610.6	8051.5	7778.4	7895.2
3135	4	1264.894	5055.545	14.51	DYFPEPVTVSWNSGALT	sp|P01861	0.00187	67597.5	60340.5	71480.1	58661.9	40792.5	50353.3	43111.3	47238.5	39784.5
4696	4	736.621	2942.455	12.98	YGPPCPSCPAPEFLGGP	sp|P01861	8.39E-05	23554.0	20088.6	21726.0	14061.8	11076.2	13072.5	27897.9	25612.0	23469.2
171	3	572.960	1715.858	14.90	FTCTVTHTDLPSPLK	sp|P01871	0.007228	253836.8	281329.4	245407.2	207048.0	253816.2	198301.0	200459.7	181416.8	232463.7
216	2	625.322	1248.630	16.06	LICQATGFSPR	sp|P01871	0.033649	109828.4	131196.6	129926.3	95305.8	130828.3	97236.0	117416.2	119223.5	122112.1
223	2	515.297	1028.579	10.87	QIQVSWLR	sp|P01871	0.005851	77751.4	101564.6	97213.6	68981.3	83046.0	70956.8	72033.1	82932.9	69625.0
252	2	809.408	1616.802	19.90	QVGSGVTTDQVQAEAK	sp|P01871	0.000627	163535.1	224313.0	161291.2	136281.4	168191.5	134776.0	86298.5	82612.6	98125.1
272	2	455.215	908.415	6.94	DGFFGNPR	sp|P01871	0.00245	50337.1	59566.7	56041.1	38659.1	46816.1	38199.9	52028.0	50450.8	65666.8
307	2	386.720	771.425	1.43	EQLNLR	sp|P01871	0.000545	28033.0	30382.5	29477.6	25274.9	27956.7	23764.8	25027.4	21993.8	23532.4
375	4	931.692	3722.737	20.69	YFAHSILTVSEEEWNTGE	sp|P01871	1.76E-06	406571.2	327774.2	417019.5	371312.7	331075.0	348221.3	140584.6	131374.6	111615.3
451	4	444.259	1773.009	2.15	GVALHRPDVYLLPPAR	sp|P01871	0.011526	104472.1	139641.0	93614.0	76673.8	93443.2	75180.8	85685.2	91050.2	110305.5
533	2	450.769	899.524	7.31	VSVFVPPR	sp|P01871	2.46E-05	54633.7	56747.5	53950.7	41545.3	42916.6	41704.5	43529.7	42045.1	48359.7
565	2	431.766	861.518	7.32	VTSTLTIK	sp|P01871	8.09E-05	33335.1	35592.0	31013.4	24135.4	30270.9	24933.5	17189.0	17063.4	16914.8
584	5	842.606	4207.995	11.94	YFAHSILTVSEEEWNTGE	sp|P01871	4.68E-10	140827.2	141872.0	124651.7	62704.4	61686.1	67888.3	348254.1	336367.9	341527.3
657	5	546.479	2727.360	8.43	YAATSQVLLPSKDVMQG	sp|P01871	0.023522	50135.2	67794.8	58534.7	40243.4	51996.2	36762.1	58784.1	58398.9	73977.6
663	2	428.233	854.451	7.48	GQPLSPEK	sp|P01871	0.022391	35556.3	30769.5	36961.9	28259.0	23616.7	29636.8	27437.6	27944.5	29588.5
679	3	539.941	1616.803	11.83	QVGSGVTTDQVQAEAK	sp|P01871	0.000265	34981.8	45685.2	35259.3	28342.2	29453.8	28469.4	18573.0	18349.2	20988.9
879	2	639.359	1276.703	16.11	YAATSQVLLPSK	sp|P01871	1.73E-09	38173.5	40504.2	40073.5	30339.7	31041.3	28380.7	16670.5	16534.3	16705.6
934	4	694.847	2775.359	16.97	YAATSQVLLPSKDVMQG	sp|P01871	0.009148	39575.2	51768.2	48740.4	38474.3	40745.3	47203.3	57306.2	58057.6	72560.8
1005	2	819.470	1636.926	11.95	VFAIPPSFASIFLTK	sp|P01871	0.001274	147759.6	121943.9	123640.0	129536.6	103046.1	127086.8	88474.7	86411.0	74071.7
1228	3	490.562	1468.663	9.85	DVMQGTDEHVVCK	sp|P01871	0.005038	23214.6	20083.7	23325.4	15866.5	12590.4	16584.9	10698.6	11086.6	11143.6

1432	2	406.735	811.456	2.03	QTISRPK	sp|P01871	0.001285	8672.0	8094.7	9557.2	7776.2	7378.0	7719.7	7713.5	7434.6	7958.1
1460	3	518.264	1551.770	9.29	YVTSAPMPEPQAPGR	sp|P01871	7.72E-05	26174.7	30009.8	24472.7	16872.3	20397.5	17998.8	14101.5	15757.6	15540.5
1475	2	693.922	1385.830	13.06	NVPLPVIAELPPK	sp|P01871	0.000288	193482.3	174820.1	184747.6	136330.0	123903.1	144094.5	156556.7	157604.9	136359.0
1480	3	417.217	1248.631	7.09	LICQATGFSPR	sp|P01871	0.01017	9045.9	10275.4	10243.8	7637.8	9297.2	7750.0	8927.3	9612.2	9810.4
1886	2	506.784	1011.553	6.87	QIQVSWLR	sp|P01871	0.000315	7885.9	6936.8	7593.3	6292.2	5964.7	7273.4	8572.3	8420.5	7850.2
2063	3	546.649	1636.926	16.11	VFAIPPSFASIFLTK	sp|P01871	0.000994	35186.3	28251.0	31260.4	30852.3	23106.2	29274.2	19948.4	20594.1	17435.3
2160	2	441.715	881.415	6.07	ESGPTTYK	sp|P01871	6.67E-05	38686.2	35279.2	42243.4	34953.2	30010.4	31608.5	23885.5	23855.1	23122.4
2192	3	644.661	1930.962	12.63	EGKQVGSGVTTDQVQAE	sp|P01871	2.47E-07	5773.9	3727.6	4179.2	2758.0	3538.0	2188.9	35863.1	39890.7	34920.4
2307	3	915.114	2742.319	22.38	ESATITCLVTGFSPADVF\	sp|P01871	0.000941	28523.7	27329.7	32655.6	33152.9	28140.8	32236.4	36738.1	35872.7	33669.1
2474	2	800.895	1599.776	17.90	QVGSGVTTDQVQAEAK	sp|P01871	7.52E-05	19348.1	22802.7	24744.3	17522.9	21778.9	17025.4	8850.0	9296.1	11314.5
2785	4	1053.006	4207.995	16.89	YFAHSILTVSEEEWNTGE	sp|P01871	2.83E-08	35754.4	33127.0	28645.2	11363.6	12659.5	14296.0	86930.3	84544.8	82745.9
3864	3	462.951	1385.831	11.32	NVPLPVIAELPPK	sp|P01871	0.001136	30914.8	26482.8	30038.9	21836.2	18947.2	22813.4	23224.8	25233.1	21963.0
7487	2	808.846	1615.678	15.06	ESDWLGQSMFTCR	sp|P01871	5.67E-05	1932.9	1378.0	1785.9	4949.6	4981.9	5737.3	763.2	1181.1	1100.5
21239	4	686.587	2742.319	14.66	ESATITCLVTGFSPADVF\	sp|P01871	0.027259	792.4	657.6	991.3	896.2	688.2	836.1	1065.8	1107.5	914.9
26043	4	1070.820	4279.253	3.19	VFAIPPSFASIFLTKSTKLT	sp|P01871	3.39E-05	3547.4	3148.6	4701.8	1447.7	1269.3	1274.8	3781.5	5155.6	5035.9
40762	3	298.820	893.437	2.14	VQHPNGNK	sp|P01871	0.0296	816.8	159.5	147.4	789.6	801.4	623.5	192.6	298.7	168.7
64	2	918.479	1834.944	19.95	QEPSQGTTTFAVTSILR	sp|P01876	0.048493	992146.2	797441.5	840272.8	785830.3	662289.0	730525.1	652990.7	731096.2	741465.5
100	3	865.048	2592.123	23.17	DLCGCYSVSSVLPGCAEI	sp|P01876	0.003918	691078.6	434988.0	542777.5	436966.4	409886.4	476480.1	310175.4	309768.7	251444.4
211	4	573.040	2288.129	6.81	KGDTFSCMVGHEALPLAI	sp|P01876	0.020965	187507.3	213123.6	168709.1	156868.4	145718.5	161836.3	183506.7	167698.7	207123.2
213	4	879.405	3513.592	18.44	SAVQGGPPERDLCGCYSV	sp|P01876	3.81E-08	291903.0	259863.9	305992.5	145334.9	168646.7	141576.3	819593.0	839972.2	775769.4
227	4	585.041	2336.134	11.81	KGDTFSCMVGHEALPLAI	sp|P01876	0.028579	118864.4	133963.5	112113.2	127506.4	135515.3	122511.9	148614.1	141605.1	133274.5
282	3	514.248	1539.724	13.88	DASGVTFTWTPSSGK	sp|P01876	0.03871	74567.5	77743.7	72656.0	60800.8	66261.6	55856.3	95999.4	81860.5	68207.1
376	3	1056.844	3167.512	18.90	NFPPSQDASGDLYTTSS(	sp|P01876	0.008971	########	#######	#######	1168159.0	1032524.9	########	########	1358872.0	########
510	5	458.633	2288.131	4.10	KGDTFSCMVGHEALPLAI	sp|P01876	0.000736	53776.4	51303.0	52903.9	46498.2	44838.7	46644.8	55052.0	55143.0	60468.1
526	4	1195.348	4777.361	18.11	VFPLSLCSTQPDGNVVIA(	sp|P01876	0.037543	649930.7	630640.9	808220.2	736672.2	591418.6	720532.6	804813.1	892859.7	715925.3
541	2	909.966	1817.918	17.90	QEPSQGTTTFAVTSILR	sp|P01876	0.032087	133800.4	134169.0	143416.2	151792.7	146264.2	145849.7	124100.5	143073.8	135827.9
712	4	792.885	3167.512	17.18	NFPPSQDASGDLYTTSS(	sp|P01876	0.009971	471924.4	423435.3	444605.0	396243.6	336051.2	345681.1	447324.4	489670.0	418341.5
1725	4	649.038	2592.124	12.43	DLCGCYSVSSVLPGCAEI	sp|P01876	0.001052	28435.7	20175.1	27847.8	19640.0	20951.5	19213.1	11878.5	14664.9	13658.2
1789	5	956.480	4777.362	19.42	VFPLSLCSTQPDGNVVIA(	sp|P01876	0.03415	148072.5	140433.9	179674.9	166196.2	129219.2	156595.5	158623.1	176815.8	146433.0
3053	4	691.615	2762.429	12.09	QEPSQGTTTFAVTSILRV/	sp|P01876	4.84E-06	26519.3	23994.8	27594.2	21315.5	18145.8	21088.3	11188.1	11257.9	10558.2
3411	4	1195.347	4777.357	18.55	VFPLSLCSTQPDGNVVIA(	sp|P01876	0.001067	94628.0	77025.3	97803.3	92545.2	78850.0	86590.6	66699.1	65659.5	64077.9
7126	3	879.118	2634.333	11.90	QEPSQGTTTFAVTSILRV/	sp|P01876	0.005485	10895.6	7373.4	10220.6	7687.9	6192.1	6908.5	4888.6	5541.1	3397.9
24274	6	797.234	4777.361	16.23	VFPLSLCSTQPDGNVVIA(	sp|P01876	0.002406	2720.7	2063.7	2928.0	2586.3	2207.3	2711.9	2155.7	2039.9	2195.2
30171	3	1593.459	4777.355	16.28	VFPLSLCSTQPDGNVVIA(	sp|P01876	0.003823	5227.6	4350.9	5442.1	5183.5	5064.6	5682.3	3694.9	3337.0	2931.5

309	2	756.853	1511.691	18.95	DASGATFTWTPSSGK	sp|P01877	7.79E-06	114961.6	120323.1	126510.8	120778.2	112101.7	128751.8	188132.1	167775.6	170906.6
829	4	988.192	3948.737	12.37	DLCGCYSVSSVLPGCAQ	sp|P01877	2.32E-05	126257.5	97489.6	114014.8	99475.1	87150.5	92713.2	48474.1	46590.7	46804.7
1958	5	975.048	4870.205	11.09	SAVQGPPERDLCGCYSV	sp|P01877	3.48E-07	38453.1	37737.0	33995.7	21580.1	28309.9	21099.3	113082.8	121610.2	117840.7
1515	2	480.270	958.526	7.89	TAVNALWGK	sp|P02042	0.040196	6530.4	7253.4	7203.3	7386.7	11159.2	7754.9	9659.4	8696.7	9955.1
457	4	605.566	2418.233	11.32	EVDLKDYEDQQKQLEQV	sp|P02671	6.13E-06	45494.5	65502.7	50292.4	31062.4	32849.7	35699.9	107527.4	125884.4	107176.1
464	2	514.757	1027.499	7.05	NSLFEYQK	sp|P02671	0.004584	27175.6	28831.5	34721.1	22245.2	26991.8	27581.3	38549.8	35936.2	39847.1
504	2	786.843	1571.672	18.90	GGSTSYGTGSETESPR	sp|P02671	0.000648	96864.0	86817.5	90138.8	86260.3	69464.4	82365.4	62941.2	59677.0	58392.0
529	2	760.871	1519.728	15.11	GLIDEVNQDFTNR	sp|P02671	0.000289	69642.9	59278.3	59831.3	50877.1	41789.1	44039.9	51240.9	53665.3	55249.5
538	2	505.760	1009.506	11.87	VTSGSTTTTR	sp|P02671	0.004198	23892.8	22302.1	26433.1	20133.6	16005.6	19215.3	29208.9	46180.6	32779.0
697	2	464.778	927.541	7.07	QLEQVIAK	sp|P02671	3.21E-05	21591.1	24013.2	21601.3	18747.0	20287.1	16839.0	10303.7	10936.2	10705.3
709	3	507.584	1519.729	10.13	GLIDEVNQDFTNR	sp|P02671	0.001687	27914.8	22310.0	23572.6	20602.7	16766.4	17909.7	21885.2	25919.8	23893.5
726	3	501.253	1500.738	3.41	MELERPGGNEITR	sp|P02671	1.43E-05	19361.2	20147.1	19865.5	18842.7	23140.0	18866.3	34531.6	32858.5	36333.6
842	3	577.588	1729.742	10.18	GDFSSANNRDNTYNR	sp|P02671	2.88E-09	20120.3	18291.6	20136.9	14213.8	14853.9	13625.2	52667.8	49867.9	53454.8
914	3	369.561	1105.663	7.23	VQHIQLLQK	sp|P02671	0.001853	20281.6	24156.3	17683.7	14309.3	20805.3	15363.7	10180.5	9862.8	9929.4
1072	3	503.908	1508.703	7.40	EVDLKDYEDQQK	sp|P02671	0.009663	23952.3	30193.0	26339.0	24831.1	30748.4	22016.1	17643.2	16730.2	19833.2
1129	4	669.521	2674.053	6.79	HQSACKDSDWPFCSDEI	sp|P02671	8.25E-06	43675.4	33178.8	43098.7	25711.8	21019.1	26542.5	67625.6	70372.5	65745.8
1450	2	570.781	1139.547	15.06	GSESGIFTNTK	sp|P02671	0.001963	13287.7	16433.1	12242.4	9706.5	11729.6	9869.8	17185.0	19717.2	17269.7
1908	3	807.084	2418.230	16.88	EVDLKDYEDQQKQLEQV	sp|P02671	0.000162	14478.6	24470.6	16536.7	9738.4	12555.5	12732.1	35070.0	41883.7	31407.1
2019	3	485.252	1452.734	6.01	MELERPGGNEITR	sp|P02671	5.82E-06	11660.2	10410.1	11069.1	7906.9	8048.6	8130.7	13665.3	13375.3	13443.3
2033	3	481.268	1440.782	8.10	MKPVPDLVPGNFK	sp|P02671	0.000652	14200.7	17811.0	13859.2	11327.2	13361.2	10644.0	21247.4	22346.1	29391.4
2293	3	655.289	1962.844	19.11	NPSSAGSWNSGSSGPGS	sp|P02671	2.90E-05	16037.8	20664.4	19384.5	14890.0	15018.3	13548.0	8849.9	9641.8	9498.8
2616	4	472.476	1885.874	3.89	HRHPDEAAFFDTASTGK	sp|P02671	2.37E-05	9030.4	10700.1	10429.9	7148.9	6017.8	6962.3	13742.1	15552.4	15985.1
2676	4	880.134	3516.505	11.51	GGSTSYGTGSETESPRN	sp|P02671	1.95E-05	25014.6	19372.6	26280.6	15043.7	17677.4	15542.8	35772.2	41754.8	40481.7
2894	4	345.943	1379.743	6.44	TVIGPDGHKEVTK	sp|P02671	0.001661	4479.4	3976.1	4747.9	3049.0	3049.1	3344.9	2941.1	2918.2	3163.2
2966	2	484.216	966.417	7.63	GDFSSANNR	sp|P02671	0.000287	21422.7	19232.9	22824.2	18085.9	15380.6	16723.5	16053.1	15951.4	14700.9
3061	2	368.648	735.281	5.58	CPSGCR	sp|P02671	0.012928	14360.9	12203.1	15375.1	11090.0	9935.5	12007.8	13991.9	14755.3	11189.2
3137	2	982.377	1962.740	15.90	DSDWPFCSDEDWNYK	sp|P02671	4.04E-06	44159.7	39722.0	47303.7	37548.2	31868.9	34896.2	19154.4	18149.6	16821.5
3495	2	595.776	1189.538	14.02	QFTSSTSYNR	sp|P02671	0.013569	6085.2	6987.2	6570.9	5010.9	4260.7	4752.6	5699.4	5120.3	4608.5
3516	4	497.258	1985.004	3.19	NNKDSHSLTTNIMEILR	sp|P02671	0.04713	4454.7	7952.3	4770.4	4380.3	9056.2	4033.7	11235.0	11927.6	9736.5
3518	3	625.991	1874.952	7.29	GLIDEVNQDFTNRINK	sp|P02671	6.95E-10	1193.4	1061.6	1219.9	772.6	903.5	730.6	26053.3	21333.4	20822.8
3636	2	391.676	781.336	3.14	DNTYNR	sp|P02671	0.00629	10086.0	8254.2	9908.9	7920.7	6657.5	7895.1	4965.4	7993.4	5094.1
3669	3	367.549	1099.626	2.05	RLEVDIDIK	sp|P02671	0.012686	2716.0	4958.4	3100.9	3122.7	5353.6	2666.4	1532.1	1562.8	2310.7
3833	3	655.254	1962.741	11.78	DSDWPFCSDEDWNYK	sp|P02671	1.27E-05	16225.6	15521.8	18681.4	13100.2	10855.6	13810.5	7480.2	7335.6	6844.9
5093	3	577.916	1730.726	10.27	GDFSSANNRDNTYNR	sp|P02671	0.000305	4784.1	4089.7	4464.5	4024.0	3551.7	4176.6	4741.1	4947.6	5261.0

6797	3	894.344	2680.009	17.64	DSDWPFCSDEDWNYKCI	sp|P02671	1.11E-07	5288.5	4045.1	4358.1	1851.3	1098.4	1457.5	22011.3	23156.0	19198.1
12487	3	1043.474	3127.400	15.90	EVVTSEDGSDCPEAMDL	sp|P02671	0.036221	4608.0	4111.3	3985.3	4071.1	3717.5	4408.4	2738.0	2334.6	2286.5
226	2	422.749	843.483	2.43	SILENLR	sp|P02675	8.53E-05	38908.1	47692.9	40391.2	30688.4	30602.0	26288.0	51976.0	53164.5	62626.3
292	2	654.813	1307.612	17.06	QGFGNVATNTDGK	sp|P02675	0.000325	97233.5	89078.2	97368.6	72352.5	60255.3	73280.5	96306.8	85156.7	94099.8
304	5	817.610	4083.015	7.25	KAPDAGGCLHADPDLGV	sp|P02675	0.003639	232715.1	233735.1	282582.9	194554.1	192135.9	219343.5	265691.5	218599.0	213615.1
386	2	620.264	1238.513	11.98	EDGGGWWYNR	sp|P02675	0.000693	57997.1	69438.7	57875.4	42350.2	41919.9	40400.2	73278.2	70267.2	57982.2
424	2	490.726	979.437	13.02	QDGSVDFGR	sp|P02675	0.000383	30801.3	38702.7	34226.2	24346.4	22128.4	23367.1	35855.7	36010.4	45284.5
595	3	850.079	2547.216	20.91	TPCTVSCNIPVVSGKECE	sp|P02675	1.20E-05	75043.9	104950.0	75762.1	48419.9	45106.3	57911.4	179848.0	167057.4	179916.2
598	2	359.217	716.419	2.46	ISQLTR	sp|P02675	8.32E-05	16795.1	14959.2	16295.3	11813.4	11507.6	12978.6	17166.1	19301.7	19378.5
665	2	893.400	1784.785	13.90	NYCGLPGEYWLGNDK	sp|P02675	0.00262	103946.1	89503.1	85529.5	71287.3	67748.5	80635.0	61169.8	68902.1	64906.1
711	4	592.798	2367.163	12.60	AHYGGFTVQNEANKYQIS	sp|P02675	4.53E-05	39717.2	44260.0	39538.1	30100.7	37933.9	30227.4	77540.7	69518.2	81541.8
782	4	969.970	3875.850	6.62	DNENVVNEYSSELEKHQI	sp|P02675	4.22E-06	99172.4	88686.0	105347.7	76510.5	63100.2	70627.6	156510.0	147242.8	151202.7
991	3	512.580	1534.719	6.03	AHYGGFTVQNEANK	sp|P02675	0.000175	27122.6	29840.4	25582.9	16656.7	18905.9	17887.0	25471.4	24191.2	24253.7
1088	2	426.235	850.456	4.21	YQISVNK	sp|P02675	0.002823	11403.2	11259.9	9325.0	7739.7	8003.7	6998.0	9991.5	8191.7	8199.2
1201	2	516.786	1031.558	4.13	IRPFFPQQ	sp|P02675	0.000804	27739.0	28113.0	27181.3	17448.7	21848.7	18282.9	28222.2	25029.4	30582.1
1256	3	651.007	1949.999	18.90	EEAPSLRPAPPPISGGGY	sp|P02675	7.53E-05	41422.5	54789.0	50552.4	30431.8	28455.2	28689.6	30983.5	28947.0	32825.8
1395	5	847.221	4231.068	1.46	QVKDNENVVNEYSSELEI	sp|P02675	2.33E-07	59984.7	57696.5	58933.7	33379.8	30912.3	35146.9	104615.5	93704.2	87616.3
1418	4	669.835	2675.310	11.87	TPCTVSCNIPVVSGKECE	sp|P02675	2.37E-06	25805.4	30788.6	23606.5	15212.1	19208.2	15758.9	61974.5	57117.4	75242.5
1429	3	828.739	2483.195	14.64	NYCGLPGEYWLGNDKIS	sp|P02675	5.97E-06	20982.7	20740.0	25034.3	12757.9	12334.7	17136.8	54753.0	53859.2	51137.8
1559	4	559.556	2234.195	6.55	KREEAPSLRPAPPPISGG	sp|P02675	0.007233	24170.8	34109.8	23446.4	14775.3	20831.7	15903.0	25001.3	24390.8	30370.0
1675	3	709.701	2126.080	11.24	HQLYIDETVNSNIPTNLR	sp|P02675	6.30E-06	30331.5	27654.2	32911.9	22837.2	23977.1	23746.1	15168.6	16813.8	17284.3
1772	2	354.672	707.329	1.45	WDPYK	sp|P02675	2.99E-06	4415.4	4865.5	4520.6	3254.5	3551.6	2963.9	6334.6	6375.5	6357.6
1822	4	585.049	2336.168	12.57	KGGETSEMYLIQPDSSVK	sp|P02675	0.031306	18928.0	22091.4	17364.0	15368.8	21782.2	13377.6	24787.3	23756.2	26425.5
1932	3	370.185	1107.532	7.54	QDGSVDFGRK	sp|P02675	2.21E-07	3990.5	3793.8	4476.6	3065.7	2771.5	2905.0	6850.7	7017.7	7115.7
2123	4	597.050	2384.173	12.80	KGGETSEMYLIQPDSSVK	sp|P02675	0.000145	11541.6	15440.5	11642.9	12604.8	13379.7	12332.0	23782.2	21343.6	26863.1
2275	5	447.846	2234.194	1.43	KREEAPSLRPAPPPISGG	sp|P02675	1.46E-06	11569.6	12339.4	10740.0	7591.2	7398.6	6780.7	13640.8	13754.7	14951.5
2408	3	359.519	1075.535	1.37	ECEEIIRK	sp|P02675	0.008533	3712.8	3516.4	3954.2	2849.3	2839.1	3069.7	3463.1	3219.4	2983.6
2602	3	513.897	1538.670	8.93	LESDVSAQMEYCR	sp|P02675	0.004055	9707.4	9954.3	9358.1	6175.1	6840.0	6517.8	9178.4	8308.5	7182.4
2644	4	1021.760	4083.012	10.61	KAPDAGGCLHADPDLGV	sp|P02675	0.008707	32722.4	37120.7	40803.0	27536.7	30062.3	33533.1	35685.4	32762.2	29344.7
2689	2	490.705	979.395	10.13	CHAANPNGR	sp|P02675	1.49E-05	6862.9	5083.4	6361.2	6108.0	5368.2	6083.1	10483.3	10098.2	10583.5
2815	2	834.862	1667.710	14.06	YYWGGQYTWDMAK	sp|P02675	0.000363	9276.6	9100.3	10602.1	10886.0	10189.0	9479.9	14916.6	17415.0	12450.9
4253	3	515.571	1543.691	3.20	HGTDDGVVWMNWK	sp|P02675	0.000375	2971.9	3113.9	3160.2	3514.6	2993.0	2819.4	5092.3	4408.6	4497.6
4805	4	944.206	3772.795	15.96	QGFGNVATNTDGKNYCG	sp|P02675	3.39E-07	2539.3	2841.6	2326.1	916.5	380.6	898.8	37580.0	35973.0	30298.7
4807	4	555.534	2218.106	10.09	MGPTELLIEMEDWKGDK	sp|P02675	1.63E-07	3357.9	2908.9	2982.6	3340.5	2783.9	3118.7	9686.7	11645.5	10810.6

4953	2	490.213	978.411	9.87	CHAANPNGR	sp|P02675	0.000225	6532.1	5686.2	6830.2	4648.2	3756.9	4211.1	7257.2	7367.1	8004.4
6022	3	1025.802	3074.385	20.52	QGFGNVATNTDGKNYCG	sp|P02675	9.56E-08	5522.7	4821.6	5386.4	2282.5	1731.7	2573.7	23630.6	25311.2	21201.3
8565	2	419.696	837.378	1.36	GSWYSMR	sp|P02675	0.00094	1838.9	1475.1	1829.2	1353.5	989.6	1257.8	2188.5	2302.2	2338.9
12723	4	792.628	3166.484	15.27	NSVDELNNNVEAVSQTSS	sp|P02675	0.002558	2613.7	2563.3	2467.9	2352.9	1847.7	2664.4	2755.1	2596.7	2455.3
14365	2	490.704	979.394	8.66	CHAANPNGR	sp|P02675	0.000526	3046.6	2576.5	3213.1	2628.8	1883.3	2535.6	5117.0	5123.9	4454.6
260	5	743.355	3711.740	5.60	VAQLEAQCQEPCKDTVQ	sp|P02679	0.000233	219510.3	241050.9	193657.0	150670.5	131176.1	162597.5	256383.1	238172.1	280237.1
296	3	736.353	2206.036	14.90	EGFGHLSPTGTTEFWLGI	sp|P02679	0.000276	161323.8	137146.1	136351.7	113979.5	98140.1	104294.9	129487.6	141259.3	146772.1
382	3	806.366	2416.077	18.11	FGSYCPTTCGIADFLSTY(	sp|P02679	0.00861	149517.5	128276.0	124981.2	118109.4	95194.3	126963.8	177014.3	165618.1	140229.8
393	3	431.924	1292.751	13.06	QSGLYFIKPLK	sp|P02679	0.003982	39462.7	52250.1	36082.4	27370.9	28077.4	27657.7	38839.8	41136.0	47088.3
430	4	692.839	2767.328	11.59	VAQLEAQCQEPCKDTVQ	sp|P02679	0.001335	108204.0	89999.1	98008.2	103747.7	98222.8	95890.1	73705.8	68855.3	78656.8
443	3	497.920	1490.740	13.06	YEASILTHDSSIR	sp|P02679	0.006965	46179.7	54982.8	42787.9	38624.8	50029.2	40291.9	32123.8	27575.2	34765.1
445	3	345.514	1033.521	8.79	VGPEADKYR	sp|P02679	7.65E-06	28947.1	25436.5	26724.9	22416.0	19980.3	22405.6	34712.2	34439.7	34417.3
473	3	561.659	1681.955	10.63	IHLISTQSAIPYALR	sp|P02679	0.018645	51041.5	62748.2	57552.1	44846.1	62678.8	46034.9	41123.3	37795.5	32666.0
496	4	387.209	1544.809	7.58	LTIGEGQQHHLGGAK	sp|P02679	1.30E-05	58622.9	52641.2	55738.2	48756.4	43032.7	41081.1	54639.3	52874.9	51932.6
508	2	757.368	1512.722	15.11	YLQEIYNSNNQK	sp|P02679	0.002135	59801.9	79517.2	66908.6	43263.1	59643.7	48968.2	80819.6	85325.8	94648.0
555	2	559.268	1116.522	7.24	VELEDWNGR	sp|P02679	0.007753	36278.6	36963.1	34470.1	25825.0	32016.2	25396.3	28305.4	26044.6	30767.4
605	2	597.747	1193.480	6.57	DNCCILDER	sp|P02679	0.000211	33017.4	34460.0	31648.5	22465.5	23472.4	19996.9	39116.6	39236.9	52906.4
633	2	426.225	850.435	1.33	NWIQYK	sp|P02679	0.000746	19900.1	21618.8	18983.6	15832.4	17624.5	15855.3	21734.1	21229.4	24251.7
881	4	618.822	2471.257	4.34	AIQLTYNPDESSKPNMID/	sp|P02679	0.003267	45170.9	59170.7	42329.9	35921.6	39332.6	29066.2	67567.9	58097.7	56739.2
911	3	362.842	1085.505	4.12	TSTADYAMFK	sp|P02679	0.000173	12163.3	13533.5	12973.4	8862.0	8793.2	8583.7	12924.4	11726.7	11006.3
959	2	638.870	1275.725	7.89	QSGLYFIKPLK	sp|P02679	0.006588	19107.6	19769.2	23681.7	18235.8	16993.7	19276.9	27584.3	25874.2	22216.4
1213	4	503.979	2011.888	4.38	CHAGHLNGVYYQGGTYS	sp|P02679	0.00443	19631.9	24952.8	20670.3	15942.8	15303.0	15603.7	20396.3	23793.0	30723.1
1290	3	505.248	1512.722	5.63	YLQEIYNSNNQK	sp|P02679	2.06E-06	13973.8	15012.3	16188.8	10716.3	10004.3	10947.0	18853.8	18881.3	19411.0
1396	3	336.851	1007.531	7.39	LDGSVDFKK	sp|P02679	0.000439	6302.6	5878.4	5530.7	4677.8	4790.3	4644.1	6325.4	6594.7	6911.9
1528	2	559.761	1117.507	6.36	VELEDWNGR	sp|P02679	0.000313	24274.3	23823.4	22574.1	19597.5	23075.2	19286.3	15213.9	12188.2	13845.1
1588	2	567.762	1133.509	8.55	TSTADYAMFK	sp|P02679	0.000484	9208.2	8902.0	9436.0	8861.6	11488.9	9193.9	13572.3	12741.1	14534.8
1798	3	840.761	2519.262	18.17	AIQLTYNPDESSKPNMID/	sp|P02679	0.000359	28601.1	24490.9	29149.9	27564.7	35280.8	28171.1	58360.1	54370.3	47145.1
1829	3	515.943	1544.808	7.45	LTIGEGQQHHLGGAK	sp|P02679	0.035905	6537.1	5511.7	7192.2	4584.6	4840.1	6472.8	7555.8	6239.4	8180.5
1884	2	482.214	962.414	6.37	DCQDIANK	sp|P02679	0.002376	20311.6	17188.4	20490.0	16775.6	13542.6	16633.2	10870.8	10846.8	11482.4
1931	2	1209.045	2416.074	19.90	FGSYCPTTCGIADFLSTY(	sp|P02679	0.030078	54242.0	44872.3	46926.9	41578.3	35731.8	45433.7	69420.1	60449.3	48968.6
1941	3	888.082	2661.223	16.94	ANQQFLVYCEIDGSGNG\	sp|P02679	0.00546	36242.1	33755.4	35078.7	35395.3	29170.9	36771.5	34384.4	33165.0	30233.2
2250	2	440.724	879.434	5.93	LDGSVDFK	sp|P02679	0.000136	6244.2	6956.0	6365.6	2966.5	3797.8	3190.8	4935.0	5171.3	4523.6
2945	2	517.768	1033.521	6.46	VGPEADKYR	sp|P02679	1.61E-05	6362.1	6093.7	5989.8	5503.5	5465.5	5569.5	7138.9	7026.4	7491.6
3573	4	696.124	2780.465	11.70	IHLISTQSAIPYALRVELEI	sp|P02679	2.70E-08	5891.4	5113.4	4667.6	2644.7	2400.2	1804.8	27723.9	26840.2	27115.0

3894	2	757.861	1513.707	14.06	YLQEIYNSNNQK	sp|P02679	0.000425	6262.1	6782.6	6920.6	5521.5	7487.0	6710.0	10187.3	8442.2	9084.9
4512	4	696.370	2781.450	7.68	IHLISTQSAIPYALRVELEI	sp|P02679	2.90E-08	3871.4	3549.8	4216.8	2654.7	2070.5	2071.1	15722.5	17486.0	16295.7
7541	4	592.300	2365.172	7.47	MLEEIMKYEASILTHDSSI	sp|P02679	1.01E-07	1750.2	1584.9	1903.8	1758.5	1437.9	1372.1	7171.3	7325.7	6179.3
520	3	604.636	1810.887	14.64	IVLGQEQDSYGGKFDR	sp|P02743	0.000305	47082.4	45984.8	42817.3	45503.9	47409.4	47299.2	58829.3	52939.1	54589.1
682	2	382.727	763.439	3.27	VFVFPR	sp|P02743	0.002626	12507.5	12887.1	12939.2	13219.1	11447.1	12015.4	14979.4	16005.5	17290.9
1066	2	578.805	1155.595	14.02	VGEYSLYIGR	sp|P02743	0.034335	16117.6	19416.5	17430.0	20049.4	21360.6	19290.4	12240.2	12033.3	18003.9
1275	2	643.899	1285.783	10.96	GYVIIKPLVWV	sp|P02743	0.003842	19368.8	17223.5	19243.1	24793.4	21072.3	24857.0	17333.7	17213.3	14401.0
1348	2	703.840	1405.665	16.06	AYSLFSYNTQGR	sp|P02743	5.08E-05	22143.4	28538.4	29583.2	29812.0	32136.3	31982.1	13135.2	15302.3	13245.0
1444	2	406.201	810.388	3.64	AYSDLSR	sp|P02743	0.000438	8383.6	7352.9	7639.0	8778.7	8784.1	8048.3	6558.1	6251.2	6233.4
2688	2	621.878	1241.741	9.24	LSPIYNLVPVK	sp|P02748	0.000106	5906.3	5237.0	5561.7	9615.4	8223.0	8205.5	10235.8	11186.7	9273.4
5564	2	728.361	1454.707	10.54	AIEDYINEFSVR	sp|P02748	0.001819	3416.5	2368.4	3198.8	5094.0	3740.4	4764.2	6450.8	6637.6	5243.6
6255	3	930.485	2788.432	21.99	GTVIDVTDFVNWASSIND	sp|P02748	0.001767	4582.2	3787.1	5707.1	10632.3	8340.3	8562.5	7077.1	8117.3	7076.5
19937	4	698.116	2788.434	16.27	GTVIDVTDFVNWASSIND	sp|P02748	0.014429	642.6	462.0	698.2	1269.2	815.1	1113.5	930.2	983.4	873.0
1370	2	495.281	988.547	6.50	VAAGAFQGLR	sp|P02750	0.001009	7054.2	7451.6	6016.6	8062.2	10712.9	7796.1	12373.9	12198.4	15812.4
1407	2	358.719	715.424	1.47	VLDLTR	sp|P02750	0.000283	3739.2	4571.4	3863.9	3916.3	5726.6	4189.7	8589.3	8565.3	10722.0
1494	2	406.736	811.457	1.43	GPLQLER	sp|P02750	1.60E-06	4320.5	5109.3	4645.8	4817.1	5905.2	5388.3	10878.5	10870.3	11129.3
1617	3	384.876	1151.606	7.54	ALGHLDLSGNR	sp|P02750	0.002238	4236.2	5579.5	4643.4	4953.0	6984.7	5188.1	9176.6	8603.0	11903.2
1635	4	740.903	2959.581	15.78	LQELHLSSNGLESLSPEF	sp|P02750	0.00136	24859.1	17620.0	23546.4	29062.0	21510.0	30832.3	48052.9	47069.7	42469.5
2095	2	450.780	899.546	7.56	GQTLLAVAK	sp|P02750	0.01911	4755.9	5560.2	4623.4	5106.9	6930.6	4979.7	6985.9	7446.0	7911.4
2296	2	485.257	968.499	3.27	YLFLNGNK	sp|P02750	2.27E-05	3313.9	3576.1	3540.4	4161.3	4816.2	4829.5	8541.2	9068.9	6814.6
2642	3	632.009	1893.004	11.51	ENQLEVLEVSWLHGLK	sp|P02750	3.23E-05	8621.2	8322.7	9951.2	14006.5	13648.3	13956.6	6906.5	6572.7	5462.8
3855	3	829.042	2484.106	20.11	DGFDISGNPWICDQNLSI	sp|P02750	0.019813	11570.7	9033.4	10213.5	13352.5	13207.5	14192.9	10344.4	10729.8	10491.1
4822	3	679.702	2036.083	13.16	TLDLGENQLETLPPDLLR	sp|P02750	0.000155	16884.1	15126.0	17580.8	20111.7	15001.5	19072.3	30342.0	30746.3	29151.1
8338	3	780.779	2339.314	17.38	NALTGLPPGLFQASATLD	sp|P02750	1.12E-06	3728.1	3535.1	3795.8	7453.4	7027.1	8181.6	2335.3	1861.1	1850.6
9616	3	270.821	809.442	2.22	LHLEGNK	sp|P02750	0.001762	793.3	683.4	823.7	924.7	784.6	889.6	1545.9	1538.5	1561.3
10841	4	1054.583	4214.303	9.77	NALTGLPPGLFQASATLD	sp|P02750	1.23E-05	4502.5	3058.4	4198.5	6029.7	4528.4	5584.8	11554.5	13876.4	12744.7
15088	2	1243.059	2484.103	17.90	DGFDISGNPWICDQNLSI	sp|P02750	0.001832	3646.1	2868.1	2805.7	4344.5	3857.0	5019.1	2860.8	2745.0	2750.2
3070	3	551.267	1650.779	7.22	LLCQCLGFGSGHFR	sp|P02751	0.000169	9909.0	13032.8	9590.3	6516.3	6582.9	5887.3	5702.1	5292.1	6963.1
7288	3	643.021	1926.041	13.67	FLATTPNSLLVSWQPPR	sp|P02751	0.000583	6063.6	5254.3	6483.0	4137.5	2918.5	4384.7	3256.0	3429.4	2561.1
8798	3	355.518	1063.532	7.28	VGDTYERPK	sp|P02751	0.00217	2048.7	1805.7	2102.1	1451.7	1258.5	1404.4	960.1	1001.8	1073.9
12687	3	642.689	1925.045	15.11	VTWAPPPSIDLTNFLVR	sp|P02751	8.09E-06	3023.9	2986.7	3210.0	2360.0	1765.1	2068.6	1470.9	1378.6	1265.4
17112	3	842.128	2523.361	22.38	DLEVVAATPTSLLISWDAI	sp|P02751	1.28E-06	2768.2	2153.9	2656.0	2489.1	1882.8	2397.0	501.8	474.6	327.9
3951	2	599.818	1197.621	6.65	YWGVASFLQK	sp|P02753	0.000439	4366.6	3774.7	3901.7	5418.6	4568.4	5220.0	3190.6	3022.1	3445.5
5078	3	434.892	1301.653	6.69	QRQEELCLAR	sp|P02753	2.81E-05	2046.0	2064.7	2155.0	2266.8	2569.3	2356.9	1338.7	1452.9	1311.3

456	2	607.341	1212.667	14.06	TVAACNLPIVR	sp|P02760	0.004089	46023.3	45725.6	40103.3	57503.5	73256.6	54922.3	70980.7	59031.9	83899.4
574	2	704.886	1407.758	13.62	AFIQLWAFDAVK	sp|P02760	0.001086	45516.5	41663.5	45915.7	65116.8	50949.0	70966.4	67925.0	73803.2	70745.9
650	2	854.879	1707.743	15.95	EYCGVPGDGDEELLR	sp|P02760	2.64E-05	73294.9	80763.2	81124.7	101047.5	109226.8	106284.5	129023.0	117419.8	123605.0
2665	2	483.711	965.407	7.27	ECLQTCR	sp|P02760	0.012549	6259.2	5323.1	6491.5	11156.8	7976.9	10187.9	5159.6	5805.4	5139.5
3082	2	836.367	1670.719	12.00	CVLFPYGGCQGNGNK	sp|P02760	4.29E-05	12044.5	14176.0	12087.0	18957.8	20481.0	20238.1	12581.9	13183.1	9906.6
3543	3	470.260	1407.758	8.05	AFIQLWAFDAVK	sp|P02760	0.009431	3783.4	2974.6	3438.8	4699.8	3653.6	5381.0	4730.2	5438.1	5043.0
3590	3	776.015	2325.022	9.60	CVLFPYGGCQGNGNKFY	sp|P02760	0.001888	6780.4	7023.9	8741.9	10111.5	10712.8	7966.4	13912.6	13996.0	13301.2
3843	3	373.503	1117.488	5.72	FYSEKECR	sp|P02760	0.001468	2022.4	1804.4	2217.2	3315.4	2447.0	2935.5	4741.1	5139.7	4426.3
4162	3	649.615	1945.823	11.29	GVCEETSGAYEKTDTDG	sp|P02760	0.000673	8261.6	6090.5	7358.7	9700.1	8293.5	9436.5	5018.9	5333.2	5325.6
4739	4	693.557	2770.197	9.65	CVLFPYGGCQGNGNKFY	sp|P02760	1.75E-05	3309.2	3284.6	2547.6	2770.5	1362.3	3227.3	18893.0	20574.5	24670.6
25	4	438.995	1751.949	7.98	YVGGQEHFAHLLILR	sp|P02763	0.002236	338949.2	440603.6	345982.5	387615.9	547383.6	385780.4	712844.2	679690.8	708016.7
38	3	414.212	1239.616	12.77	SDVVYTDWKK	sp|P02763	5.14E-05	149478.9	184611.1	137319.0	165241.1	205412.9	165564.0	381298.6	336680.7	393014.1
106	3	584.990	1751.949	13.95	YVGGQEHFAHLLILR	sp|P02763	0.006213	168168.9	235726.5	176118.4	182062.8	299665.3	194506.9	401029.3	332045.1	399048.6
108	2	620.815	1239.615	13.87	SDVVYTDWKK	sp|P02763	0.001226	109152.7	156296.7	93506.5	128357.5	193596.5	132416.8	290732.7	282869.5	349370.5
114	2	556.768	1111.521	12.29	SDVVYTDWK	sp|P02763	0.015501	111025.8	133951.5	131712.5	140933.3	181403.9	136462.5	156170.7	148909.4	206345.4
240	2	871.907	1741.800	16.06	EQLGEFYEALDCLR	sp|P02763	1.29E-06	203462.3	201113.4	214324.6	297998.5	254666.1	290407.5	110604.0	123144.7	110876.9
437	3	581.608	1741.802	10.28	EQLGEFYEALDCLR	sp|P02763	3.33E-06	62736.9	59046.5	70098.0	88368.5	77442.9	89490.1	38933.3	39326.5	39265.6
857	5	972.664	4858.286	11.15	TYMLAFDVNDEKNWGLS	sp|P02763	1.06E-05	158672.2	127516.7	165604.2	237093.7	214491.5	236279.0	373364.9	431808.4	434206.0
1024	5	618.306	3086.493	11.28	TYMLAFDVNDEKNWGLS	sp|P02763	9.59E-07	42521.7	44258.9	41534.8	60479.7	57972.6	62061.2	30540.2	26681.0	27175.8
1608	4	858.917	3431.641	12.23	NWGLSVYADKPETTKEQ	sp|P02763	3.10E-05	49622.4	39128.8	43356.8	57575.2	53485.6	56832.0	31522.2	28770.7	34528.1
1871	4	772.630	3086.492	20.52	TYMLAFDVNDEKNWGLS	sp|P02763	5.37E-08	30505.0	32931.2	30049.0	42105.7	42956.6	42903.3	21248.4	21033.3	21618.1
2074	3	570.290	1707.849	8.41	NWGLSVYADKPETTK	sp|P02763	0.012276	12058.3	15334.0	13379.9	16203.9	24096.9	15941.6	13578.0	12389.8	14651.1
5835	3	694.352	2080.035	11.69	EQLGEFYEALDCLRIPK	sp|P02763	2.37E-08	2069.1	1629.8	1776.7	2204.4	1630.0	2181.7	23503.1	25918.4	23227.9
7656	5	963.059	4810.261	13.38	TYMLAFDVNDEKNWGLS	sp|P02763	0.003362	17126.1	11165.9	15347.3	16857.3	11822.4	16121.9	26784.5	29023.2	24472.9
299	2	424.221	846.428	6.09	CNLLAEK	sp|P02765	0.000688	38058.9	35905.4	39975.9	44270.9	41914.3	39808.8	44891.9	45193.3	48702.7
394	2	401.682	801.350	3.48	QYGFCK	sp|P02765	0.023566	25523.2	25967.8	22904.4	25859.8	30496.2	24467.0	30168.3	32669.4	27220.0
455	5	971.892	4854.422	17.97	HTLNQIDEVKVWPQQPS	sp|P02765	0.006463	265200.5	192096.2	253769.6	234592.0	242533.2	304268.1	393923.0	375731.5	345223.6
610	3	1121.855	3362.544	12.95	QPNCDDPETEEAALVAID	sp|P02765	0.000677	232978.8	208218.9	270062.9	296804.8	224400.7	251135.6	144598.2	143425.9	130004.6
718	4	508.257	2028.998	3.08	QLKEHAVEGDCDFQLLK	sp|P02765	0.007089	33861.3	35552.5	31562.0	38947.3	43870.4	36993.1	36221.4	42208.8	36598.2
989	3	554.260	1659.758	13.06	EHAVEGDCDFQLLK	sp|P02765	0.00034	28793.3	36214.9	25689.2	28601.1	30804.8	28432.5	17523.1	15617.2	14900.7
1000	3	671.664	2011.970	13.70	QLKEHAVEGDCDFQLLK	sp|P02765	0.006313	23858.4	31873.4	28059.0	27813.7	30922.3	26595.4	37780.5	39973.1	42018.7
1060	3	860.441	2578.302	16.38	AQLVPLPPSTYVEFTVSG	sp|P02765	0.003878	223873.3	192915.8	208604.8	248452.4	209896.3	240728.3	183455.6	179464.3	166878.9
1697	3	399.547	1195.620	4.97	HTLNQIDEVK	sp|P02765	6.27E-06	6574.1	8170.7	8197.8	8848.9	8214.8	9858.4	3190.1	3716.4	3328.0
1705	1	830.409	829.402	1.44	CNLLAEK	sp|P02765	0.001037	13994.1	17190.0	15168.0	17980.4	23098.8	17947.1	13415.7	14754.6	12115.9

1899	3	1226.611	3676.811	17.69	VWPQQPSGELFEIEIDTLI	sp|P02765	3.69E-06	114797.8	92277.3	108240.9	159773.9	125324.3	139667.5	47405.4	46638.5	42588.6
2076	4	920.211	3676.814	18.90	VWPQQPSGELFEIEIDTLI	sp|P02765	8.94E-06	73200.2	62326.6	75977.6	96014.0	77858.4	83375.5	36127.1	33305.4	29348.4
2218	4	841.644	3362.546	19.18	QPNCDDPETEEAALVAID	sp|P02765	0.000236	41590.6	36282.8	43377.5	50134.1	40415.0	45361.7	25705.8	26126.0	22397.0
2433	2	598.817	1195.620	12.09	HTLNQIDEVK	sp|P02765	7.84E-06	7002.6	9971.9	8332.6	9465.2	11373.0	11312.6	2925.1	3424.9	2754.7
3156	4	611.564	2442.225	11.88	QLKEHAVEGDCDFQLLKL	sp|P02765	1.21E-05	3558.3	5675.6	2941.2	2289.7	3672.2	2057.9	23833.9	25691.1	36344.0
3310	6	810.079	4854.427	14.18	HTLNQIDEVKVWPQQPS(	sp|P02765	0.002317	21915.0	16766.3	21766.7	21425.6	21153.2	25751.9	35392.9	33514.6	29881.5
3505	3	1093.888	3278.642	7.70	AQLVPLPPSTYVEFTVSG	sp|P02765	2.53E-11	3824.4	3619.1	3715.7	1575.9	1620.2	2127.4	120235.0	115493.6	109604.5
4293	2	360.183	718.351	1.39	EATEAAK	sp|P02765	0.011823	12572.0	10977.6	13212.7	18097.3	14932.4	18256.3	15135.2	15570.2	12065.6
4341	3	489.213	1464.617	15.80	CDSSPDSAEDVRK	sp|P02765	0.00013	3420.0	2989.6	3752.1	3714.0	3209.9	3375.9	2489.0	2292.2	2301.2
4879	4	607.307	2425.199	14.83	QLKEHAVEGDCDFQLLKL	sp|P02765	3.84E-09	2589.1	2959.7	2570.2	1505.1	1563.8	1721.5	19107.4	17854.8	14897.5
7637	4	820.669	3278.645	15.65	AQLVPLPPSTYVEFTVSG	sp|P02765	3.68E-09	782.1	606.9	659.6	146.1	242.8	124.3	28447.4	26580.6	23662.8
22298	3	1122.184	3363.530	10.86	QPNCDDPETEEAALVAID	sp|P02765	2.89E-06	2607.3	2251.7	2732.9	3122.6	3160.2	3477.1	1362.4	1271.5	1117.7
22678	3	1122.183	3363.528	14.90	QPNCDDPETEEAALVAID	sp|P02765	1.15E-05	3426.9	3107.4	3535.3	5424.7	4158.5	5094.0	1704.5	1226.3	1445.6
378	2	697.816	1393.617	17.11	AADDTWEPFASGK	sp|P02766	0.010034	73066.9	83548.1	72362.9	76429.8	75457.1	66380.4	63612.8	60968.5	54160.0
389	4	785.886	3139.515	17.52	TSESGELHGLTTEEEFVE	sp|P02766	0.009326	217977.4	177252.6	210961.4	209134.6	181836.8	201669.6	161626.7	149634.5	143550.9
554	3	456.259	1365.755	12.21	GSPAINVAVHVFR	sp|P02766	0.000334	40606.1	44245.0	39630.9	40805.6	54665.2	40850.1	29222.3	25493.6	31553.1
1056	4	613.558	2450.203	3.76	ALGISPFHEHAEVVFTANI	sp|P02766	0.00101	43909.1	51516.3	46011.8	44799.6	60780.6	51215.8	31327.6	29029.1	41134.9
2253	3	819.056	2454.148	16.17	TSESGELHGLTTEEEFVE	sp|P02766	5.30E-07	25029.9	24338.6	21868.6	26703.2	23159.9	28700.1	6684.2	8009.9	7228.6
3299	2	1245.145	2488.276	16.91	YTIAALLSPYSYSTTAVVT	sp|P02766	0.028959	21839.8	20316.1	25409.7	28042.7	34529.8	24353.2	21737.7	20338.2	18324.2
232	2	575.312	1148.609	9.90	LVNEVTEFAK	sp|P02768	0.00042	113791.5	100054.7	84639.2	74039.9	88642.2	75717.5	48874.9	44163.0	52784.1
268	3	682.371	2044.091	15.90	VFDEFKPLVEEPQNLIK	sp|P02768	0.01082	180655.2	111191.6	152403.3	117155.7	100094.9	143624.3	94734.7	87129.2	68249.3
276	2	722.326	1442.637	16.06	YICENQDSISSK	sp|P02768	6.08E-06	128473.8	145990.7	126238.2	90227.5	94730.5	88363.8	87542.8	85831.3	79871.1
280	2	395.240	788.466	4.41	LVTDLTK	sp|P02768	8.60E-06	32805.2	35214.3	31675.4	24750.5	30906.5	23578.5	18301.8	18892.5	18264.5
329	2	464.251	926.488	5.95	YLYEIAR	sp|P02768	0.007481	39355.6	50681.8	50640.4	31163.0	43745.9	33873.3	31282.1	26732.6	32861.1
337	3	358.853	1073.537	3.63	LDELRDEGK	sp|P02768	3.57E-05	34835.7	27739.1	32623.8	24723.2	21911.3	23801.2	18967.4	15576.7	16958.2
343	2	686.288	1370.561	15.06	AAFTECCQAADK	sp|P02768	1.90E-08	117041.7	118720.2	107680.0	76968.1	86400.9	82507.0	36124.1	38656.7	39603.2
361	2	569.753	1137.492	7.42	CCTESLVNR	sp|P02768	1.41E-07	56318.5	58087.3	52677.7	42873.2	41102.0	38625.6	23355.4	22928.7	23481.0
392	2	749.794	1497.573	17.06	TCVADESAENCDK	sp|P02768	0.000127	101497.8	88008.3	113806.0	77620.7	65295.0	82570.0	46858.0	46672.1	38971.0
401	3	547.318	1638.934	13.90	KVPQVSTPTLVEVSR	sp|P02768	0.000249	75894.1	96789.4	88392.5	64215.8	69198.5	54772.8	47584.4	47287.7	53482.5
441	2	480.786	959.557	5.60	FQNALLVR	sp|P02768	3.33E-07	44863.3	55122.4	45708.9	34628.5	35339.5	29575.8	11500.9	12170.1	13111.5
454	2	500.807	999.599	11.90	QTALVELVK	sp|P02768	0.01454	37082.1	52543.8	35376.0	23078.8	33819.7	22671.0	22148.4	22205.7	25722.3
458	2	492.748	983.482	7.51	TYETTLEK	sp|P02768	8.56E-05	44000.1	37081.2	40084.9	31430.8	36140.3	27209.2	17991.0	21217.5	19303.4
459	4	407.688	1626.722	6.89	ADDKETCFAEEGKK	sp|P02768	1.45E-06	43960.3	41376.7	41896.6	30234.6	26395.4	29223.2	27489.0	27375.6	28865.0
491	3	409.541	1225.601	6.16	FKDLGEENFK	sp|P02768	0.001615	30558.4	42619.0	31865.1	21920.8	28078.0	22537.2	18848.1	19138.7	20255.1

532	2	507.304	1012.594	15.27	LVAASQAALGL	sp|P02768	0.002073	33352.6	38135.8	37128.5	24390.8	21674.7	25875.7	23195.5	27394.9	21646.9
535	3	518.205	1551.593	13.06	CCAAADPHECYAK	sp|P02768	0.001172	57185.7	46738.8	60236.9	38971.8	37838.1	40970.4	32332.1	34577.3	32377.0
569	5	517.830	2584.116	5.82	VHTECCHGDLLECADDR	sp|P02768	0.011962	52641.8	71334.1	57077.9	46713.0	54308.1	38927.2	38725.5	36009.0	45573.7
579	4	647.036	2584.115	10.76	VHTECCHGDLLECADDR	sp|P02768	0.002704	73361.4	115463.3	78766.6	57305.9	92452.4	52207.0	33179.5	28923.3	38692.2
603	2	440.724	879.434	6.32	AEFAEVSK	sp|P02768	0.000208	21280.0	20759.5	19302.3	15613.0	17606.6	13378.2	9804.7	10881.3	11496.4
732	3	637.650	1909.928	10.49	RPCFSALEVDETYVPK	sp|P02768	8.61E-09	86327.7	96948.2	100669.9	63897.4	75207.9	69826.0	18690.6	19075.6	19006.7
764	4	475.505	1897.990	7.84	RHPYFYAPELLFFAK	sp|P02768	0.001749	37131.1	32989.6	40944.0	26811.7	21040.8	27217.6	24704.7	24174.0	21541.7
820	3	432.216	1293.625	5.06	AVMDDFAAFVEK	sp|P02768	6.35E-07	21152.9	22243.6	20834.4	12304.9	12752.3	12296.0	12047.2	11369.0	12339.0
887	3	339.851	1016.532	5.76	SLHTLFGDK	sp|P02768	8.87E-06	14316.0	20339.6	14944.6	11061.4	14678.8	10371.3	3330.0	3154.5	3901.2
900	3	489.953	1466.837	9.96	RHPDYSVVLLLR	sp|P02768	0.03971	25250.5	51535.8	29060.1	16405.1	34427.9	21105.8	14434.2	13205.0	16554.2
909	2	386.724	771.432	3.51	AACLLPK	sp|P02768	1.16E-07	24011.5	25574.3	24277.5	16804.4	17287.1	16320.4	10522.5	10392.4	11653.1
928	3	543.248	1626.722	13.99	ADDKETCFAEEGKK	sp|P02768	4.64E-08	33128.8	31693.5	31487.3	22040.3	20617.8	22530.4	18882.8	17818.6	18477.9
984	5	528.052	2635.223	4.43	QEPERNECFLQHKDDNP	sp|P02768	0.000136	70570.8	89736.7	68350.0	46169.7	56545.3	47412.1	37795.6	39546.1	38792.4
1003	4	483.766	1931.034	7.08	SLHTLFGDKLCTVATLR	sp|P02768	0.001017	27120.2	35625.9	27077.7	16062.4	25768.3	17113.3	13163.6	12185.0	9934.2
1021	2	829.381	1656.748	17.06	QNCELFEQLGEYK	sp|P02768	1.05E-07	67427.6	69838.8	52849.6	42109.8	42826.0	44526.3	13414.8	11223.1	11944.0
1073	4	623.328	2489.283	17.11	ALVLIAFAQYLQQCPFEDI	sp|P02768	0.000169	42171.8	49991.3	42452.4	38061.0	28986.8	35178.2	24087.4	22897.0	19313.5
1145	4	637.048	2544.163	7.69	EFNAETFTFHADICTLSEK	sp|P02768	9.99E-07	44919.3	49985.3	47783.6	32400.2	38132.3	36173.3	15653.9	16493.6	12890.3
1160	3	516.271	1545.792	5.84	LKECCEKPLLEK	sp|P02768	0.000477	27731.7	28053.9	26361.4	20882.6	21110.5	17713.7	22801.3	20849.1	15694.9
1220	4	367.717	1466.837	3.10	RHPDYSVVLLLR	sp|P02768	0.014447	14183.7	20763.6	15273.7	10756.4	13544.2	9704.2	8008.4	8538.2	10103.0
1298	3	633.670	1897.990	11.06	RHPYFYAPELLFFAK	sp|P02768	0.002547	31831.5	28469.6	35535.6	22269.5	25770.8	23368.2	21141.0	24936.3	19663.1
1330	3	830.768	2489.281	21.90	ALVLIAFAQYLQQCPFEDI	sp|P02768	0.00058	50436.9	66711.9	50509.7	49303.1	36545.2	46018.5	28883.1	27776.5	23242.9
1371	2	768.290	1534.566	13.12	CCAAADPHECYAK	sp|P02768	0.000798	31704.3	37928.3	33420.8	26760.4	35530.5	24042.7	17421.6	14328.7	17704.4
1372	3	867.105	2598.294	21.64	QNCELFEQLGEYKFQNAI	sp|P02768	1.62E-06	36543.3	28706.1	34521.9	19358.8	15180.9	15943.8	43805.8	42024.7	36427.4
1400	5	683.126	3410.596	10.07	TCVADESAENCDKSLHTL	sp|P02768	2.87E-08	22683.9	31269.7	24686.8	9304.6	9446.4	8716.8	56284.4	60680.9	50877.0
1557	4	655.557	2618.197	11.73	QEPERNECFLQHKDDNP	sp|P02768	5.04E-05	37279.8	39472.7	34804.0	28832.6	35625.2	31253.2	18826.0	16210.3	19715.8
1618	2	537.776	1073.537	6.07	LDELRDEGK	sp|P02768	4.02E-06	13290.0	10915.7	11885.4	8624.3	9147.4	8624.5	5748.7	4726.8	5281.2
1644	2	671.822	1341.630	14.06	AVMDDFAAFVEK	sp|P02768	0.010765	14954.9	12971.5	13600.2	14781.3	9153.6	12870.8	11441.6	10464.4	10885.0
1784	3	553.257	1656.750	9.78	QNCELFEQLGEYK	sp|P02768	9.59E-08	20555.7	20136.7	17519.0	12976.0	11144.4	12837.5	4599.8	3989.6	4230.2
2107	4	659.813	2635.223	11.39	QEPERNECFLQHKDDNP	sp|P02768	0.008344	37019.9	56187.0	33231.1	23598.7	37023.6	25269.2	18361.1	21036.1	22118.7
2337	3	481.886	1442.637	6.92	YICENQDSISSK	sp|P02768	5.24E-05	6878.2	8445.5	7995.8	5566.2	5224.5	5370.3	5884.6	5068.1	5626.8
2384	3	840.077	2517.210	21.90	MPCAEDYLSVVLNQLCVL	sp|P02768	2.58E-05	31929.6	25137.7	26425.0	24732.5	21589.2	27781.5	13565.8	13046.3	13047.4
2509	3	709.001	2123.982	18.70	AAFTECCQAADKAACLLF	sp|P02768	1.59E-07	18518.7	19881.1	18635.6	8289.2	9792.5	8487.6	41545.8	42519.8	58847.8
2592	6	740.354	4436.081	7.12	RPCFSALEVDETYVPKEF	sp|P02768	7.04E-06	25556.4	17738.3	25127.3	11938.2	11779.4	13278.5	31349.4	35416.5	30361.9
2726	3	581.637	1741.890	1.88	HPYFYAPELLFFAK	sp|P02768	1.87E-06	15153.8	14905.9	16029.5	9339.1	8007.9	9599.7	8082.2	8587.5	7946.5

2910	3	754.013	2259.017	15.95	EFNAETFTFHADICTLSEK	sp|P02768	3.54E-08	26327.9	28177.8	21950.5	22951.2	19707.8	21752.7	3525.8	3359.6	2649.7
2917	3	644.685	1931.033	12.70	SLHTLFGDKLCTVATLR	sp|P02768	0.004849	11315.3	17345.1	10756.5	7202.8	12529.8	6844.0	5361.5	4942.6	3340.3
3083	3	435.878	1304.612	5.75	ECCEKPLLEK	sp|P02768	7.61E-07	5166.9	5013.2	5044.4	3592.0	3393.0	3396.0	2680.7	2221.9	2691.3
3414	2	492.293	982.572	6.52	QTALVELVK	sp|P02768	0.002608	3025.2	2930.0	3422.3	2578.8	2000.3	2783.9	2135.7	2028.3	1981.2
3681	5	888.223	4436.077	17.49	RPCFSALEVDETYVPKEF	sp|P02768	1.83E-06	22861.4	15873.4	19214.1	8639.8	9811.8	9774.8	28847.8	31516.0	24840.7
4405	3	992.121	2973.340	19.78	SHCIAEVENDEMPADLPS	sp|P02768	4.55E-05	29721.3	27799.1	28704.5	30707.0	28420.2	29833.1	14663.3	13667.9	9641.2
4938	4	380.450	1517.771	2.20	LDELRDEGKASSAK	sp|P02768	2.83E-12	174.4	194.0	161.9	102.8	101.6	100.4	5942.7	6408.3	7260.2
4939	2	776.804	1551.593	14.12	CCAAADPHECYAK	sp|P02768	5.70E-05	8326.8	7461.7	7875.8	5647.1	7088.9	6036.3	3404.8	3294.5	3396.8
5025	5	740.382	3696.872	17.56	FKDLGEENFKALVLIAFAC	sp|P02768	3.94E-05	13332.0	11110.2	12360.6	5714.0	4954.8	6954.4	9526.0	7414.7	7233.9
5985	2	812.398	1622.782	14.06	DVFLGMFLYEYAR	sp|P02768	0.000118	10455.8	8633.3	10340.0	11949.2	8354.3	10048.2	4638.9	4531.2	4368.2
6552	3	541.935	1622.784	11.32	DVFLGMFLYEYAR	sp|P02768	9.26E-05	4640.8	3689.5	4806.6	5529.3	3568.8	4467.8	1754.7	1641.6	1549.2
11496	3	861.430	2581.267	15.64	QNCELFEQLGEYKFQNAI	sp|P02768	5.80E-05	3486.4	2675.8	3345.6	2290.1	1866.3	1799.3	2929.6	3003.9	2814.3
13368	4	905.977	3619.880	8.12	ALVLIAFAQYLQQCPFEDI	sp|P02768	4.61E-08	3210.7	2913.8	2746.3	2053.8	1921.1	2392.9	14611.7	13960.8	11657.9
14003	5	925.882	4624.373	14.38	VFDEFKPLVEEPQNLIKQI	sp|P02768	3.21E-07	777.6	437.7	865.4	476.5	535.7	436.3	11944.4	9787.0	9614.4
9653	3	936.187	2805.541	11.20	LAQKVPTADLEDVLPLAE	sp|P02774	7.65E-05	6198.5	5100.7	6943.7	4144.7	2735.4	3435.2	12172.3	12553.8	10986.3
15241	3	789.430	2365.267	12.43	VPTADLEDVLPLAEDITNI	sp|P02774	0.000177	3177.8	3299.2	2615.0	2259.9	1845.4	1885.0	1602.5	1365.6	1554.1
4483	3	606.607	1816.800	10.23	EGTCPEAPTDECKPVK	sp|P02787	1.14E-06	7209.4	8576.4	7968.1	5980.9	6128.8	6145.0	3686.5	3831.5	3314.0
5982	3	724.372	2170.093	17.67	SAGWNIPIGLLYCDLPEPI	sp|P02787	7.15E-06	8269.2	7493.6	7724.4	6527.0	5662.8	6521.1	4702.0	4918.5	4781.9
73	2	610.808	1219.601	8.17	NFPSPVDAAFR	sp|P02790	0.002302	227562.0	191195.0	223144.8	286695.8	356698.4	316420.6	332729.9	371257.0	299196.7
87	2	748.344	1494.673	11.16	YYCFQGNQFLR	sp|P02790	0.001584	227645.5	190003.0	228215.0	299975.5	361992.6	330297.0	345466.5	391318.8	309994.8
172	3	381.549	1141.627	9.07	QGHNSVFLIK	sp|P02790	0.02705	63738.5	82864.2	68252.9	90101.8	113973.9	86323.9	95323.1	92318.3	112484.5
196	3	377.223	1128.647	1.45	RLWWLDLK	sp|P02790	0.03283	46798.5	35314.2	42841.5	58797.8	50089.9	61051.9	48176.8	52011.7	50111.9
209	4	783.617	3130.440	22.83	CSPHLVLSALTSDNHGAT	sp|P02790	0.004987	301753.7	242150.9	260584.1	426127.8	340732.0	436527.9	338420.2	339243.9	336970.3
230	3	788.728	2363.161	19.90	LLQDEFPGIPSPLDAAVE(	sp|P02790	7.46E-07	299075.1	271679.5	259850.3	475513.6	383165.1	407135.9	129932.5	134575.2	116220.8
248	3	499.232	1494.675	6.00	YYCFQGNQFLR	sp|P02790	0.001271	56113.8	44819.3	57397.1	72465.3	74014.9	74920.1	79103.2	99742.6	81951.3
302	3	613.301	1836.883	18.90	SGAQATWTELPWPHEK	sp|P02790	4.16E-06	195540.7	171190.1	190271.7	279098.3	336589.7	299078.0	110924.7	113282.5	94827.3
351	2	571.299	1140.584	14.06	GGYTLVSGYPK	sp|P02790	2.53E-06	105080.8	107504.0	111330.3	164754.5	190927.5	162760.2	81697.7	74952.0	87049.6
358	4	945.710	3778.813	18.83	SLGPNSCSANGPGLYLIH	sp|P02790	3.22E-08	132023.6	134703.0	164654.1	146595.9	135565.4	145757.2	508952.1	511255.5	502922.6
388	2	563.307	1124.599	6.30	QGHNSVFLIK	sp|P02790	0.000931	32290.8	42749.0	38312.3	43643.5	49606.0	48963.2	59484.4	60810.6	69590.7
404	2	565.331	1128.647	1.43	RLWWLDLK	sp|P02790	0.002938	40099.3	35645.5	34737.6	57245.1	47956.9	60763.3	40037.6	43699.7	40492.8
544	4	821.386	3281.516	17.90	SLGPNSCSANGPGLYLIH	sp|P02790	0.044193	107938.3	100694.2	115452.7	146959.7	114227.9	139845.2	105434.6	103838.4	75897.3
653	3	365.496	1093.467	2.17	DYFMPCPGR	sp|P02790	0.003039	23700.5	28489.0	23383.5	30499.6	31500.5	29062.8	31530.0	32853.4	34709.9
897	2	511.737	1021.459	7.20	VDGALCMEK	sp|P02790	5.37E-07	11544.8	12243.6	12200.6	21414.9	24496.6	21303.0	9802.0	9536.4	9015.9
990	4	362.201	1444.774	6.55	GDKVWVYPPEKK	sp|P02790	4.49E-05	37909.0	42738.2	34273.7	49384.8	54748.6	47637.6	65871.7	68592.7	67899.5

1039	4	805.625	3218.470	10.47	DGWHSWPIAHQWPQGP	sp|P02790	3.18E-07	102784.3	80169.2	100718.9	183855.0	151254.4	180000.8	39523.5	39163.1	35069.3
1101	2	571.743	1141.471	9.50	DYFMPCPGR	sp|P02790	9.79E-06	11850.3	12941.4	12192.7	21398.3	21521.4	20683.4	23405.2	24145.1	29721.1
1164	3	591.339	1770.996	15.11	LYLVQGTQVYVFLTK	sp|P02790	1.37E-08	40867.8	33827.0	39598.3	69892.8	60202.3	67676.3	13488.7	12606.1	12582.4
1331	4	421.979	1683.888	1.35	FDPVRGEVPPRYPR	sp|P02790	2.26E-06	66768.7	74667.3	67810.4	78943.7	96142.6	78987.4	167025.1	164373.6	190996.9
1401	3	381.877	1142.609	7.77	QGHNSVFLIK	sp|P02790	0.004725	4966.6	5088.0	4930.4	7229.6	9339.9	7050.5	7607.1	6476.4	8510.4
1451	3	325.493	973.456	4.14	VDGALCMEK	sp|P02790	0.019636	15542.0	13496.4	15918.4	20341.4	18078.9	20196.4	10967.5	11406.0	11846.4
1495	3	423.564	1267.670	8.13	FDPVRGEVPPR	sp|P02790	3.10E-05	26106.8	28308.1	25714.2	38538.7	48454.7	38281.7	20474.1	20264.4	21712.2
1504	4	711.090	2840.330	15.91	SGAQATWTELPWPHEKV	sp|P02790	1.46E-07	24012.1	24320.9	25588.4	39705.0	45840.2	36605.7	70115.7	67617.5	69147.2
1506	4	625.315	2497.232	14.38	EVGTPHGIILDSVDAAFIC	sp|P02790	0.024329	20880.2	14218.8	18102.9	27536.5	22837.9	23448.4	15697.0	18515.5	16965.3
1666	3	423.564	1267.670	1.45	FDPVRGEVPPR	sp|P02790	0.001028	31999.9	34962.5	33043.6	40913.4	50620.4	42057.2	30633.1	29754.2	34277.6
1670	3	382.551	1144.630	5.67	VWVYPPEKK	sp|P02790	0.006238	18289.7	20563.0	17188.5	24820.4	28555.2	23225.4	24988.2	24440.6	26421.0
1840	2	563.307	1124.600	6.06	QGHNSVFLIK	sp|P02790	0.029967	8197.2	11582.6	8980.8	12446.6	17713.4	12619.3	13529.6	13512.7	17213.1
2156	3	512.266	1533.776	9.66	WKNFPSPVDAAFR	sp|P02790	5.05E-08	6077.4	6725.1	5652.5	7382.6	7999.3	6844.9	33061.1	31848.1	27922.6
2159	3	266.487	796.439	2.82	LHIMAGR	sp|P02790	4.21E-06	1627.5	1461.3	1723.5	3525.6	3440.0	3242.0	2749.2	2642.3	2503.8
2245	2	886.505	1770.995	16.90	LYLVQGTQVYVFLTK	sp|P02790	1.13E-08	35268.9	28550.0	31996.7	59802.1	53965.4	60383.7	10145.0	8780.7	8884.2
2388	5	1105.524	5522.586	10.80	LLQDEFPGIPSPLDAAVE(	sp|P02790	5.87E-05	35648.9	36032.5	46369.0	60056.6	68137.3	62624.0	72281.6	79591.5	93191.5
2830	2	487.735	973.456	6.05	VDGALCMEK	sp|P02790	0.003948	12655.2	11087.1	13137.8	17109.9	15284.3	17399.8	8480.7	8708.5	8528.5
3499	4	783.365	3129.431	12.46	GECQAEGVLFFQGDREW	sp|P02790	2.81E-07	20649.7	16530.2	18214.6	27273.8	26942.5	27627.7	5644.5	5487.2	4249.0
3525	4	361.450	1441.771	6.91	QGHNSVFLIKGDK	sp|P02790	1.20E-07	1524.8	1745.1	1552.1	2203.4	2255.4	2075.1	5981.1	6485.3	6263.7
4172	6	1016.677	6094.020	12.54	DGWHSWPIAHQWPQGP	sp|P02790	3.09E-06	31409.2	29934.7	38986.2	23133.5	23459.7	23708.6	68695.8	71871.6	75681.6
4188	3	1060.152	3177.435	21.52	GECQAEGVLFFQGDREW	sp|P02790	1.26E-07	15783.1	15269.0	15406.9	40068.6	38059.8	44460.5	7910.1	8909.6	6848.9
4946	4	389.706	1554.793	1.38	SHKWDRELISER	sp|P02790	4.07E-09	688.9	671.8	770.4	854.0	949.9	877.5	8815.4	7616.2	7132.3
5279	3	504.897	1511.669	2.28	DVRDYFMPCPGR	sp|P02790	2.78E-06	821.6	984.5	937.7	2027.0	1837.2	1515.5	6643.0	6797.6	4541.6
5387	5	559.473	2792.328	1.40	SGAQATWTELPWPHEKV	sp|P02790	1.67E-06	4467.2	5352.0	4915.8	5734.5	6207.3	5300.7	13741.1	12624.9	14622.4
5574	5	995.297	4971.447	11.07	DGWHSWPIAHQWPQGP	sp|P02790	1.13E-05	20498.8	19153.7	23952.8	17453.2	14652.3	17718.7	37321.7	41189.9	39090.0
6144	4	866.652	3462.579	5.56	GECQAEGVLFFQGDREW	sp|P02790	3.53E-05	6717.9	4017.2	5956.2	6391.6	5497.5	6307.6	12188.3	14140.9	12362.6
9792	4	795.367	3177.438	12.78	GECQAEGVLFFQGDREW	sp|P02790	3.26E-08	3857.5	3508.2	3743.9	9382.7	8878.7	10345.7	1803.6	1838.5	1505.5
9805	3	481.597	1441.769	7.17	QGHNSVFLIKGDK	sp|P02790	1.69E-05	543.2	709.3	563.7	728.6	1147.7	807.4	2418.9	2788.4	2422.4
16838	5	1219.812	6094.023	13.54	DGWHSWPIAHQWPQGP	sp|P02790	4.31E-06	6963.0	6259.7	8084.1	6742.5	6770.7	6134.0	17897.6	15030.5	15987.0
6116	3	665.953	1994.836	10.36	WTPYQGCEALCCPEPK	sp|P04003	0.000102	2897.5	2765.5	3048.0	3960.0	2910.6	3611.3	7057.8	7192.3	9127.7
755	2	336.193	670.371	2.17	TYLFK	sp|P04004	0.000128	11665.3	12824.8	11599.6	9986.6	11205.1	10394.8	11317.9	11115.7	11805.3
858	3	399.858	1196.552	8.52	CTEGFNVDKK	sp|P04004	0.0303	15682.6	13337.6	16117.5	13951.6	13256.4	15283.4	16957.9	17799.5	16204.4
1093	4	871.344	3481.346	16.04	CQCDELCSYYQSCCTDY	sp|P04004	0.00555	102588.0	89437.7	90698.6	74960.2	77677.7	76901.8	82716.5	81310.1	76153.3
1121	2	590.769	1179.524	12.87	CTEGFNVDKK	sp|P04004	0.011056	10869.7	13472.3	12605.8	14699.8	19130.3	15502.0	17382.6	18688.5	17570.4

1907	2	438.241	874.467	3.87	QPQFISR	sp|P04004	0.018255	13522.7	14152.9	12774.1	11937.6	12652.7	12335.8	11419.1	11533.1	12197.0
2401	2	711.831	1421.648	15.06	FEDGVLDPDYPR	sp|P04004	3.61E-08	33420.1	36614.7	34601.4	40092.1	39845.1	38366.9	11977.6	12291.6	10567.3
2996	3	556.265	1665.772	8.17	DWHGVPGQVDAAMAGR	sp|P04004	0.004255	5474.2	6456.4	6072.7	6922.8	9121.4	7908.6	8652.8	8324.5	11407.9
3068	2	506.825	1011.635	14.02	TGISPLALIK	sp|P04114	0.010474	4407.5	4557.4	4819.5	3597.5	5363.7	3782.9	4925.2	4797.9	5044.0
3307	3	822.798	2465.372	19.38	IADFELPTIIVPEQTIEIPSII	sp|P04114	0.000827	12044.6	11948.4	10802.6	13782.2	15970.2	12943.4	9826.1	10279.7	10349.1
3623	3	514.296	1539.865	5.98	LLSGGNTLHLVSTTK	sp|P04114	0.030521	4256.7	6815.3	4243.8	4314.9	6659.5	4609.0	3497.2	3529.3	4176.0
4639	2	371.733	741.451	1.36	AAIQALR	sp|P04114	0.005115	1203.4	1422.7	1261.9	1273.5	1437.8	1263.0	1600.9	1682.4	1581.0
4751	2	431.246	860.477	5.69	ITLPDFR	sp|P04114	1.59E-06	1590.4	2098.6	1894.9	2407.4	2326.4	2691.2	607.6	693.8	501.3
5119	2	569.781	1137.547	3.81	QGFFPDSVNK	sp|P04114	0.000737	5441.2	8403.6	7136.0	8224.6	10537.3	8664.7	2829.8	2743.8	3129.9
8667	3	719.719	2156.135	14.46	HSITNPLAVLCEFISQSIK	sp|P04114	0.004527	4508.0	3873.7	5332.1	4411.0	3377.6	3888.6	5890.8	6194.0	5658.8
9593	3	1147.589	3439.746	17.90	YSQPEDSLIPFFEITVPES	sp|P04114	0.000302	8111.6	7836.5	6685.7	12828.4	11587.6	11350.7	8740.6	7366.5	8238.4
14117	4	860.944	3439.748	15.18	YSQPEDSLIPFFEITVPES	sp|P04114	0.002157	3127.8	2957.7	2941.9	4449.9	4286.9	4405.2	3479.4	3128.2	3612.7
16190	4	915.950	3659.772	14.44	NKADYVETVLDSTCSSTV	sp|P04114	1.01E-06	3086.0	2997.2	3185.7	4651.7	3971.1	4041.6	862.1	892.7	572.0
21313	5	1026.753	5128.731	8.99	LPEIAIPEFIIPTLNLNDFQ	sp|P04114	0.000377	2658.5	3248.0	2838.3	3235.9	2565.6	2468.7	436.9	299.3	555.9
33042	4	743.177	2968.681	15.99	VHANPLLIDVVTYLVALIPI	sp|P04114	1.18E-08	520.1	614.9	554.7	693.6	603.4	669.3	120.5	132.5	114.2
951	2	562.810	1123.605	7.64	DGYLFQLLR	sp|P04196	0.042669	17926.8	22435.1	18742.1	18159.3	15298.1	18671.0	25070.6	17760.7	14986.8
1337	3	507.561	1519.661	5.89	YKEENDDFASFR	sp|P04196	6.87E-08	15646.5	16265.6	15370.7	19824.9	19793.8	20178.9	11881.5	11449.3	12826.3
1488	3	608.970	1823.887	15.67	ADLFYDVEALDLESPK	sp|P04196	0.000572	20927.9	19056.9	20657.9	24073.1	21052.8	24501.6	22209.6	24345.7	22356.4
1508	3	314.863	941.568	3.27	ALDLINKR	sp|P04196	0.002465	5491.7	7706.3	5416.1	5180.8	7709.8	5312.2	4788.9	4380.9	4532.5
1548	2	411.728	821.441	6.61	QIGSVYR	sp|P04196	0.001268	5704.1	6256.3	5959.5	6649.5	6154.8	6200.7	7489.6	6707.5	6077.6
1573	3	429.568	1285.683	5.60	RPSEIVIGQCK	sp|P04196	0.001974	9325.9	7792.3	9509.8	10956.0	10206.4	10897.0	12117.5	10663.0	10071.2
1661	2	745.850	1489.686	16.06	GGEGTGYFVDFSVR	sp|P04196	0.000284	22585.1	18506.9	21269.7	22855.5	20390.4	21432.1	24497.6	23659.5	22454.0
1770	3	378.516	1132.526	1.40	HPNVFGFCR	sp|P04196	0.00021	5897.0	6084.8	5606.5	6415.7	7074.7	5888.5	8851.5	7588.0	9908.8
2423	2	912.950	1823.886	17.90	ADLFYDVEALDLESPK	sp|P04196	0.003746	19249.9	18383.7	22659.8	26195.7	22540.1	28490.9	22320.5	25099.8	21717.0
2613	2	841.900	1681.786	17.06	DSPVLIDFFEDTER	sp|P04196	0.00065	14612.9	15650.1	21625.2	22762.7	18876.9	25326.0	12014.5	11120.2	11241.7
3979	3	561.603	1681.787	9.95	DSPVLIDFFEDTER	sp|P04196	0.000864	5098.3	5354.6	6903.3	8227.4	5930.4	8822.9	4332.4	3698.9	3762.8
4115	4	473.472	1889.858	6.78	YKEENDDFASFRVDR	sp|P04196	2.09E-06	2060.0	2630.0	2924.5	1704.2	2247.3	1824.8	10417.7	9334.1	10415.1
6544	3	667.991	2000.953	10.50	DSPVLIDFFEDTERYR	sp|P04196	0.00079	3057.5	2825.5	3287.0	3049.9	2280.9	3103.0	5068.8	4703.8	4659.4
1079	2	632.831	1263.648	16.06	SGLSTGWTQLSK	sp|P04217	0.007366	27363.4	21082.0	23785.2	17796.2	21396.9	16724.6	22073.0	21594.5	25377.6
1223	4	537.768	2147.042	12.80	IFFHLNAVALGDGGHYTC	sp|P04217	0.04644	23843.4	28807.2	21503.8	14750.9	21238.6	14461.5	17100.2	17035.3	14915.0
1674	2	403.251	804.488	3.95	GVTFLLR	sp|P04217	0.011604	4686.8	5861.9	4825.3	4145.2	4081.7	3867.5	5436.7	5151.4	7073.1
1839	3	626.006	1874.996	17.11	VTLTCVAPLSGVDFQLR	sp|P04217	5.40E-05	18641.4	17355.2	16952.2	13041.6	11407.0	11777.1	12430.7	11344.8	11287.9
1903	2	363.730	725.445	2.22	ELLVPR	sp|P04217	0.020106	4402.4	4962.3	4412.8	2753.2	4468.6	3095.5	4422.3	4228.1	4072.8
2105	3	824.405	2470.194	12.43	SWVPHTFESELSDPVELL	sp|P04217	0.00559	26632.6	18922.6	27868.7	20915.5	23803.4	20572.2	17430.2	16149.8	18622.2

5175	2	1236.104	2470.193	8.90	SWVPHTFESELSDPVELL	sp|P04217	0.020631	14868.7	11648.2	16520.2	12225.9	15741.7	11767.9	9554.3	8761.8	12161.7
8821	2	814.905	1627.795	8.61	CEGPIPDVTFELLR	sp|P04217	0.002514	3254.4	2686.6	3585.2	3058.9	3047.9	3301.6	1708.6	2172.8	1542.3
13674	2	1076.088	2150.161	16.95	SLPAPWLSMAPVSWITP(	sp|P04217	0.003245	2511.6	2166.5	2975.8	3283.1	2601.1	3101.1	2249.6	2836.7	2639.0
297	2	455.736	909.457	6.33	TLYSSSPR	sp|P05155	5.73E-05	43870.1	38363.1	40837.1	47682.1	51528.6	43038.4	68065.0	64891.0	70019.0
377	2	558.799	1115.584	13.15	LLDSLPSDTR	sp|P05155	0.02121	37880.0	46982.4	47016.8	51334.3	72082.2	49107.2	40024.7	33493.4	43120.5
474	2	593.354	1184.694	10.79	FQPTLLTLPR	sp|P05155	0.000506	57333.8	56354.2	57634.9	75442.1	66785.0	67732.4	47981.3	52927.2	44140.9
571	3	609.665	1825.972	16.90	GVTSVSQIFHSPDLAIR	sp|P05155	7.47E-05	51097.9	59454.7	52635.3	69494.1	96969.5	67915.1	32696.3	26338.1	24007.5
640	3	406.870	1217.589	7.54	DFTCVHQALK	sp|P05155	8.96E-08	23218.6	23119.2	21970.3	25324.7	26849.7	26344.1	14283.8	13918.2	14832.8
823	2	659.412	1316.810	15.06	LVLLNAIYLSAK	sp|P05155	0.001438	33943.1	29421.0	33505.8	44408.3	32914.6	45196.5	28726.2	26521.2	24208.5
902	2	325.169	648.324	2.00	VYDPR	sp|P05155	0.03362	9141.5	7834.1	9412.7	10077.3	8200.2	10312.4	10848.5	11253.3	12356.1
904	4	616.820	2463.251	12.54	TNLESILSYPKDFTCVHQ/	sp|P05155	8.14E-06	23911.3	30054.9	28308.9	21366.2	17611.8	21758.6	64912.8	82133.0	65492.2
3009	3	395.905	1184.694	1.82	FQPTLLTLPR	sp|P05155	0.002516	4054.2	3568.2	4069.4	5103.8	4232.5	4574.3	3391.6	3612.6	3596.1
4515	3	872.473	2614.398	18.90	TLLVFEVQQPFLFVLWDC	sp|P05155	2.76E-09	12328.1	10286.0	11484.4	18186.0	17334.2	16000.5	2484.2	2407.7	2239.0
6933	3	1022.879	3065.615	12.74	KVETNMAFSPFSIASLLT(	sp|P05155	1.55E-05	6851.0	6441.8	7141.9	13827.4	12705.0	12541.8	11158.9	12519.6	9224.9
12137	4	755.410	3017.613	8.14	KVETNMAFSPFSIASLLT(	sp|P05155	0.004815	3639.5	2819.7	3433.2	4369.3	4151.7	3984.7	3229.2	3027.5	2354.2
13249	4	654.607	2614.399	8.20	TLLVFEVQQPFLFVLWDC	sp|P05155	6.54E-07	1369.6	1447.4	1325.0	2443.7	2140.3	2072.1	204.5	213.4	351.6
15562	4	767.411	3065.616	16.90	KVETNMAFSPFSIASLLT(	sp|P05155	2.10E-06	1451.7	1506.4	1503.1	2919.4	2555.0	2682.4	2445.7	2751.2	2256.4
16584	3	805.801	2414.382	15.91	LLDSLPSDTRLVLLNAIYL	sp|P05155	5.96E-05	703.4	644.0	993.3	1152.8	956.7	896.2	3176.2	3325.8	2863.0
4657	2	560.825	1119.635	7.49	QFPILLDFK	sp|P05546	6.12E-06	3501.7	3199.0	3712.9	3742.1	3315.8	3404.3	2028.7	2136.3	1969.3
5851	3	761.060	2280.158	12.90	AILQSGSFNAPWAVTSLY	sp|P06276	0.000979	6869.6	6902.8	7833.2	7231.6	7460.1	7217.6	4975.5	4701.5	5681.3
4020	3	677.321	2028.942	14.64	DSGVPDRFSGSGSGTDF	sp|P06310	0.000624	6099.5	6759.4	6317.2	4554.6	5377.8	5690.6	8928.4	9101.1	11986.4
10267	2	373.178	744.341	4.47	DSGVPDR	sp|P06310	1.53E-05	2646.6	2339.0	2931.8	2842.0	2196.9	2608.5	1224.6	1257.9	1128.1
1942	2	561.820	1121.625	11.17	LLIYWASTR	sp|P06312	0.009812	10539.9	12540.5	11302.1	8918.4	11849.2	9631.1	9893.8	10385.5	9751.5
2711	2	380.186	758.357	1.45	ESGVPDR	sp|P06312	0.000174	4565.0	3851.5	4428.7	4747.4	4001.2	4938.7	1480.3	1749.0	1422.6
20795	2	364.701	727.388	2.07	VNVGDPK	sp|P06681	0.000683	696.7	584.1	705.9	657.0	516.6	700.9	1064.6	1040.6	1061.7
27809	2	674.868	1347.722	5.54	QHLGDVLNFLPL	sp|P06681	0.012116	252.5	192.7	317.6	291.8	222.3	282.5	409.5	440.3	391.7
2916	2	882.963	1763.912	16.90	WSAGLTSSQVDLYIPK	sp|P08185	0.038504	18048.5	12055.7	17571.4	16550.5	11402.2	16503.8	10223.2	10453.5	8470.3
10265	4	701.872	2803.460	18.78	KNIFISPVSISMALAMLSL(	sp|P08185	0.001137	3735.5	3668.6	4197.3	3882.8	3103.3	3612.2	2815.0	2688.4	2694.1
11761	3	892.795	2675.363	15.17	NIFISPVSISMALAMLSLG	sp|P08185	2.08E-05	3280.9	2881.7	3007.7	4568.5	4381.0	4349.8	2555.9	2210.6	2158.7
21376	3	910.803	2729.387	16.17	FNQPFIIMIFDHFTWSSLF	sp|P08185	1.89E-05	1065.1	1290.1	1395.2	877.0	686.9	858.8	2054.9	2198.2	2052.8
3742	3	509.927	1526.758	9.81	SIDVACHPGYALPK	sp|P08603	6.08E-05	3112.1	3503.1	3093.2	3311.7	3817.4	3276.6	4847.3	4869.4	5229.3
2305	2	656.847	1311.680	10.99	LGNQEPGGQTALK	sp|P08697	1.94E-06	16033.2	15229.5	15084.0	12081.8	12710.2	11146.0	7893.4	8117.8	7466.1
3451	3	710.380	2128.119	13.90	WFLLEQPEIQVAHFPFK	sp|P08697	9.73E-06	19759.3	15235.9	18359.1	16242.3	11132.7	14741.0	5068.4	4134.9	4458.5
5901	5	884.657	4418.250	6.35	GFPIKEDFLEQSEQLFGA	sp|P08697	0.000371	12180.4	11861.5	11657.9	9655.9	13137.3	8713.9	5844.3	4769.6	4973.4

17187	3	908.479	2722.415	12.22	HQMDLVATLSQLGLQELF	sp|P08697	0.001135	1286.7	1496.4	1679.5	1994.5	2099.2	2025.4	734.3	1178.0	826.5
1358	2	684.365	1366.716	15.06	DSSTWLTAFVLK	sp|P0C0L4	0.000142	12192.7	16140.6	12276.1	35920.3	26061.5	33538.5	21376.7	20951.5	18393.5
2633	3	1044.830	3131.467	19.99	LQETSNWLLSQQQADGS	sp|P0C0L4	4.69E-05	21880.3	20300.8	24249.1	41963.7	33154.9	45211.1	17353.3	18027.3	17526.8
4375	3	543.320	1626.938	12.10	PVAFSVVPTAAAAVSLK	sp|P0C0L4	1.31E-06	3575.8	2961.6	3088.5	7405.8	5044.1	8621.7	698.4	890.0	796.6
5569	3	456.579	1366.716	7.43	DSSTWLTAFVLK	sp|P0C0L4	6.14E-05	1289.0	1496.9	1429.6	3614.2	2564.0	2941.3	1976.4	2070.7	1987.4
6055	2	814.475	1626.936	11.89	PVAFSVVPTAAAAVSLK	sp|P0C0L4	1.17E-05	4396.3	3551.0	3561.9	8945.6	5823.5	10232.7	832.2	1120.5	590.0
1959	4	775.140	3096.531	16.99	LQETSNWLLSQQQADGS	sp|P0C0L5	0.002937	19850.8	14884.8	18956.3	32705.5	26180.6	28368.5	16730.9	19059.4	19061.3
2535	3	1033.184	3096.530	21.78	LQETSNWLLSQQQADGS	sp|P0C0L5	0.002842	22348.2	15961.2	20856.9	36583.5	30389.5	33156.9	16178.7	20707.1	19962.6
3021	2	655.363	1308.711	14.06	GSSTWLTAFVLK	sp|P0C0L5	0.000393	6582.6	5779.8	6652.8	4719.9	4835.7	5809.5	8734.8	7476.8	7410.8
4416	5	924.874	4619.332	11.17	VLSLAQEQVGGSPEKLQI	sp|P0C0L5	1.26E-06	10306.4	9173.8	8700.6	11795.2	10624.4	12190.5	32476.3	31440.6	27397.5
889	3	772.065	2313.174	15.17	VTTVASHTSDSDVPSGVT	sp|P10909	0.014798	48691.8	45741.0	51593.3	46279.5	65279.2	49828.3	68923.2	62627.2	70661.8
1499	2	644.824	1287.633	13.83	ELDESLQVAER	sp|P10909	0.001393	13714.2	18813.6	16838.4	15735.9	13557.1	14527.5	21103.0	20398.2	23048.5
1937	2	697.353	1392.691	15.06	ASSIIDELFQDR	sp|P10909	0.000601	17012.7	15695.9	18469.4	16144.5	13828.0	16425.1	10630.6	11544.1	10580.2
2168	2	356.177	710.340	3.63	SYQWK	sp|P10909	7.46E-05	4257.1	4208.9	4051.7	3990.9	4113.3	4010.2	4671.4	5065.8	4862.9
3272	2	536.786	1071.557	7.28	EIQNAVNGVK	sp|P10909	0.00236	4465.6	3912.9	4289.0	4316.9	4303.9	4538.3	3319.8	3635.0	3503.9
4999	4	500.748	1998.964	4.44	EPQDTYHYLPFSLPHR	sp|P10909	0.039649	5059.7	5849.8	5009.2	4330.2	4735.5	4374.8	4824.7	4468.4	3343.0
5651	3	408.176	1221.506	3.40	EGDDDRTVCR	sp|P10909	0.023444	3764.3	3195.0	3913.6	3993.8	3098.3	3348.9	4690.3	4623.6	5144.0
8809	3	649.000	1943.979	9.37	ASSIIDELFQDRFFTR	sp|P10909	1.20E-06	1795.5	1603.6	1596.2	1194.2	958.2	1039.3	3059.2	4125.7	3480.5
186	4	309.432	1233.700	4.66	EHVAHLLFLR	sp|P19652	0.03369	45393.2	57683.5	50966.9	49024.7	66687.4	46709.1	32044.6	36115.3	45747.2
368	3	408.869	1223.584	7.88	SDVMYTDWKK	sp|P19652	5.35E-06	46927.0	47841.3	44937.9	46949.0	42637.3	45140.7	74643.2	71748.5	67236.5
416	2	548.752	1095.489	6.07	SDVMYTDWK	sp|P19652	0.002664	62464.1	61066.6	64538.5	58687.6	67299.0	56275.5	54549.0	55572.4	55517.4
534	3	704.997	2111.970	16.11	EQLGEFYEALDCLCIPR	sp|P19652	4.69E-05	119838.2	84541.4	106607.6	126271.6	88544.7	107859.6	36772.1	40900.7	37663.7
550	5	360.197	1795.951	4.10	YEGGREHVAHLLFLR	sp|P19652	6.06E-05	16464.8	21929.7	18226.8	15541.6	24585.5	18037.8	53486.0	49198.0	62575.1
592	4	449.995	1795.951	6.33	YEGGREHVAHLLFLR	sp|P19652	0.005722	20899.8	35258.5	22467.6	17325.5	37763.3	21787.6	62878.5	56153.1	88465.1
973	3	366.170	1095.489	2.12	SDVMYTDWK	sp|P19652	0.004652	14775.5	13952.1	15837.3	14063.3	15828.2	13303.6	13392.8	13427.9	12968.1
1081	3	424.870	1271.588	9.32	SDVMYTDWKK	sp|P19652	1.07E-05	9056.2	9124.1	9157.1	11855.2	13904.2	11438.1	19411.9	18454.5	20793.7
1648	5	622.503	3107.478	7.16	TLMFGSYLDDEKNWGLS	sp|P19652	2.47E-09	32285.9	34052.1	32080.5	37534.1	34738.7	35236.9	9583.8	10344.3	10617.7
1865	2	1056.991	2111.967	16.95	EQLGEFYEALDCLCIPR	sp|P19652	7.44E-05	59970.5	38662.2	48714.5	67165.8	43115.7	55758.1	15742.6	18240.8	16211.5
2348	5	1050.895	5249.439	5.99	TLMFGSYLDDEKNWGLS	sp|P19652	1.41E-05	46527.7	42303.6	46369.8	65708.8	61604.5	60517.9	92650.0	125090.8	103071.5
2368	4	789.879	3155.486	6.78	TLMFGSYLDDEKNWGLS	sp|P19652	6.37E-06	24838.6	20995.6	25258.2	40026.1	33569.1	38953.6	12288.7	12748.2	9598.8
651	3	494.592	1480.755	10.86	IYGNQDTSSQLKK	sp|P19823	0.000507	22614.7	22097.3	19412.8	24780.3	22369.2	26236.3	38372.8	37579.7	30885.6
655	2	791.932	1581.849	17.90	IQPSGGTNINEALLR	sp|P19823	0.014859	55030.4	66928.4	58370.1	59862.7	74717.9	61624.1	72390.2	62013.8	71906.1
662	2	669.365	1336.716	11.05	FYNQVSTPLLR	sp|P19823	0.000737	30448.7	28962.7	34464.9	35743.3	28205.4	37379.6	52027.7	53201.6	49086.1
938	4	921.695	3682.749	16.09	NVQFNYPHTSVTDVTQNI	sp|P19823	0.000188	90952.6	80375.6	93459.8	103755.2	91373.1	103612.1	56780.6	60940.9	57770.4

1198	2	396.722	791.430	2.24	ALYAQAR	sp|P19823	0.025929	7100.0	4964.5	6589.2	6934.6	7302.3	7422.6	8714.1	8831.8	7860.7
1200	3	473.915	1418.722	7.74	VQFELHYQEVK	sp|P19823	0.00118	11927.7	16760.4	15557.0	18036.2	20473.7	16901.9	11872.4	10903.9	13513.1
1255	2	423.746	845.478	5.87	IYLQPGR	sp|P19823	0.001836	9474.4	10787.0	9478.5	11167.9	13515.7	10344.2	8787.9	8877.0	9095.3
1260	4	367.957	1467.797	3.50	AHVSFKPTVAQQR	sp|P19823	0.000128	10254.7	9898.7	9832.1	13299.1	11579.5	11545.6	15282.7	14598.6	14110.6
1373	3	611.354	1831.039	11.95	KLWAYLTINQLLAER	sp|P19823	0.026986	23668.3	20858.4	21184.7	30832.3	27496.4	25559.5	25807.5	23948.6	23312.6
1440	2	415.243	828.471	8.97	SLAPTAAAK	sp|P19823	0.0036	7808.8	7166.6	8447.7	9912.3	9361.0	9256.4	8869.7	8457.5	8279.8
1534	3	597.325	1788.954	7.38	HLEVDVWVIEPQGLR	sp|P19823	9.70E-05	18583.0	16622.4	19024.1	24663.1	21922.7	23633.5	19738.4	22395.7	19850.4
1715	2	514.294	1026.573	7.19	TEVNVLPGAK	sp|P19823	0.000328	11736.6	12936.5	11872.4	12977.0	18183.4	15390.6	8399.3	8406.8	8957.3
2417	3	946.139	2835.396	20.17	AGELEVFNGYFVHFFAPD	sp|P19823	0.00357	34006.2	36767.3	31767.7	49223.7	37122.7	44858.4	27180.8	29052.2	23854.9
2482	3	490.273	1467.797	9.91	AHVSFKPTVAQQR	sp|P19823	0.000205	6906.2	7181.4	6988.8	8666.5	8792.3	8184.2	10906.1	9776.1	9158.7
2750	3	528.291	1581.851	10.66	IQPSGGTNINEALLR	sp|P19823	0.002706	6372.1	6960.3	7175.6	6906.9	7599.0	7073.7	8914.2	8124.7	8479.6
4605	2	308.693	615.372	1.46	TAGLVR	sp|P19823	0.034328	4369.0	3790.3	4534.6	5143.3	4041.2	4964.3	6166.5	6357.0	6352.7
7509	3	568.655	1702.944	9.72	LWAYLTINQLLAER	sp|P19823	0.005256	2949.5	1835.8	2418.5	3328.8	2674.8	3397.5	2251.3	2261.5	2050.1
8629	4	709.856	2835.396	12.43	AGELEVFNGYFVHFFAPD	sp|P19823	0.004303	4305.9	4478.0	4022.1	6454.3	4219.2	5499.6	2931.2	3416.8	3223.5
21826	4	1138.832	4551.301	11.15	FDPAKLDQIESVITATSAN	sp|P19823	0.000303	2042.2	1880.6	1904.2	3627.2	2609.4	3227.7	1506.7	1426.9	1407.6
23461	4	941.766	3763.036	16.58	AIFILNEANNLGLLDPNSV	sp|P19823	0.000128	4524.5	4429.6	4749.8	5916.8	5062.0	5681.2	3341.1	2709.8	2180.4
25831	5	911.267	4551.300	14.31	FDPAKLDQIESVITATSAN	sp|P19823	0.000168	993.5	960.5	856.3	1562.1	1136.7	1366.5	536.2	611.0	684.6
28928	3	1255.352	3763.033	15.30	AIFILNEANNLGLLDPNSV	sp|P19823	0.000105	4930.9	4940.3	4531.5	6375.0	5634.8	6481.4	3285.3	3000.0	2211.5
824	3	669.341	2005.001	6.88	FAHYVVTSQVVNTANEA	sp|P19827	0.01996	49578.9	51129.9	43566.4	51137.6	72290.7	52335.2	45834.9	39653.8	55958.8
1004	2	580.815	1159.615	12.71	EVAFDLEIPK	sp|P19827	0.030678	21815.0	19602.6	21965.5	21353.2	20044.6	23252.0	19160.9	15528.7	12442.3
1087	2	579.318	1156.622	16.06	AAISGENAGLVR	sp|P19827	0.038338	18093.1	20709.5	18777.4	19881.3	21568.4	18230.7	18671.1	18077.8	20115.4
1132	2	735.865	1469.716	18.11	QYYEGSEIVVAGR	sp|P19827	0.001122	29284.3	35609.9	31239.3	36768.6	38060.8	32086.3	46944.4	40488.4	53827.4
1250	2	545.302	1088.589	13.98	LDAQASFLPK	sp|P19827	0.001588	26043.7	29848.1	25767.2	29336.3	32317.2	28183.5	20628.9	18146.3	22996.7
1397	2	420.217	838.419	5.47	VTYDVSR	sp|P19827	3.30E-05	8513.9	10005.6	8647.1	10498.5	11232.7	11101.2	4893.7	5579.7	6193.9
1442	6	842.430	5048.539	13.06	IYEDHDATQQLQGFYSQ	sp|P19827	0.003899	74132.8	61094.7	73361.2	87660.3	71244.8	77170.2	99795.4	100866.6	94052.6
2178	2	781.454	1560.894	17.06	LWAYLTIQELLAK	sp|P19827	0.018584	19394.1	14946.2	17438.6	22141.7	18382.6	20147.3	16345.8	16388.0	11436.8
2188	2	839.427	1676.839	16.90	GFSLDEATNLNGGLLR	sp|P19827	0.017484	20955.2	21392.1	18385.1	28031.6	22770.6	24899.0	20362.7	18351.4	19725.1
2286	3	429.246	1284.717	5.77	KAAISGENAGLVR	sp|P19827	0.00091	6320.0	6348.7	5783.5	7420.9	7605.7	7182.9	7256.0	7481.2	7362.5
2573	3	542.636	1624.886	10.86	VTFQLTYEEVLKR	sp|P19827	3.48E-07	10287.3	10741.2	9384.6	13450.8	14043.9	11207.7	4053.2	3914.7	3495.3
2627	3	521.306	1560.895	11.32	LWAYLTIQELLAK	sp|P19827	0.011047	7909.6	5940.3	7256.6	9377.4	7648.7	8171.1	7554.0	7952.0	5930.1
3286	3	334.539	1000.594	4.15	ELAAQTIKK	sp|P19827	0.021365	1830.1	1569.4	2038.3	2070.8	1862.4	2287.2	2884.6	3189.1	3885.0
3500	4	751.140	3000.530	16.65	TAFISDFAVTADGNAFIGD	sp|P19827	7.31E-06	11944.4	9792.1	10380.8	10946.9	9713.1	10002.4	23095.0	20992.5	19141.1
4839	5	663.934	3314.633	10.71	KGHVLFRPTVSQQQSCP	sp|P19827	0.005237	8688.6	12977.2	9024.1	11401.7	15337.2	14127.7	8164.6	7237.2	7374.0
5320	3	328.535	982.583	5.41	ELAAQTIKK	sp|P19827	0.035645	977.9	865.4	1063.4	1072.3	990.2	1222.8	1398.2	1584.1	1917.5

8744	4	1002.009	4004.008	13.77	GIEILNQVQESLPELSNH	sp|P19827	7.50E-05	11087.2	10130.5	11545.2	25819.6	21092.1	20625.5	11380.9	11165.3	8618.0
10014	3	719.696	2156.066	11.45	ILGDMQPGDYFDLVLFGT	sp|P19827	0.00044	1383.4	1216.4	1408.0	2420.1	2672.9	3349.7	1793.7	1931.4	1516.7
10027	3	725.033	2172.079	19.11	TAFISDFAVTADGNAFIG	sp|P19827	4.78E-06	5470.1	4897.8	5249.3	7711.1	5767.7	6482.7	2393.7	2378.7	2364.8
18692	2	1079.039	2156.063	16.90	ILGDMQPGDYFDLVLFGT	sp|P19827	0.000284	903.5	955.7	1055.4	1780.3	1978.0	2430.6	1025.8	1194.2	1229.8
2183	3	848.703	2543.087	17.38	SQCTYSNPEGTVVLACD	sp|P22792	0.000537	32188.3	34201.8	34240.6	26508.0	24408.7	25390.3	38310.0	30448.6	38237.6
2649	2	801.437	1600.860	16.90	LSNNALSGLPQGVFGK	sp|P22792	0.014114	15828.8	14248.0	17055.8	15457.4	14250.1	14760.1	11987.0	13983.7	11108.7
2719	3	812.428	2434.262	15.90	VVFLNTQLCQFRPDAFG	sp|P22792	0.013707	25545.7	18675.1	20989.1	23626.0	15157.4	18704.6	12980.4	13796.2	13144.0
4557	4	762.182	3044.697	16.99	TLNLAQNLLAQLPEELFH	sp|P22792	0.004253	9363.4	9014.6	9099.7	11343.1	9519.9	8564.7	10744.2	9886.5	10633.8
4675	2	617.285	1232.556	11.01	CLAYDFYPGK	sp|P25311	0.00593	3598.9	3951.2	3280.3	2168.1	2474.8	2299.3	3653.5	2682.1	3254.4
4746	3	801.743	2402.206	15.90	HVEDVPAFQALGSLNDL	sp|P25311	0.007464	10720.3	11551.3	11798.4	8926.0	6794.4	9219.0	10823.5	11049.5	12517.4
10383	3	800.759	2399.255	15.38	ILLMDLNEEDPTVLELGIT	sp|P27169	1.01E-07	3055.2	3047.8	2839.8	3880.1	3632.8	3787.0	1680.8	1578.7	1571.6
6521	2	657.845	1313.676	4.12	DFALQNPSAVPR	sp|P35858	0.004081	3404.3	3341.4	3360.8	2314.0	2514.9	2485.5	2870.9	2583.2	3425.8
7338	2	727.942	1453.869	9.17	NLIAAVAPGAFLGLK	sp|P35858	4.44E-07	3054.8	3858.0	3269.6	3095.4	3031.6	2641.9	776.1	792.3	635.5
7382	4	334.447	1333.761	1.39	LSHNAIASLRPR	sp|P35858	0.003474	1138.2	1126.4	1004.7	857.7	997.8	868.4	745.6	794.5	758.3
9511	2	835.979	1669.943	16.90	VAGLLEDTFPGLLGLR	sp|P35858	6.98E-05	3139.4	3002.2	3260.3	2919.5	2210.3	3089.6	895.2	1074.8	844.4
13634	3	557.655	1669.943	12.16	VAGLLEDTFPGLLGLR	sp|P35858	1.45E-05	1316.7	1111.7	1233.5	1036.4	812.8	955.2	319.9	397.6	271.6
29174	4	953.498	3809.965	19.09	DNGLVGIEEQSLWGLAE	sp|P35858	0.002982	995.3	917.9	1150.5	767.0	824.8	749.9	682.1	689.9	809.7
1550	2	527.799	1053.584	4.29	LPNNVLQEK	sp|P43652	0.000363	21416.4	23601.2	21869.9	16390.4	23661.4	17087.8	10159.8	10601.2	12150.0
1682	3	530.948	1589.823	10.83	ESLLNHFLYEVAR	sp|P43652	3.63E-07	18752.1	16481.3	15209.9	12944.2	11384.4	11593.9	6080.9	5986.0	5937.9
1719	3	642.614	1924.821	15.64	SCCEEQNKVNCLQTR	sp|P43652	0.00143	23184.6	23818.1	24711.6	16787.2	15471.8	17385.4	18274.1	20172.2	17202.7
1737	4	673.348	2689.364	7.56	LKHELTDEELQSLFTNFA	sp|P43652	0.002155	25778.2	27719.3	31812.4	22668.1	30450.5	22518.1	16925.3	17010.0	17507.3
1950	2	811.855	1621.695	17.06	AESPEVCFNEESPK	sp|P43652	3.05E-08	27947.9	26728.5	28058.3	22231.2	25558.5	26302.4	8733.9	8699.2	8834.7
1957	3	438.870	1313.587	9.73	TINPAVDHCCK	sp|P43652	0.005097	9561.5	8940.8	10268.6	8003.6	6868.9	7166.4	7098.1	7476.0	5878.1
2100	2	692.794	1383.574	12.15	FTDSENVCQER	sp|P43652	1.35E-07	17766.8	18675.6	18916.8	16331.0	12601.4	13985.0	4152.5	3593.4	3478.4
2373	4	411.959	1643.807	2.10	GQCIINSNKDDRPK	sp|P43652	0.00529	7763.0	8002.8	7896.9	5782.0	4790.8	6520.1	5350.4	6152.2	5744.3
2639	4	622.097	2484.358	12.07	RNPFVFAPTLLTVAVHFE	sp|P43652	0.005107	19971.3	15635.2	17906.1	16181.6	13412.8	15292.0	12353.7	12185.1	11734.1
3097	2	516.809	1031.603	1.33	AIPVTQYLK	sp|P43652	2.83E-05	9561.2	12002.3	9502.2	8392.1	10184.1	8499.5	3095.4	3998.6	4092.7
4074	3	366.859	1097.556	4.06	ICAMEGLPQK	sp|P43652	7.41E-07	3213.4	2570.9	3179.8	2502.6	2637.2	2455.1	957.4	761.6	850.5
4192	3	831.362	2491.063	18.64	FTDSENVCQERDADPDT	sp|P43652	2.90E-06	6940.1	8333.5	8017.1	3661.7	4249.7	3404.2	13103.7	11820.5	15776.5
4221	2	944.474	1886.933	15.90	SDVGFLPPFPTLDPEEK	sp|P43652	0.000143	18805.8	17862.4	15709.0	13887.7	11763.5	13616.5	8969.3	9778.1	8584.1
4689	2	514.218	1026.421	7.92	CQAYESNR	sp|P43652	3.28E-06	9013.8	7830.8	9732.8	6661.7	5851.8	6548.0	3521.5	3509.1	2950.9
4920	2	415.706	829.398	4.72	AFSSYQK	sp|P43652	4.78E-08	2266.0	1961.2	2464.9	2187.6	1778.0	2111.9	415.3	342.3	374.8
6666	3	629.985	1886.935	14.67	SDVGFLPPFPTLDPEEK	sp|P43652	7.53E-05	5359.5	4984.9	4897.2	4438.0	3408.2	3962.7	2560.5	2912.5	2695.7
6953	3	1006.117	3015.328	22.04	ELISLVEDVSSNYDGCCE	sp|P43652	1.67E-09	15357.4	16891.0	18425.9	16077.6	17136.6	14970.7	1757.4	1617.8	1990.5

8075	3	566.919	1697.734	7.39	EGKFTDSENVCQER	sp|P43652	0.007777	2268.6	1957.3	1775.0	1617.6	1570.9	1232.5	1361.4	1493.8	1624.2
12335	4	754.840	3015.331	12.32	ELISLVEDVSSNYDGCCE	sp|P43652	1.56E-05	5205.6	3857.9	5444.8	3942.8	5098.5	4829.7	500.0	445.8	183.9
34	2	657.837	1313.659	15.06	VNVDEVGGEALGR	sp|P68871	0.000178	364307.8	317298.8	324504.9	458338.8	471184.3	437947.9	598138.3	515895.8	676254.5
46	2	466.765	931.515	11.08	SAVTALWGK	sp|P68871	0.039258	151270.3	199594.4	144741.1	173235.9	221299.2	166629.8	196360.9	204967.8	272198.6
59	4	445.005	1775.989	11.95	LLGNVLVCVLAHHFGK	sp|P68871	5.54E-05	208965.6	184525.8	185442.9	181185.6	191111.3	204833.7	307944.7	316603.7	282796.9
101	2	689.855	1377.695	15.06	EFTPPVQAAYQK	sp|P68871	0.000531	270712.9	275449.3	286098.6	326429.4	431918.6	328345.2	431225.9	395870.0	450940.1
267	3	438.894	1313.660	9.42	VNVDEVGGEALGR	sp|P68871	1.23E-05	39091.7	33869.3	35677.0	51742.2	49132.9	48329.2	67017.7	60893.9	73009.5
328	3	460.239	1377.696	10.02	EFTPPVQAAYQK	sp|P68871	3.98E-06	50788.3	47458.9	55488.4	60603.2	67233.6	60578.1	82202.9	80260.9	86359.6
418	3	670.987	2009.939	18.11	FFESFGDLSTPDAVMGNI	sp|P68871	0.007239	71009.5	107248.9	89242.1	85932.7	82286.9	94664.8	121591.6	127462.7	110908.6
439	5	518.055	2585.238	1.47	GTFATLSELHCDKLHVDP	sp|P68871	0.021653	59809.6	63086.8	60567.8	66486.1	84685.6	67166.8	74963.5	70461.6	78369.6
499	3	686.989	2057.945	17.11	FFESFGDLSTPDAVMGNI	sp|P68871	3.01E-05	45577.9	39147.8	43221.2	73262.1	61689.1	77622.0	91517.1	81945.7	73305.0
519	3	593.004	1775.989	11.06	LLGNVLVCVLAHHFGK	sp|P68871	0.001011	47290.3	44466.3	40398.3	43169.0	41988.9	52972.4	68188.9	71406.7	56330.3
797	2	1029.979	2057.943	17.90	FFESFGDLSTPDAVMGNI	sp|P68871	0.001587	48190.9	31604.6	49783.0	81345.1	71247.7	91048.1	96561.6	80906.2	63620.8
2028	1	932.521	931.514	3.91	SAVTALWGK	sp|P68871	0.008324	10501.6	16779.6	8942.3	15011.4	24156.1	14042.1	11351.8	12300.6	17764.5
2177	5	628.142	3135.676	4.16	LLGNVLVCVLAHHFGKEF	sp|P68871	0.001987	19119.4	13757.4	19407.8	19021.5	16991.0	19759.2	31867.2	25352.2	24600.0
3183	6	707.026	4236.113	4.91	VLGAFSDGLAHLDNLKGT	sp|P68871	1.96E-08	4027.8	3990.4	5107.2	2442.7	2093.2	3049.4	56365.5	41265.3	49542.9
9954	4	829.420	3313.653	12.55	LLVVYPWTQRFFESFGDI	sp|P68871	2.22E-07	2750.5	2976.8	3055.0	6156.5	5693.5	6117.5	5801.5	6547.0	5853.1
44	3	510.584	1528.730	10.46	VGAHAGEYGAEALER	sp|P69905	0.032486	210000.6	287454.2	199090.6	248129.3	310755.7	241298.3	287942.9	309870.6	281091.9
52	4	459.229	1832.888	7.34	TYFPHFDLSHGSAQVK	sp|P69905	0.00945	224138.9	295977.9	239890.9	257702.8	288063.9	276892.6	321913.5	323653.8	391930.0
69	2	536.282	1070.550	11.80	MFLSFPTTK	sp|P69905	0.000935	105103.2	113344.4	116254.2	159777.9	198457.8	150424.2	216928.5	176247.6	164100.5
96	3	391.228	1170.662	8.79	VLSPADKTNVK	sp|P69905	8.88E-06	64999.0	61570.4	70777.1	84733.3	72979.8	78099.4	125812.0	127972.6	110992.7
239	3	611.970	1832.887	15.01	TYFPHFDLSHGSAQVK	sp|P69905	0.024113	102807.4	147151.5	105500.5	128281.9	149968.3	138280.8	144978.1	148358.3	183213.6
478	2	365.211	728.408	2.43	VLSPADK	sp|P69905	0.027337	44260.5	37337.5	44946.6	56528.9	45171.8	54034.6	49600.5	48663.9	56503.6
493	5	600.105	2995.489	14.69	VADALTNAVAHVDDMPN	sp|P69905	0.000694	54541.0	62408.6	54089.1	91571.4	73854.3	84878.0	73583.9	70358.7	69418.9
644	2	266.648	531.281	1.45	AAWGK	sp|P69905	0.00292	18064.5	14330.7	17430.6	22189.1	16758.0	20877.8	28630.2	28988.6	32694.0
727	2	626.862	1251.710	16.06	FLASVSTVLTSK	sp|P69905	0.002957	33040.3	37004.5	31689.3	48745.7	64792.5	47291.9	33641.5	30724.7	26172.6
742	3	341.856	1022.545	2.18	MFLSFPTTK	sp|P69905	0.003053	17823.8	22901.5	19513.0	19435.2	24346.8	22060.9	27890.3	27601.0	31889.7
1138	2	586.338	1170.662	13.91	VLSPADKTNVK	sp|P69905	3.84E-06	9428.9	8360.6	9212.5	12574.9	12478.6	11968.1	19398.0	20193.5	18404.5
1247	6	710.563	4257.334	10.43	LLSHCLLVTLAAHLPAEFT	sp|P69905	0.000314	91576.5	85716.0	90679.0	92382.0	79756.7	80525.7	137765.0	133061.5	123359.6
1619	5	605.734	3023.634	12.24	LLSHCLLVTLAAHLPAEFT	sp|P69905	0.000938	31044.4	28249.7	29334.9	47228.4	40933.6	43951.8	31802.3	32129.3	36421.9
1654	5	625.724	3123.582	11.42	KVADALTNAVAHVDDMPI	sp|P69905	0.002284	17606.2	17435.8	16770.9	27906.0	24945.9	27226.5	26848.5	27156.0	18150.9
1917	5	852.473	4257.329	17.02	LLSHCLLVTLAAHLPAEFT	sp|P69905	0.002979	73078.6	68074.8	71403.4	74492.8	64628.6	61435.8	112116.3	92192.7	92134.4
2407	4	756.915	3023.632	19.83	LLSHCLLVTLAAHLPAEFT	sp|P69905	0.007884	23429.2	23250.7	26486.6	41099.2	34046.7	35720.4	25918.3	29810.9	29886.8
10885	6	763.756	4576.493	9.92	LLSHCLLVTLAAHLPAEFT	sp|P69905	4.22E-08	4381.9	5330.0	4711.0	2851.9	3042.4	3143.6	16601.6	15337.6	15300.8

11189	6	843.800	5056.754	13.99	VDPVNFKLLSHCLLVTLA	sp|P69905	5.27E-05	7118.5	9052.2	8561.5	5419.2	5649.6	5401.2	8559.8	8577.1	7253.1
11638	3	540.642	1618.905	8.40	LWAYLTIEQLLEK	sp|Q06033	1.78E-05	1223.1	1116.9	1177.6	1680.7	1638.8	1964.0	2434.8	2108.8	2355.1
599	3	402.228	1203.664	7.85	FKPTLSQQQK	sp|Q14624	2.92E-06	17990.5	16929.0	17300.2	21090.9	17403.8	19065.2	34583.7	31766.6	32729.3
685	2	467.275	932.535	5.85	NVVFVIDK	sp|Q14624	0.004237	17411.5	21356.8	17084.4	17842.9	15652.3	18192.9	22524.7	24819.7	24009.9
728	3	339.855	1016.542	7.57	FAHTVVTSR	sp|Q14624	0.007277	10293.4	9004.4	10610.1	12072.1	10207.6	11551.0	14432.6	14678.5	12351.1
801	4	781.878	3123.484	15.44	FSSHVGGTLGQFYQEVL	sp|Q14624	0.001063	80215.4	51061.1	66720.9	77135.7	73626.2	87426.5	37922.9	39752.8	31279.9
807	4	485.769	1939.046	7.64	NPLVWVHASPEHVVVTR	sp|Q14624	0.040316	33342.4	45344.0	28581.0	36217.2	46867.9	36724.2	27926.4	33159.7	33899.1
885	3	805.732	2414.175	17.38	QGPVNLLSDPEQGVEVT	sp|Q14624	0.04556	62291.7	37615.2	67344.2	66083.7	45809.6	69129.3	35041.6	36754.5	34745.2
968	2	642.836	1283.658	15.06	AGFSWIEVTFK	sp|Q14624	0.000747	22160.2	22183.2	23401.2	29062.6	25517.5	29555.7	22702.1	24416.4	21974.5
975	2	654.334	1306.653	17.06	AEAQAQYSAAVAK	sp|Q14624	0.005878	26043.9	27205.1	22490.7	26033.0	30991.0	27597.8	35252.9	35767.7	30747.9
1075	3	604.656	1810.946	5.92	SPEQQETVLDGNLIIR	sp|Q14624	0.001997	21053.0	18233.2	25194.0	26232.7	25384.3	27772.0	40529.3	33042.4	34002.7
1152	2	500.283	998.552	6.73	LALDNGGLAR	sp|Q14624	0.002437	9837.2	8728.0	8304.7	9896.0	12161.1	10027.1	13426.3	13235.7	14290.9
1172	2	464.759	927.504	6.33	ILDDLSPR	sp|Q14624	0.007558	14241.9	17410.9	13405.8	16750.2	15804.6	15263.5	20446.1	18863.4	22298.1
1210	3	323.207	966.600	6.10	VRPQQLVK	sp|Q14624	0.015064	8025.9	7464.7	7897.7	9319.3	8109.6	9016.6	9503.9	9558.7	9988.5
1461	2	500.776	999.537	6.78	LALDNGGLAR	sp|Q14624	0.015831	5957.4	7826.4	7880.9	8487.5	10722.8	8767.2	10406.5	9031.9	11106.5
1472	3	552.970	1655.888	7.10	LQDRGPDVLTATVSGK	sp|Q14624	0.000766	14502.2	16497.6	14490.7	16079.1	17362.9	18269.8	12986.0	11966.8	12363.8
1576	2	906.480	1810.945	13.90	SPEQQETVLDGNLIIR	sp|Q14624	0.016069	27507.7	22362.4	29549.3	32304.7	35594.1	33595.4	46083.1	34620.2	34177.0
1610	5	723.145	3610.689	7.40	DTDRFSSHVGGTLGQFY	sp|Q14624	0.000635	31866.2	29491.2	31150.4	28673.4	23763.1	28199.5	40703.2	47256.6	47605.4
1611	3	891.443	2671.308	22.97	QGPVNLLSDPEQGVEVT	sp|Q14624	5.08E-07	24059.9	30608.4	22204.6	17614.9	20380.5	15797.4	99139.9	100804.1	84860.6
1694	3	987.849	2960.527	21.04	LWAYLTIQQLLEQTVSAS	sp|Q14624	0.018615	44432.0	40810.7	50475.9	65306.2	54494.8	58153.5	50778.9	48692.9	39175.4
1900	4	546.781	2183.096	12.80	QLGLPGPPDVPDHAAYH	sp|Q14624	0.003852	21439.9	24850.7	21905.0	22381.1	22183.1	21428.3	16891.5	16146.3	20287.7
2005	2	415.728	829.442	3.95	SIQNNVR	sp|Q14624	0.004077	12822.6	10631.5	12727.9	14227.6	11744.0	13630.3	22283.7	23393.0	25236.5
2176	3	405.892	1214.654	3.92	MNFRPGVLSSR	sp|Q14624	1.23E-05	5586.9	4868.3	5284.5	5339.1	5655.7	5066.7	9352.6	9437.8	8374.2
2263	4	741.139	2960.527	19.32	LWAYLTIQQLLEQTVSAS	sp|Q14624	0.007685	21296.1	19844.1	24099.7	29829.9	24783.4	28740.5	29927.6	28221.8	24416.5
2375	2	748.917	1495.820	16.06	ITFELVYEELLK	sp|Q14624	0.00028	16931.5	13192.1	17264.3	21902.5	20807.4	22958.6	14852.7	16010.2	15432.7
2620	5	641.946	3204.694	12.42	AGFSWIEVTFKNPLVWV	sp|Q14624	2.48E-07	12354.5	10575.6	11275.2	10945.1	8800.2	9030.5	37670.9	41887.5	39697.0
2963	4	736.160	2940.613	15.65	TGLLLLSDPDKVTIGLLF	sp|Q14624	0.000494	12815.3	12092.2	14920.4	13826.7	13592.9	11352.2	19630.9	21550.7	23683.2
3205	3	375.190	1122.548	3.47	NVHSGSTFFK	sp|Q14624	1.91E-05	2923.7	3188.1	2774.1	3393.2	3650.7	3466.0	4310.0	4594.5	4456.5
3271	4	1300.381	5197.495	16.06	RIHEDSDSALQLQDFYQE	sp|Q14624	0.002654	35479.0	25247.2	38644.3	54909.1	49244.0	53886.7	56447.5	54908.4	54505.3
3311	3	810.454	2428.340	14.91	TGLLLLSDPDKVTIGLLF	sp|Q14624	7.41E-09	17367.2	15938.6	16694.2	23384.1	22226.8	23772.2	6332.3	5667.1	5653.5
4531	5	1040.507	5197.496	17.28	RIHEDSDSALQLQDFYQE	sp|Q14624	0.00168	17400.8	13021.7	19178.6	28133.5	22994.1	24667.5	27251.0	29711.7	26555.6
4688	4	1261.356	5041.395	18.28	IHEDSDSALQLQDFYQEV	sp|Q14624	1.46E-05	21011.2	24822.4	24542.5	27684.3	27373.7	30549.8	32442.9	29334.0	29025.3
5970	3	499.614	1495.820	7.92	ITFELVYEELLK	sp|Q14624	0.000929	2762.0	2125.0	2757.3	3762.2	3390.9	3530.4	2555.5	2723.5	2822.8
6042	4	1043.543	4170.142	17.30	ILDDLSPRDQFNLIVFSTE	sp|Q14624	4.05E-07	4980.4	3996.5	4078.7	4585.7	4647.4	3448.2	37064.1	28580.8	25018.2

6425	3	800.057	2397.150	16.38	QGPVNLLSDPEQGVEVT(	sp|Q14624	0.000216	7688.6	6962.1	8900.8	9597.2	7361.4	9046.6	5035.9	3964.4	4119.8
6633	3	865.130	2592.368	17.38	LPEGSVSLIILLTDGDPTV(	sp|Q14624	1.06E-06	9945.4	7328.9	7585.7	13733.1	11277.4	13809.1	3274.1	2836.2	2865.8
13072	5	1009.286	5041.396	13.55	IHEDSDSALQLQDFYQEV	sp|Q14624	2.26E-05	3775.3	4072.8	4016.4	4988.9	4187.5	4950.8	6046.9	5599.4	5034.6
15097	5	835.036	4170.144	1.43	ILDDLSPRDQFNLIVFSTE	sp|Q14624	1.28E-07	982.1	853.7	940.8	844.2	819.3	598.0	6921.2	5694.6	5575.2
26410	3	1681.474	5041.401	12.32	IHEDSDSALQLQDFYQEV	sp|Q14624	0.001742	2483.5	2324.4	2483.3	2702.4	2549.4	3392.0	3300.8	2237.9	3230.6
29203	4	866.926	3463.675	17.58	SFAAGIQALGGTNINDAM	sp|Q14624	0.000148	399.8	436.9	492.3	1038.6	833.5	1018.7	630.8	429.3	415.5
32032	3	1155.565	3463.673	17.35	SFAAGIQALGGTNINDAM	sp|Q14624	7.57E-05	565.6	489.9	415.5	1227.4	1019.5	1270.3	258.1	411.0	412.2
35576	5	1208.813	6039.030	12.99	SFAAGIQALGGTNINDAM	sp|Q14624	0.000794	561.5	518.2	607.0	512.7	336.7	569.7	1487.0	1093.8	1272.9
23997	3	961.183	2880.528	19.90	EYGVVLAPDGSTVAVEPL	sp|Q96PD5	0.002565	1124.6	871.0	1002.6	1701.6	1510.0	1535.0	727.1	367.4	255.7
3840	2	730.382	1458.750	16.06	FGQGSGPIVLDDVR	sp|Q9UGM	9.13E-08	16.0	40.1	44.4	148.4	102.2	139.8	32908.2	31938.8	29266.2

Supplementary Table S2. The 80 WGA-enriched plasma proteins selected for protein quantification

UniProt	Gene	ID	Description	Peptides used	Non-metastatic CRC vs Healthy control						Metastatic CRC vs Healthy control					
					NM1/ Avg Ctr	NM2/ Avg Ctr	NM2/ Avg Ctr	LOG 10 p value	*p*-value	Fold change (NM/Ctr)	M1/ Avg Ctr	M2/ Avg Ctr	M3/ Avg Ctr	LOG 10 p value	*p*-value	Fold change (M/Ctr)
sp|Q9UGM3	DMBT1	DMBT1_HUMAN	Deleted in malignant brain tumors 1 protein	1	4.43	3.05	4.17	1.6806	0.0209	3.88	1.15	1.22	1.15	2.9189	0.0012	1.17
sp|P01833	PIGR	PIGR_HUMAN	Polymeric immunoglobulin receptor	1	2.07	1.87	1.48	1.3583	0.0438	1.80	-1.35	-1.36	-1.28	2.1955	0.0064	-1.33
sp|P04003	C4BPA	C4BPA_HUMAN	C4b-binding protein alpha chain	1	1.36	1.00	1.24	0.7081	>0.05	1.20	1.24	1.22	1.21	1.7400	0.0182	1.23
sp|Q06033	ITIH3	ITIH3_HUMAN	Inter-alpha-trypsin inhibitor heavy chain H3	1	1.43	1.40	1.68	1.5403	0.0288	1.50	1.43	1.49	1.42	2.1251	0.0075	1.44
sp|P08603	CFH	CFAH_HUMAN	Complement factor H	1	1.02	1.18	1.01	0.5018	>0.05	1.07	1.61	1.62	1.59	2.3020	0.0050	1.60
sp|P00736	C1R	C1R_HUMAN	Complement C1r subcomponent	1	1.81	1.19	1.39	0.9024	>0.05	1.47	1.02	-1.10	-1.09	1.4201	0.0380	-0.39
sp|P02042	HBD	HBD_HUMAN	Hemoglobin subunit delta	1	1.06	1.60	1.11	0.5557	>0.05	1.25	-1.71	-1.62	-1.76	1.6317	0.0233	-1.70
sp|O75882	ATRN	ATRN_HUMAN	Attractin	1	-1.22	-1.22	-1.20	5.0120	<0.0001	-1.21	-1.08	-1.18	-1.10	0.6718	>0.05	-1.12
sp|P01714	IGLV3-19	LV319_HUMAN	Immunoglobulin lambda variable 3-19	1	-1.01	-1.40	-1.19	2.5872	0.0026	-1.20	20.85	18.60	24.18	0.3632	>0.05	21.21
sp|P06276	BCHE	CHLE_HUMAN	Cholinesterase	1	1.00	1.04	1.00	0.4855	>0.05	1.01	-1.15	-1.15	-1.21	2.9835	0.0010	-1.17
sp|P05546	SERPIND1	HEP2_HUMAN	Heparin cofactor 2	1	1.08	-1.05	-1.02	0.7001	>0.05	-0.33	1.02	1.07	-1.04	3.6544	0.0002	0.35
sp|P00748	F12	FA12_HUMAN	Coagulation factor XII	1	1.21	1.17	1.12	1.6106	0.0245	1.16	1.10	1.06	1.10	2.4333	0.0037	1.09
sp|O75636	FCN3	FCN3_HUMAN	Ficolin-3	1	1.11	1.06	1.10	1.4551	0.0351	1.09	-2.05	-2.10	-2.00	3.2078	0.0006	-2.05
sp|A0A075B6H9	IGLV4-69	LV469_HUMAN	Immunoglobulin lambda variable 4-69	1	1.24	-1.02	1.17	0.2645	>0.05	0.46	1.60	1.53	1.66	2.9170	0.0012	1.60
sp|P27169	PON1	PON1_HUMAN	Serum paraoxonase/arylesterase 1	1	1.30	1.22	1.27	2.0805	0.0083	1.26	-1.79	-2.01	-2.02	3.7121	0.0002	-1.94
sp|O75460	ERN1	ERN1_HUMAN	Serine/threonine-protein kinase/endoribonuclease IRE1	1	1.53	1.42	1.72	1.6264	0.0236	1.56	1.30	1.21	1.25	3.1571	0.0007	1.25
sp|P01817		HV205_HUMAN	Immunoglobulin heavy variable 2-5	1	-1.07	-1.37	-1.16	2.8053	0.0016	-1.20	-1.15	-1.18	-1.32	2.7597	0.0017	-1.21
sp|Q96PD5	PGLYRP2	PGRP2_HUMAN	N-acetylmuramoyl-L-alanine amidase	1	1.70	1.51	1.54	1.9797	0.0105	1.58	1.34	1.30	1.28	1.4248	0.0376	1.30
sp|P06681	C2	CO2_HUMAN	Complement C2	2	1.04	-1.24	1.07	0.3459	>0.05	0.29	1.39	1.47	1.38	3.6505	0.0002	1.42
sp|P02774	GC	VTDB_HUMAN	Vitamin D-binding protein	2	-1.42	-1.99	-1.71	2.4406	0.0036	-1.71	1.51	1.53	1.38	1.9913	0.0102	1.47
sp|P06310	IGKV2-30	KV230_HUMAN	Immunoglobulin kappa variable 2-30	2	-1.22	-1.19	-1.09	3.4605	0.0003	-1.17	-1.37	-2.72	-3.91	0.8236	>0.05	-2.67
sp|P00739	HPR	HPTR_HUMAN	Haptoglobin-related protein	2	-1.03	1.31	1.09	0.2664	>0.05	0.46	-1.46	-1.40	-1.43	0.2515	>0.05	-1.43
sp|P25311	AZGP1	ZA2G_HUMAN	Zinc-alpha-2-glycoprotein	2	-1.35	-1.61	-1.30	2.7877	0.0016	-1.42	-1.03	-1.09	1.05	0.7114	>0.05	-0.36
sp|O43866	CD5L	CD5L_HUMAN	CD5 antigen-like	2	-1.06	-1.15	-1.18	3.4928	0.0003	-1.13	1.20	1.15	1.23	0.7263	>0.05	1.19
sp|P02753	RBP4	RET4_HUMAN	Retinol-binding protein 4	2	1.26	1.17	1.24	1.8320	0.0147	1.22	-1.88	-1.80	-1.95	3.9514	0.0001	-1.87
sp|P06312	IGKV4-1	KV401_HUMAN	Immunoglobulin kappa variable 4-1	2	-1.15	1.01	-1.08	0.7329	>0.05	-0.41	1.05	1.07	1.01	3.6891	0.0002	1.05
sp|P02787	TF	TRFE_HUMAN	Serotransferrin	2	-1.26	-1.34	-1.24	3.8060	0.0002	-1.28	-1.77	-1.89	-1.90	3.6705	0.0002	-1.85
sp|P01877	IGHA2	IGHA2_HUMAN	Immunoglobulin heavy constant alpha 2	3	-1.12	-1.19	-1.11	3.9034	0.0001	-1.14	-2.26	-2.27	-2.69	2.3654	0.0043	-2.41
sp|P01860	IGHG3	IGHG3_HUMAN	Immunoglobulin heavy constant gamma 3	3	-1.54	-1.66	-1.62	3.7221	0.0002	-1.61	1.12	1.15	1.45	3.2581	0.0006	1.24
sp|P02748	C9	CO9_HUMAN	Complement component C9	4	1.92	1.52	1.63	1.5555	0.0278	1.69	-1.36	-1.37	-1.57	1.8598	0.0138	-1.43
sp|P0C0L5	C4B	CO4B_HUMAN	Complement C4-B	4	1.61	1.35	1.49	1.6373	0.0231	1.48	1.47	1.42	1.31	2.3035	0.0050	1.40
sp|P00751	CFB	CFAB_HUMAN	Complement factor B	4	-1.07	-1.10	-1.17	3.6899	0.0002	-1.11	1.31	1.52	1.29	1.8904	0.0129	1.37
sp|P22792	CPN2	CPN2_HUMAN	Carboxypeptidase N subunit 2	4	-1.04	-1.27	-1.19	3.0374	0.0009	-1.17	-1.08	-1.18	-1.10	3.7174	0.0002	-1.12
sp|P08185	SERPINA6	CBG_HUMAN	Corticosteroid-binding globulin	4	1.08	-1.23	1.05	0.3409	>0.05	0.30	-1.45	-1.53	-1.27	3.1342	0.0007	-1.42
sp|P08697	SERPINF2	A2AP_HUMAN	Alpha-2-antiplasmin	4	-1.17	-1.19	-1.27	3.6782	0.0002	-1.21	1.70	1.64	1.81	3.3709	0.0004	1.71
sp|P0C0L4	C4A	CO4A_HUMAN	Complement C4-A	5	2.22	1.65	2.28	1.4556	0.0350	2.05	1.78	1.94	1.62	3.6756	0.0002	1.78
sp|P01861	IGHG4	IGHG4_HUMAN	Immunoglobulin heavy constant gamma 4	5	-1.41	-1.82	-1.52	2.6408	0.0023	-1.58	-1.38	-1.30	-1.41	3.2839	0.0005	-1.36
sp|P02751	FN1	FINC_HUMAN	Fibronectin	5	-1.44	-1.69	-1.51	3.0557	0.0009	-1.55	1.63	1.52	1.69	3.9954	0.0001	1.61
sp|P01031	C5	CO5_HUMAN	Complement C5	6	1.53	1.39	1.61	1.7824	0.0165	1.51	1.50	1.50	1.62	1.6814	0.0208	1.54
sp|P02743	APCS	SAMP_HUMAN	Serum amyloid P-component	6	1.10	1.10	1.11	3.0010	0.0010	1.11	-2.62	-2.68	-2.68	4.5590	<0.0001	-2.66
sp|P01859	IGHG2	IGHG2_HUMAN	Immunoglobulin heavy constant gamma 2	6	-1.24	-1.26	-1.25	5.0495	<0.0001	-1.25	1.04	1.05	-1.06	3.3132	0.0005	0.34

sp|P02766	TTR	TTHY_HUMAN	Transthyretin	6	1.03	1.04	1.00	0.7072	>0.05	1.02	-1.32	-1.41	-1.40	3.8191	0.0002	-1.37
sp|P35858	IGFALS	ALS_HUMAN	Insulin-like growth factor-binding protein complex acid labile subunit	6	-1.20	-1.27	-1.23	4.0780	0.0001	-1.23	-1.64	-1.72	-1.76	3.6588	0.0002	-1.71
sp|P01591	JCHAIN	IGJ_HUMAN	Immunoglobulin J chain	7	-1.16	-1.09	-1.17	3.8530	0.0001	-1.14	-2.10	-2.09	-1.96	1.9099	0.0123	-2.05
sp|P04004	VTN	VTNC_HUMAN	Vitronectin	7	-1.09	-1.03	-1.06	4.1237	0.0001	-1.06	-1.17	-1.17	-1.21	4.5078	<0.0001	-1.18
sp|P01019	AGT	ANGT_HUMAN	Angiotensinogen	7	-1.18	-1.39	-1.30	3.1245	0.0008	-1.29	-1.24	-1.27	-1.22	3.7820	0.0002	-1.25
sp|P10909	CLU	CLUS_HUMAN	Clusterin	8	-1.05	1.09	-1.03	0.6967	>0.05	-0.33	-1.04	-1.02	-1.12	1.8484	0.0142	-1.06
sp|P01834	IGKC	IGKC_HUMAN	Immunoglobulin kappa constant	9	-1.01	1.06	-1.02	0.7058	>0.05	-0.32	1.06	1.04	1.00	0.3490	>0.05	1.03
sp|P04217	A1BG	A1BG_HUMAN	Alpha-1B-glycoprotein	9	-1.31	-1.12	-1.36	2.9758	0.0011	-1.26	-2.00	-1.95	-1.97	3.6628	0.0002	-1.98
sp|P01857	IGHG1	IGHG1_HUMAN	Immunoglobulin heavy constant gamma 1	9	-1.25	-1.18	-1.20	4.1486	0.0001	-1.21	1.16	1.15	1.21	4.3015	<0.0001	1.17
sp|P02760	AMBP	AMBP_HUMAN	Protein AMBP	10	1.36	1.38	1.40	3.1674	0.0007	1.38	-1.04	-1.04	-1.01	2.4293	0.0037	-1.03
sp|P19652	ORM2	A1AG2_HUMAN	Alpha-1-acid glycoprotein 2	12	1.14	1.10	1.06	0.3826	0.0334	1.10	-1.66	-1.70	-1.97	0.3324	>0.05	-1.78
sp|P04114	APOB	APOB_HUMAN	Apolipoprotein B-100	12	1.20	1.29	1.14	1.4087	0.0390	1.21	2.43	2.48	3.14	4.3344	<0.0001	2.68
sp|P04196	HRG	HRG_HUMAN	Histidine-rich glycoprotein	13	1.16	1.05	1.19	1.0474	>0.05	1.13	-1.29	-1.23	-1.40	0.7782	>0.05	-1.31
sp|P02750	LRG1	A2GL_HUMAN	Leucine-rich alpha-2-glycoprotein	14	1.29	1.23	1.33	2.0089	0.0098	1.28	1.14	1.16	1.12	4.4624	<0.0001	1.14
sp|P02763	ORM1	A1AG1_HUMAN	Alpha-1-acid glycoprotein 1	14	1.18	1.41	1.19	1.1401	>0.05	1.26	-1.01	1.05	1.10	2.2969	0.0050	0.38
sp|P68871	HBB	HBB_HUMAN	Hemoglobin subunit beta	15	1.20	1.33	1.22	1.5893	0.0257	1.25	1.30	1.35	1.29	2.2000	0.0063	1.31
sp|P05155	SERPING1	IC1_HUMAN	Plasma protease C1 inhibitor	15	1.24	1.32	1.18	1.6132	0.0244	1.25	-1.09	-1.10	-1.24	0.9416	>0.05	-1.14
sp|P01042	KNG1	KNG1_HUMAN	Kininogen-1	16	1.11	1.07	1.08	1.7536	0.0176	1.09	1.11	1.09	1.02	2.6543	0.0022	1.07
sp|P69905	HBA1	HBA_HUMAN	Hemoglobin subunit alpha	18	1.18	1.25	1.15	1.6009	0.0251	1.19	1.38	1.24	1.42	3.1304	0.0007	1.35
sp|P00734	F2	THRB_HUMAN	Prothrombin	18	1.18	1.29	1.14	1.3545	0.0442	1.20	-1.05	-1.08	-1.04	2.4916	0.0032	-1.05
sp|P01876	IGHA1	IGHA1_HUMAN	Immunoglobulin heavy constant alpha 1	18	-1.13	-1.28	-1.18	3.4045	0.0004	-1.20	-1.19	-1.21	-1.16	0.3482	>0.05	-1.19
sp|P01008	SERPINC1	ANT3_HUMAN	Antithrombin-III	19	-1.29	-1.21	-1.26	3.9674	0.0001	-1.25	1.05	1.02	1.16	4.5439	<0.0001	1.08
sp|P43652	AFM	AFAM_HUMAN	Afamin	19	-1.25	-1.21	-1.26	4.3004	0.0001	-1.24	-2.39	-2.56	-2.63	4.6107	<0.0001	-2.53
sp|P19827	ITIH1	ITIH1_HUMAN	Inter-alpha-trypsin inhibitor heavy chain H1	20	1.22	1.20	1.14	1.7732	0.0169	1.19	2.08	1.80	2.01	1.2260	>0.05	1.96
sp|P19823	ITIH2	ITIH2_HUMAN	Inter-alpha-trypsin inhibitor heavy chain H2	22	1.20	1.14	1.18	1.9533	0.0111	1.17	1.35	1.32	1.26	0.7576	>0.05	1.31
sp|P02765	AHSG	FETUA_HUMAN	N Alpha-2-HS-glycoprotein	23	1.18	1.05	1.16	1.0702	>0.05	1.13	-1.80	-1.84	-1.62	0.8860	>0.05	-1.75
sp|P01011	SERPINA3	AACT_HUMAN	Alpha-1-antichymotrypsin	24	1.30	1.24	1.22	2.0698	0.0085	1.25	-1.31	-1.35	-1.24	2.2267	0.0059	-1.30
sp|P02671	FGA	FIBA_HUMAN	Fibrinogen alpha chain	32	-1.28	-1.31	-1.30	4.7869	<0.0001	-1.30	1.94	2.00	2.12	1.7400	0.0182	2.02
sp|P02679	FGG	FIBG_HUMAN	Fibrinogen gamma chain	32	-1.25	-1.27	-1.25	5.0410	<0.0001	-1.26	1.40	1.37	1.40	1.5949	0.0254	1.39
sp|P01871	IGHM	IGHM_HUMAN	Immunoglobulin heavy constant mu	34	-1.26	-1.21	-1.28	4.0846	0.0001	-1.25	1.04	-1.08	1.00	4.4724	<0.0001	0.32
sp|P02675	FGB	FIBB_HUMAN	Fibrinogen beta chain	37	-1.40	-1.42	-1.33	3.9636	0.0001	-1.38	1.19	1.28	-1.09	2.0217	0.0095	0.46
sp|P01009	SERPINA1	A1AT_HUMAN	Alpha-1-antitrypsin	37	1.03	1.10	1.00	0.5353	>0.05	1.04	1.10	1.04	1.09	4.4053	<0.0001	1.08
sp|Q14624	ITIH4	ITIH4_HUMAN	Inter-alpha-trypsin inhibitor heavy chain H4	40	1.20	1.11	1.19	1.5999	0.0251	1.17	1.74	1.75	1.74	2.1681	0.0068	1.74
sp|P02790	HPX	HEMO_HUMAN	Hemopexin	45	1.46	1.46	1.46	4.6672	<0.0001	1.46	1.12	1.05	1.02	2.3839	0.0041	1.07
sp|P00738	HP	HPT_HUMAN	Haptoglobin	60	1.04	1.14	1.04	0.8076	>0.05	1.07	1.30	1.24	1.24	2.5493	0.0028	1.26
sp|P02768	ALB	ALBU_HUMAN	Serum albumin	63	-1.42	-1.32	-1.81	2.4520	0.0035	-1.52	-1.50	-1.50	-1.51	4.4652	<0.0001	-1.51
sp|P00450	CP	CERU_HUMAN	Ceruloplasmin	82	1.37	1.41	1.33	2.4194	0.0038	1.37	-1.55	-1.58	-2.06	2.7328	0.0019	-1.73
sp|P01024	C3	CO3_HUMAN	Complement C3	128	1.40	1.38	1.38	3.7168	0.0002	1.39	1.14	1.14	1.09	4.0388	0.0001	1.12
sp|P01023	A2M	A2MG_HUMAN	Alpha-2-macroglobulin	168	1.02	1.00	-1.01	0.3699	>0.05	0.33	-1.32	-1.36	-1.37	7.6089	<0.0001	-1.35

Supplementary Table S3. List of the 62 WGA-enriched plasma proteins containing at least 2 peptides and their fold change in non-metastatic and metastatic CRC patients compared to those of the healthy controls

					Non-metastatic CRC vs Healthy control		Metastatic CRC vs Healthy control	
**UniProt**	**Gene**	**ID**	**Description**	**Peptides used**	***p*-value**	**Fold change (NM/Ctr)**	***p*-value**	**Fold change (M/Ctr)**
sp|P00738	HP	HPT_HUMAN	Haptoglobin	60	>0.05	1.07	0.0028	2.02
sp|P02748	C9	CO9_HUMAN	Complement component C9	4	0.0278	1.69	0.0138	1.78
sp|P02750	LRG1	A2GL_HUMAN	Leucine-rich alpha-2-glycoprotein	14	0.0098	1.28	0.0000	1.74
sp|P02763	ORM1	A1AG1_HUMAN	Alpha-1-acid glycoprotein 1	14	>0.05	1.26	0.0050	1.71
sp|P68871	HBB	HBB_HUMAN	Hemoglobin subunit beta	15	0.0257	1.25	0.0063	1.61
sp|P06681	C2	CO2_HUMAN	Complement C2	2	>0.05	0.29	0.0002	1.60
sp|P02760	AMBP	AMBP_HUMAN	Protein AMBP	10	0.0007	1.38	0.0037	1.60
sp|P02774	GC	VTDB_HUMAN	Vitamin D-binding protein	2	0.0036	-1.71	0.0102	1.47
sp|P01042	KNG1	KNG1_HUMAN	Kininogen-1	16	0.0176	1.09	0.0022	1.44
sp|P0C0L5	C4B	CO4B_HUMAN	Complement C4-B	4	0.0231	1.48	0.0050	1.42
sp|P00751	CFB	CFAB_HUMAN	Complement factor B	4	0.0002	-1.11	0.0129	1.40
sp|P69905	HBA1	HBA_HUMAN	Hemoglobin subunit alpha	18	0.0251	1.19	0.0007	1.39
sp|P02790	HPX	HEMO_HUMAN	Hemopexin	45	<0.0001	1.46	0.0041	1.31
sp|Q14624	ITIH4	ITIH4_HUMAN	Inter-alpha-trypsin inhibitor heavy chain H4	40	0.0251	1.17	0.0068	1.31
sp|P00734	F2	THRB_HUMAN	Prothrombin	18	0.0442	1.20	0.0032	1.30
sp|P01877	IGHA2	IGHA2_HUMAN	Immunoglobulin heavy constant alpha 2	3	0.0001	-1.14	0.0043	1.26
sp|P02675	FGB	FIBB_HUMAN	Fibrinogen beta chain	37	0.0001	-1.38	0.0095	1.25
sp|P06310	IGKV2-30	KV230_HUMAN	Immunoglobulin kappa variable 2-30	2	0.0003	-1.17	>0.05	1.24
sp|P00450	CP	CERU_HUMAN	Ceruloplasmin	82	0.0038	1.37	0.0019	1.23
sp|P10909	CLU	CLUS_HUMAN	Clusterin	8	>0.05	-0.33	0.0142	1.19
sp|P01591	JCHAIN	IGJ_HUMAN	Immunoglobulin J chain	7	0.0001	-1.14	0.0123	1.17
sp|P02671	FGA	FIBA_HUMAN	Fibrinogen alpha chain	32	<0.0001	-1.30	0.0182	1.17
sp|P01011	SERPINA3	AACT_HUMAN	Alpha-1-antichymotrypsin	24	0.0085	1.25	0.0059	1.14
sp|P01031	C5	CO5_HUMAN	Complement C5	6	0.0165	1.51	0.0208	1.12
sp|P02679	FGG	FIBG_HUMAN	Fibrinogen gamma chain	32	<0.0001	-1.26	0.0254	1.09
sp|P19827	ITIH1	ITIH1_HUMAN	Inter-alpha-trypsin inhibitor heavy chain H1	20	0.0169	1.19	>0.05	1.08
sp|P02765	AHSG	FETUA_HUMAN	N Alpha-2-HS-glycoprotein	23	>0.05	1.13	>0.05	1.07
sp|P04196	HRG	HRG_HUMAN	Histidine-rich glycoprotein	13	>0.05	1.13	>0.05	1.07
sp|P05155	SERPING1	IC1_HUMAN	Plasma protease C1 inhibitor	15	0.0244	1.25	>0.05	1.05
sp|P19823	ITIH2	ITIH2_HUMAN	Inter-alpha-trypsin inhibitor heavy chain H2	22	0.0111	1.17	>0.05	1.03
sp|P00739	HPR	HPTR_HUMAN	Haptoglobin-related protein	2	>0.05	0.46	>0.05	0.46
sp|P19652	ORM2	A1AG2_HUMAN	Alpha-1-acid glycoprotein 2	12	0.0334	1.10	>0.05	0.38
sp|P01876	IGHA1	IGHA1_HUMAN	Immunoglobulin heavy constant alpha 1	18	0.0004	-1.20	>0.05	0.35
sp|P01834	IGKC	IGKC_HUMAN	Immunoglobulin kappa constant	9	>0.05	-0.32	>0.05	0.34
sp|P25311	AZGP1	ZA2G_HUMAN	Zinc-alpha-2-glycoprotein	2	0.0016	-1.42	>0.05	-0.36
sp|O43866	CD5L	CD5L_HUMAN	CD5 antigen-like	2	0.0003	-1.13	>0.05	-0.39
sp|P01008	SERPINC1	ANT3_HUMAN	Antithrombin-III	19	0.0001	-1.25	<0.0001	-1.03
sp|P02743	APCS	SAMP_HUMAN	Serum amyloid P-component	6	0.0010	1.11	<0.0001	-1.05

sp|P0C0L4	C4A	CO4A_HUMAN	Complement C4-A	5	0.0350	2.05	<0.0001	-1.06
sp|P22792	CPN2	CPN2_HUMAN	Carboxypeptidase N subunit 2	4	0.0009	-1.17	<0.0001	-1.12
sp|P01861	IGHG4	IGHG4_HUMAN	Immunoglobulin heavy constant gamma 4	5	0.0023	-1.58	<0.0001	-1.14
sp|P01024	C3	CO3_HUMAN	Complement C3	128	0.0002	1.39	<0.0001	-1.17
sp|P04004	VTN	VTNC_HUMAN	Vitronectin	7	0.0001	-1.06	<0.0001	-1.18
sp|P01871	IGHM	IGHM_HUMAN	Immunoglobulin heavy constant mu	34	0.0001	-1.25	<0.0001	-1.19
sp|P01860	IGHG3	IGHG3_HUMAN	Immunoglobulin heavy constant gamma 3	3	0.0002	-1.61	<0.0001	-1.21
sp|P04114	APOB	APOB_HUMAN	Apolipoprotein B-100	12	0.0390	1.21	<0.0001	-1.25
sp|P04217	A1BG	A1BG_HUMAN	Alpha-1B-glycoprotein	9	0.0011	-1.26	<0.0001	-1.30
sp|P01859	IGHG2	IGHG2_HUMAN	Immunoglobulin heavy constant gamma 2	6	<0.0001	-1.25	<0.0001	-1.31
sp|P02753	RBP4	RET4_HUMAN	Retinol-binding protein 4	2	0.0147	1.22	<0.0001	-1.33
sp|P01009	SERPINA1	A1AT_HUMAN	Alpha-1-antitrypsin	37	>0.05	1.04	<0.0001	-1.35
sp|P06312	IGKV4-1	KV401_HUMAN	Immunoglobulin kappa variable 4-1	2	>0.05	-0.41	<0.0001	-1.36
sp|P02766	TTR	TTHY_HUMAN	Transthyretin	6	>0.05	1.02	<0.0001	-1.37
sp|P01857	IGHG1	IGHG1_HUMAN	Immunoglobulin heavy constant gamma 1	9	0.0001	-1.21	<0.0001	-1.43
sp|P08185	SERPINA6	CBG_HUMAN	Corticosteroid-binding globulin	4	>0.05	0.30	0.001	-1.43
sp|P01023	A2M	A2MG_HUMAN	Alpha-2-macroglobulin	168	>0.05	0.33	<0.0001	-1.51
sp|P01019	AGT	ANGT_HUMAN	Angiotensinogen	7	0.0008	-1.29	<0.0001	-1.71
sp|P02787	TF	TRFE_HUMAN	Serotransferrin	2	0.0002	-1.28	<0.0001	-1.87
sp|P43652	AFM	AFAM_HUMAN	Afamin	19	0.0001	-1.24	<0.0001	-1.98
sp|P02751	FN1	FINC_HUMAN	Fibronectin	5	0.0009	-1.55	<0.0001	-2.05
sp|P35858	IGFALS	ALS_HUMAN	Insulin-like growth factor-binding protein comple	6	0.0001	-1.23	<0.0001	-2.05
sp|P08697	SERPINF2	A2AP_HUMAN	Alpha-2-antiplasmin	4	0.0002	-1.21	<0.0001	-2.53
sp|P02768	ALB	ALBU_HUMAN	Serum albumin	63	0.0035	-1.52	<0.0001	-2.66

Supplementary Table S4. N-linked glycosylated searched against 20 WGA-enriched glycoproteins of the pooled sample of healthy control, non-metastatic and metastatic CRC patient groups (criteria: DeltaMod > 20, Log Prob > 1.3 (p-value < 0.05) and MS/MS fragmentations containing modifications were found in at least 2 of 3 runs)

Description	N-glycosylation	Healthy control		Non-metastatic CRC		Metastatic CRC	
		Delta Mod	|Log Prob|	Delta Mod	|Log Prob|	Delta Mod	|Log Prob|
>sp|P01023|A2MG_HUMAN Alpha-2-macroglobulin OS=Homo sapiens OX=9606 GN=A2M PE=1 SV=3							
K.GCVLLSYLN[55][+2204.772]ETVTVSASLESVR.G	HexNAc(4)Hex(5)NeuAc(2)	48.33	4.00	-	-	-	-
K.GCVLLSYLN[55][+2350.830]ETVTVSASLESVR.G	HexNAc(4)Hex(5)Fuc(1)NeuAc(2)	33.45	4.73	-	-	-	-
R.GN[70][+2204.772]R.S	HexNAc(4)Hex(5)NeuAc(2)	-	-	116.01	2.17	-	-
K.IITILEEEMN[247][+2018.708]VSVCGLYTYGKPVPGHVTVSICR.K	HexNAc(3)Hex(6)Fuc(1)NeuAc(1)	-	-	383.31	8.27	-	-
K.IITILEEEMN[247] [+2262.814]VSVCGLYTYGKPVPGHVTVSICR.K	HexNAc(5)Hex(5)Fuc(1)NeuAc(1)	102.42	3.35	-	-	-	-
K.VSN[1424][+1913.677]QTLSLFFTVLQDVPVR.D	HexNAc(4)Hex(5)NeuAc(1)	189.17	3.92	151.31	3.70	-	-
K.VSN[1424] [+2059.735]QTLSLFFTVLQDVPVR.D	HexNAc(4)Hex(5)Fuc(1)NeuAc(1)	154.10	4.04	161.60	4.13	-	-
K.VSN[1424] [+2226.754]QTLSLFFTVLQDVPVR.D	HexNAc(4)Hex(5)NeuAc(2)Na(1)	-	-	81.68	3.07	-	-
K.VSN[1424] [+2569.905]QTLSLFFTVLQDVPVR.D	HexNAc(5)Hex(6)NeuAc(2)	40.34	2.76	-	-	-	-
>sp|P00738|HPT_HUMAN Haptoglobin OS=Homo sapiens OX=9606 GN=HP PE=1 SV=1							
K.MVSHHN[184][+1913.677]LTTGATLINEQWLLTTAK.N	HexNAc(4)Hex(5)NeuAc(1)	145.40	3.62	156.52	2.96	200.20	2.60
K.MVSHHN[184] [+2018.708]LTTGATLINEQWLLTTAK.N	HexNAc(3)Hex(6)Fuc(1)NeuAc(1)	129.34	3.42	56.23	3.18	151.24	3.06
K.MVSHHN[184] [+2204.772]LTTGATLINEQWLLTTAK.N	HexNAc(4)Hex(5)NeuAc(2)	23.91	3.15	46.94	2.54	20.55	2.70
K.MVSHHN[184] [+2861.000]LTTGATLINEQWLLTTAK.N	HexNAc(5)Hex(6)NeuAc(3)	-	-	47.68	3.48	-	-
K.VVLHPN[241][+1913.677]YSQVDIGLIK.L	HexNAc(4)Hex(5)NeuAc(1)	166.98	4.02	37.92	3.51	53.89	3.68
K.VVLHPN[241] [+2204.772]YSQVDIGLIK.L	HexNAc(4)Hex(5)NeuAc(2)	33.27	2.32	46.13	3.64	35.92	2.85
K.VVLHPN[241] [+2226.754]YSQVDIGLIK.L	HexNAc(4)Hex(5)NeuAc(2)Na(1)	-	-	98.82	2.95	-	-
K.VVLHPN[241] [+2278.809]YSQVDIGLIK.L	HexNAc(5)Hex(6)NeuAc(1)	-	-	-	-	47.30	3.18

>sp|P02763|A1AG1_HUMAN Alpha-1-acid glycoprotein 1 OS=Homo sapiens OX=9606 GN=ORM1 PE=1 SV=1							
R.NEEYN^{56}[+2204.772]K.S	HexNAc(4)Hex(5)NeuAc(2)	160.38	2.78	200.79	2.43	198.73	2.55
R.QDQCIYN^{93}[+2861.000]TTYLNVQR.E	HexNAc(5)Hex(6)NeuAc(3)	-	-	59.58	3.50	21.86	2.19
>sp|P0C0L4|CO4A_HUMAN Complement C4-A OS=Homo sapiens OX=9606 GN=C4A PE=1 SV=2							
R.GLN^{1328}[+2204.772]VTLSSTGR.N	HexNAc(4)Hex(5)NeuAc(2)	-	-	-	-	185.59	3.48

Supplementary Table S5. Glycated peptides searched against 20 WGA-enriched glycoproteins of the pooled sample of healthy control, non-metastatic and metastatic CRC patient groups (criteria: DeltaMod > 20, Log Prob > 1.3 (p-value < 0.05) and MS/MS fragmentations containing modifications were found in at least 2 of 3 runs)

Peptide sequence	Modification	Healthy control		Non-metastatic CRC		Metastatic CRC	
		Delta Mod	|Log Prob|	Delta Mod	|Log Prob|	Delta Mod	|Log Prob|
>sp|P01023|A2MG_HUMAN Alpha-2-macroglobulin OS=Homo sapiens OX=9606 GN=A2M PE=1 SV=3							
R.AVDQSVLLMK^{608}[+58.005]PDAELSASSVYNLLPEKDLTGFPGPLNDQDNEDCINR.H	CML	73.56	6.58	74.81	6.42	78.67	6.56
K.DTVIK^{901}[+58.005]PLLVEPEGLEKETTFNSLLCPSGGEVSEELSLK.L	CML	22.30	2.69	126.19	5.80	-	-
K.DTVIKPLLVEPEGLEK^{912}[+58.005]ETTFNSLLCPSGGEVSEELSLK.L	CML	83.46	2.73	-	-	-	-

Note: CML= carboxymethyllysine
